# MicroRNA-7 regulates melanocortin circuits involved in mammalian energy homeostasis

**DOI:** 10.1038/s41467-022-33367-w

**Published:** 2022-09-29

**Authors:** Mary P. LaPierre, Katherine Lawler, Svenja Godbersen, I. Sadaf Farooqi, Markus Stoffel

**Affiliations:** 1 Institute of Molecular Health Sciences, ETH Zürich, 8093 Zürich, Switzerland; 2 University of Cambridge Metabolic Research Laboratories and NIHR Cambridge Biomedical Research Centre, Wellcome Trust-MRC Institute of Metabolic Science, Addenbrooke’s Hospital, Cambridge, CB2 0QQ UK; 3 Medical Faculty, University of Zürich, 8091 Zürich, Switzerland

**Keywords:** Obesity, Hypothalamus, miRNAs

## Abstract

MicroRNAs (miRNAs) modulate physiological responses by repressing the expression of gene networks. We found that global deletion of microRNA-7 (miR-7), the most enriched miRNA in the hypothalamus, causes obesity in mice. Targeted deletion of miR-7 in Single-minded homolog 1 (Sim1) neurons, a critical component of the hypothalamic melanocortin pathway, causes hyperphagia, obesity and increased linear growth, mirroring Sim1 and Melanocortin-4 receptor (MC4R) haplo-insufficiency in mice and humans. We identified *Snca* (α-Synuclein) and *Igsf8* (Immunoglobulin Superfamily Member 8) as miR-7 target genes that act in Sim1 neurons to regulate body weight and endocrine axes. In humans, *MIR-7-1* is located in the last intron of *HNRNPK*, whose promoter drives the expression of both genes. Genetic variants at the *HNRNPK* locus that reduce its expression are associated with increased height and truncal fat mass. These findings demonstrate that miR-7 suppresses gene networks involved in the hypothalamic melanocortin pathway to regulate mammalian energy homeostasis.

## Abstract (web summary)

The hypothalamus controls systemic energy homeostasis. Here, the authors show that loss of microRNA-7 in hypothalamic neurons causes obesity, increased growth and endocrine dysfunction in mice, and report genetic evidence that links microRNA-7 to height and adiposity in humans.

## Introduction

Energy homeostasis is maintained by neurons in the hypothalamus that modulate eating behaviour, pituitary hormone secretion, and autonomic nervous system activation in response to changes in nutrient availability^1,2^. Hypothalamic circuits involving leptin-responsive neurons in the arcuate nucleus (ARC) that express Pro-opiomelanocortin (POMC) and Agouti-related protein (AgRP) play a pivotal role in energy homeostasis and the defence against starvation in mice and humans^3^. These ARC neurons synapse onto Melanocortin-4 receptor (MC4R)-expressing neurons in the paraventricular nucleus of the hypothalamus (PVN). POMC-derived peptides are endogenous agonists of MC4R, whose activation leads to reduced food intake and increased energy expenditure in the fed state. In the fasted state, AgRP (an endogenous MC4R antagonist) drives an increase in food intake^4^.

Single-minded homology 1 (SIM1) is a basic helix-loop-helix transcription factor required for the migration and differentiation of PVN neurons, which express MC4R and the neuropeptides oxytocin (OXT), arginine vasopressin (AVP), corticotropin-releasing hormone (CRH), thyrotropin-releasing hormone (TRH), and somatostatin^5^. These neurons project to the posterior pituitary and the hypophyseal portal system to regulate hormone secretion, and to the hindbrain and spinal cord to regulate feeding and autonomic tone^6,7^. Mice and humans with MC4R and SIM1 haplo-insufficiency develop hyperphagia, obesity and increased linear growth^8–14^.

In humans, candidate gene and exome sequencing studies have highlighted the prominent role of the leptin-melanocortin pathway in the regulation of energy homeostasis^15,16^. Moreover, weight loss drugs targeting the melanocortin pathway have recently been licensed for the management of severe obesity caused by genetic disorders affecting this pathway^17–19^. However, a substantial proportion of the heritability of obesity remains unexplained^20^. It is well-recognized that a large portion of the noncoding genome is functional and harbours genetic variants that may contribute to Mendelian disorders or influence complex traits^21^. Nonetheless, the interpretation of noncoding genetic variants in humans presents challenges, including the identification of disease-relevant cell types and the dissection of underlying mechanisms in the appropriate in vivo models.

MicroRNAs (miRNAs) are small noncoding RNAs that repress gene expression at the post-transcriptional level. Mammals express hundreds of distinct miRNAs, each with hundreds of target transcripts^22^. By regulating the expression of networks of genes, miRNAs modulate the function of multiple cellular processes simultaneously. MiR-7 is one of the most evolutionarily conserved miRNAs, with a mature miRNA sequence that is conserved from invertebrates to humans^23^. MiR-7 is highly expressed in the brain, pituitary, and pancreatic β-cells^24–26^. We have previously shown that miR-7 regulates gonadotropin and prolactin production in the pituitary^27,28^ and insulin secretion in pancreatic β-cells^29^. In the brain, miR-7 promotes neuronal differentiation and survival^30,31^. It is the most highly enriched miRNA in the hypothalamus^32^ and displays a distinct expression pattern in the ARC and PVN^33^, suggesting that it may be involved in the control of body weight by melanocortin circuits.

In this study, we carried out an in vivo genetic screen to systematically examine whether hypothalamic miR-7 regulates energy homeostasis. Mice with a global knockout of all three members of the miR-7 family (miR-7a1, miR-7a2, and miR-7b) exhibited obesity on a standard chow diet, which was exacerbated on a high-fat diet (HFD), demonstrating that miR-7 is essential for the control of energy balance. To identify the specific cell types mediating these effects, we deleted the miR-7 family in neurons expressing the *Leptin receptor* (*Lepr*), *Pomc*, *Agrp* and *Sim1*. We found that *Sim1-cre;mir-7*^*fl/fl*^ mice exhibited severe obesity due to increased food intake and decreased energy expenditure, associated with increased linear growth and increased insulin secretion. These phenotypes recapitulate SIM1 haploinsufficiency in mice and humans, and are likely explained by reduced expression of MC4R. In addition, *Sim1-cre;mir-7*^*fl/fl*^ mice exhibited reduced corticosterone levels and increased fluid intake due to impaired CRH and AVP function. We further identified *Snca* (α-Synuclein) and *Igsf8* (Immunoglobulin Superfamily Member 8) as miR-7 target genes whose overexpression in Sim1 neurons induces weight gain and endocrine abnormalities. In humans, *MIR7-1* is located in the last intron of *HNRNPK*, whose promoter drives the expression of both genes. By interrogating genome-wide association studies, we found that variants at the *HNRNPK* locus that reduce its expression are associated with increased height and truncal fat mass. Together, these studies demonstrate that miR-7 suppresses gene networks involved in the hypothalamic melanocortin pathway to regulate mammalian energy homeostasis.

## Results

### Genetic disruption of *mir-7* leads to severe obesity in mice

**Fig. 1:**
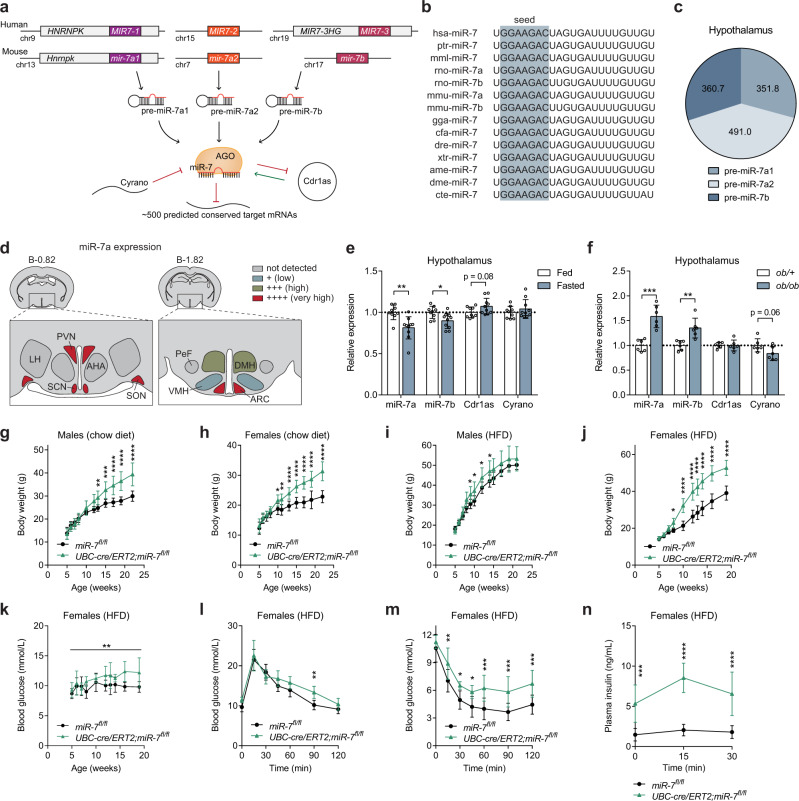
Severe obesity in mice lacking miR-7. **a** miR-7 genomic loci and organisation of the neuronal regulatory network comprising Cyrano, Cdr1as, and miR-7. Primary miRNA transcripts are processed into precursor miRNA (pre-miR), and then into mature miRNA (miR), which is loaded onto an AGO protein to interact with miRNA binding sites. **b** miR-7 sequences in different species. The grey box indicates the seed sequence. hsa, *Homo sapiens*; ptr, *Pan troglodytes*; mml, *Macaca mulatta*; rno, *Rattus norvegicus*; mmu, *Mus musculus*; gga, *Gallus gallus*; cfa, *Canis familiaris*; dre, *Danio rerio*; xtr, *Xenopus tropicalis*; ame, *Apis mellifera*; dme, *Drosophila melanogaster*; cte, *Capitella teleta*. **c** Contribution of each miR-7 precursor to the absolute expression of miR-7 in the hypothalamus. Values indicate copies/pg RNA. **d** miR-7 expression in hypothalamic nuclei, based on published data^33^. AHA anterior hypothalamic area, ARC arcuate nucleus, DMH dorsomedial hypothalamus, LH lateral hypothalamus, PeF perifornical nucleus, PVN paraventricular nucleus, SCN suprachiasmatic nucleus, SON supraoptic nucleus, VMH ventromedial hypothalamus. Brain coordinates indicate distance from Bregma (“B”) in mm. **e–f** Hypothalamic gene expression in fed and fasted (relative to fed) (*n* = 9 and 10 animals) (**e**) or *ob/+* and *ob/ob* mice (relative to *ob/+*) (*n* = 6 animals) (**f**). **(g–j)** Body weight of chow-fed male (*n* = 8 and 10 animals) (**g**), chow-fed female (*n* = 12 and 8 animals) (**h**), HFD-fed male (*n* = 12 and 8 animals) (**i**), and HFD-fed female (*n* = 10 animals) (**j**) *mir-7*^*fl/fl*^ and *UBC-cre/ERT2;mir-7*^*fl/fl*^ *mice*. **k–n** Random fed blood glucose (*n* = 10 animals) (k), blood glucose during an ipGTT (*n* = 8 animals) (**l**), blood glucose during an ipITT (*n* = 10 animals) (**m**), and plasma insulin during an ipGTT (*n* = 7 animals) (**n**) in HFD-fed female *mir-7*^*fl/fl*^ and *UBC-cre/ERT2; mir-7*^*fl/fl*^ mice. Data are presented as mean ± SD, where error bars are present. *P < 0.05; **P < 0.01; ***P < 0.001; ****P < 0.0001; no asterisk indicates *P* > 0.05; two-tailed *t* test (**e, f**) or 2-way repeated measures ANOVA with Sidak’s multiple comparisons test (**g**, **h**, **i**, **j**, **l**, **m**, **n**) or 2-way repeated measures ANOVA (**k**). Source data are provided as a Source Data file.

In mice and humans, the miR-7 family comprises three precursors (miR-7a1/7-1, miR-7a2/7-2, and miR-7b/7-3), which are encoded by different genomic loci but possess identical seed sequences with which they regulate the same repertoire of targets^34^ (Fig. 1a, b). We found that the hypothalamus expresses miR-7a1, miR-7a2, and miR-7b at approximately equal levels (Fig. 1c). Specifically, miR-7 is highly enriched in the ARC and PVN of the hypothalamus (Fig. 1d). Hypothalamic miR-7 was regulated by the metabolic state, with reduced expression during fasting (Fig. 1e) and increased expression in obese *ob/ob* mice (Fig. 1f). In the brain, miR-7 is implicated in a unique regulatory noncoding RNA network, in which the long noncoding RNA, Cyrano, degrades miR-7 and prevents miR-7-mediated destabilisation of the circular RNA, Cdr1as^35^. Cdr1as acts reciprocally by sponging miR-7 through its >70 binding sites, a process, which protects miR-7 from degradation and is thought to regulate its transport within neurons^36^. We found that Cdr1as and Cyrano showed trends to be inversely correlated with miR-7 expression in the hypothalamus (Fig. 1e, f), consistent with the previously described relationships in this regulatory network.

To investigate the physiological role of miR-7, we generated mice with loxP sites flanking the *mir-7a1*, *mir-7a2*, and *mir-7b* alleles (hereafter referred to as *mir-7*^*fl/fl*^) and crossed these with mice harbouring a *UBC-cre/ERT2* allele to introduce a global, tamoxifen-inducible knockout of all three members of the miR-7 family (Supplementary Fig. 1a). Both male and female *UBC-cre/ERT2; mir-7*^*fl/fl*^ mice exhibited obesity on chow diet (Fig. 1g, h) and high-fat diet (HFD) (Fig. 1i, j), demonstrating that the miR-7 family plays an important role in the regulation of body weight. We also measured random fed blood glucose concentrations over time and performed intraperitoneal glucose tolerance tests (ipGTT) and intraperitoneal insulin tolerance tests (ipITT). Blood glucose levels were unchanged or slightly elevated in chow-fed *UBC-cre/ERT2;mir-7*^*fl/fl*^ mice, whereas these mice exhibited improved glucose tolerance in association with increased insulin secretion and normal insulin sensitivity (Supplementary Fig. 1b–h). In HFD-fed *UBC-cre/ERT2;mir-7*^*fl/fl*^ mice, blood glucose levels were unchanged in males (Supplementary Fig. 1i) and increased in females (Fig. 1k). Glucose tolerance was unchanged or only slightly impaired (Fig. 1l, Supplementary Fig. 1j) in spite of severe insulin resistance (Fig. 1m, Supplementary Fig. 1k), due to increased insulin secretion (Fig. 1n).

### Deletion of *mir-7* in Sim1 neurons drives hyperphagia, weight gain and increased insulin secretion

**Fig. 2:**
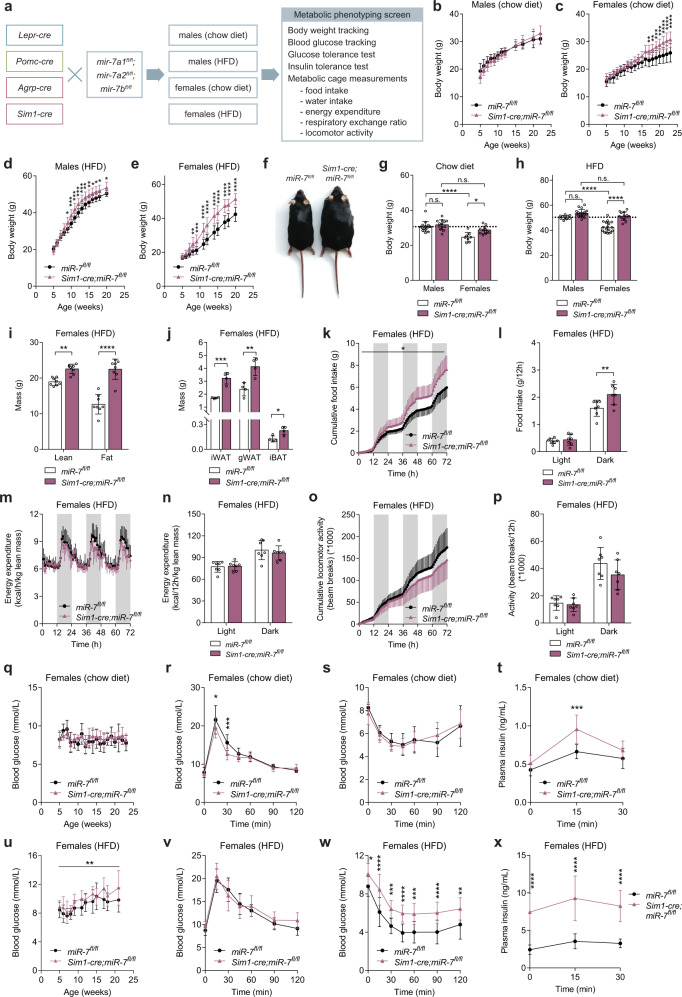
Obesity, hyperphagia, and increased insulin secretion upon deletion of *mir-7* in Sim1 neurons. **a** Experimental strategy to assess the functions of miR-7 in hypothalamic neuron populations. **b–e** Body weight of chow-fed male (*n* = 15 and 13 animals) (**b**), chow-fed female (*n* = 8 and 11 animals) (**c**), HFD-fed male (n = 10 and 19 animals) (d), and HFD-fed female (*n* = 18 and 13 animals) (**e**) *mir-7*^*fl/fl*^ and *Sim1-cre;mir-7*^*fl/fl*^ mice. **f** Representative photograph of HFD-fed female *mir-7*^*fl/fl*^ and *Sim1-cre;mir-7*^*fl/fl*^ mice. **g–h** Male and female body weight in 20 week-old chow-fed (**g**) and HFD-fed (**h**) *mir-7*^*fl/fl*^ and *Sim1-cre;mir-7*^*fl/fl*^ mice, using data shown in Fig. 2b–e. Dashed line indicates the body weight of *mir-7*^*fl/fl*^ males. **i–j** Lean and fat mass (*n* = 8 animals) (**i**) and adipose depot mass (*n* = 4 animals) (**j**) of HFD-fed female *mir-7*^*fl/fl*^ and *Sim1-cre;mir-7*^*fl/fl*^ mice. **k–p** 72-hour metabolic cage measurements of HFD-fed female *mir-7*^*fl/fl*^ and *Sim1-cre;mir-7*^*fl/fl*^ mice (*n* = 7 animals). (**k)** Cumulative food intake; (**l**) average food intake per 12-h phase; (**m**) hourly energy expenditure; (**n**) average energy expenditure per 12-hour phase; (**o**) cumulative locomotor activity; (**p**) average locomotor activity per 12-hour phase. Dark phases are shaded in grey. **q–t** Random fed blood glucose (*n* = 8 and 11 animals) (**q**), blood glucose during an ipGTT (*n* = 8 and 11 animals) (**r**), blood glucose during an ipITT (*n* = 6 and 9 animals) (**s**), and plasma insulin during an ipGTT (*n* = 6 animals) (**t**) in chow-fed female *mir-7*^*fl/fl*^ and *Sim1-cre;mir-7*^*fl/fl*^ mice. **u–x** Random fed blood glucose (*n* = 18 and 13 animals) (**u**), blood glucose during an ipGTT (*n* = 16 and 12 animals) (**v**), blood glucose during an ipITT (*n* = 16 and 12 animals) (**w**), and plasma insulin during an ipGTT (*n* = 7 animals) (**x**) in HFD-fed female *mir-7*^*fl/fl*^ and *Sim1-cre;mir-7*^*fl/fl*^ mice. Data are presented as mean ± SD. *P < 0.05; **P < 0.01; ***P < 0.001, ****P < 0.0001; no asterisk indicates *P* > 0.05; 2-way repeated measures ANOVA with Sidak’s multiple comparisons test (**b**, **c**, **d**, **e**, **g**, **h**, **l**, **n**, **p**, **r**, **s**, **t**, **v**, **w**, **x**), two-tailed *t* test **i, j**, or 2-way repeated measures ANOVA (**k**, **m**, **o**, **q**, **u**). Source data are provided as a Source Data file.

To identify the specific hypothalamic cell populations involved in the miR-7-mediated control of energy homeostasis, we generated conditional knockout mouse models by crossing *mir-7*^*fl/fl*^ mice with mice harbouring a *Lepr-cre*, *Pomc-cre*, *Agrp-cre*, or *Sim1-cre* allele (Fig. 2a) and performed a comprehensive metabolic phenotyping screen in males and females fed a chow diet or HFD. The most prominent obesity phenotype occurred upon the Sim1 neuron-specific loss of miR-7 (Fig. 2b–f). When fed a regular chow diet, body weight was increased in *Sim1-cre;mir-7*^*fl/fl*^ females, but not males (Fig. 2b, c). When challenged with chronic HFD feeding, obesity was significantly exacerbated in *Sim1-cre;mir-7*^*fl/fl*^ mice of both sexes (Fig. 2d, e). We verified that the *Sim1-cre* transgene itself did not affect the body weight of HFD-fed mice (Supplementary Fig. 2a, b). Comparison of the body weight between male and female animals revealed that miR-7 in Sim1 neurons is required to maintain the sexual dimorphism of body weight in mice. In both chow- and HFD-fed mice, *mir-7*^*fl/fl*^ females had a lower body weight than *mir-7*^*fl/fl*^ males, whereas *Sim1-cre;mir-7*^*fl/fl*^ females displayed an increase in body weight that matched the body weight of male mice (Fig. 2g, h). HFD-fed *Sim1-cre;mir-7*^*fl/fl*^ females exhibited an increase in fat mass and lean mass (Fig. 2i), as well as increased mass of adipose tissue depots (Fig. 2j). In chow-fed *Sim1-cre;mir-7*^*fl/fl*^ females studied in metabolic cages, food intake was unchanged (Supplementary Fig. 2c, d) but energy expenditure was reduced (Supplementary Fig. 2e, f), without any changes in respiratory exchange ratio (RER) or locomotor activity (Supplementary Fig. 2g–j). In metabolic cage experiments performed one week after the onset of HFD feeding, *Sim1-cre;mir-7*^*fl/fl*^ females exhibited increased food intake (Fig. 2k, l) and RER (Supplementary Fig. 2k, l) compared to *mir-7*^*fl/fl*^ controls, while energy expenditure and locomotor activity were unaffected (Fig. 2m–p). No changes in metabolic measurements were detected in males (Supplementary Fig. 2m–t). The expression of *Pgc1a* (Peroxisome proliferator-activated receptor gamma co-activator-1) in interscapular brown adipose tissue (iBAT) was significantly reduced and expression of *Ucp1* (Uncoupling protein 1) nominally reduced (*p* = 0.06) in *Sim1-cre;mir-7*^*fl/fl*^ mice, compared to *mir-7*^*fl/fl*^ control mice (Supplementary Fig. 2u). We also detected upregulation of *Adrb3* (encoding the β-3 adrenergic receptor), which has previously been observed as a compensatory response to a blockade of sympathetic input^37,38^. These results suggest that reduced sympathetic activation of iBAT contributes to the lower energy expenditure in *Sim1-cre;mir-7*^*fl/fl*^ mice.

On chow diet, *Sim1-cre;mir-7*^*fl/fl*^ females displayed no change in random fed blood glucose levels compared to *mir-7*^*fl/fl*^ controls (Fig. 2q). However, glucose tolerance was improved at 8–9 weeks of age (Fig. 2r), prior to any changes in body weight (Fig. 2c). This improvement in glucose clearance was not due to changes in insulin sensitivity (Fig. 2s) but correlated with increased glucose-stimulated insulin secretion (Fig. 2t). In HFD-fed *Sim1-cre; mir-7*^*fl/fl*^ females, random fed blood glucose levels were consistently elevated compared to *mir-7*^*fl/fl*^ controls (Fig. 2u). However, at 8–9 weeks of age, glucose tolerance was unchanged (Fig. 2v). This maintenance of normal glucose tolerance, in spite of obesity (Fig. 2e) and insulin resistance (Fig. 2w), was correlated with marked hyperinsulinemia in *Sim1-cre;mir-7*^*fl/fl*^ mice (Fig. 2x). Measurements of glucose homeostasis in male *Sim1-cre;mir-7*^*fl/fl*^ mice revealed only non-significant trends (Supplementary Fig. 2v–aa). To verify that the hyperinsulinemic phenotype was not caused by aberrant Cre recombinase activity and *mir-7* deletion in β-cells^29^, we performed immunofluorescence analysis of the *LSL-tdTomato* reporter allele in the pancreas of *Sim1-cre;LSL-tdTomato* mice and observed no recombination (Supplementary Fig. 2ab). Furthermore, miR-7 expression was unchanged in the pancreatic islets of *Sim1-cre;mir-7*^*fl/fl*^ mice versus *mir-7*^*fl/fl*^ controls (Supplementary Fig. 2ac). Thus, miR-7 acts cooperatively in pancreatic β-cells^29^ and Sim1 neurons to regulate insulin secretion. Sim1- and MC4R-expressing neurons control insulin secretion by regulating autonomic tone^39,40^, and deficiency of Sim1 or MC4R results in hyperinsulinemia in mice and humans^41–43^.

### Deletion of *mir-7* in Sim1 neurons causes neuroendocrine abnormalities

**Fig. 3:**
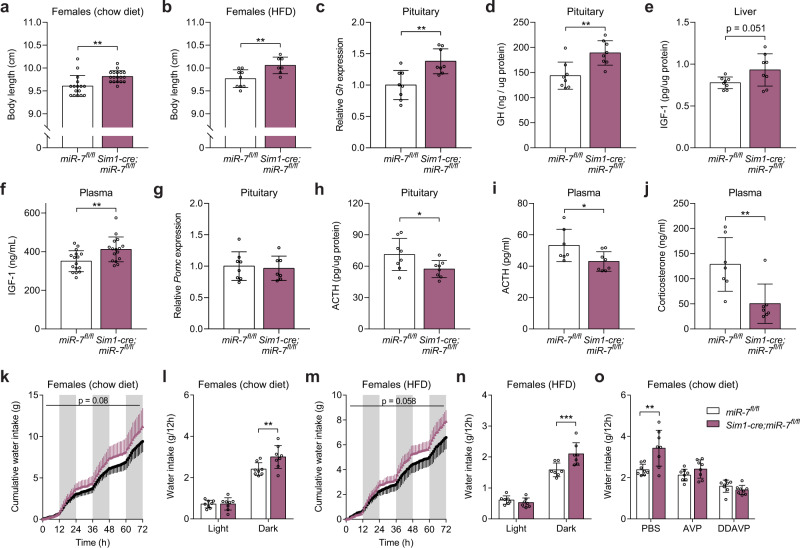
Neuroendocrine abnormalities in mice lacking miR-7 in Sim1 neurons. **a–b** Body length of chow-fed female (*n* = 16 and 18 animals) (**a**) and HFD-fed female (*n* = 9 and 7 animals) (**b**) *mir-7*^*fl/fl*^ and *Sim1-cre;mir-7*^*fl/fl*^ mice. **c–j** Pituitary *Gh* expression (relative to *mir-7*^*fl/fl*^) (*n* = 8 animals) (**c**), pituitary GH content (*n* = 8 animals) (**d**), liver IGF-1 content (*n* = 8 animals) (**e**), plasma IGF-1 concentration (*n* = 16 animals) (**f**), pituitary *Pomc* expression (relative to *mir-7*^*fl/fl*^) (*n* = 8 and 7 animals) (**g**), pituitary ACTH content (*n* = 8 animals) (**h**), plasma ACTH concentration (*n* = 8 and 7 animals) (**i**), and plasma corticosterone concentration (*n* = 7 animals) (**j**), in chow-fed *mir-7*^*fl/fl*^ and *Sim1-cre; mir-7*^*fl/fl*^ mice. **k**–**n** 72-hour metabolic cage measurements of chow-fed (*n* = 8 animals) (**k**–**l**) and HFD-fed (*n* = 7 animals) (**m-n**) female *mir-7*^*fl/fl*^ and *Sim1-cre;mir-7*^*fl/fl*^ mice. **k, m** cumulative water intake; (**l, n**) average water intake per 12-hour phase. Dark phases are shaded in grey. **o** Water intake of *mir-7*^*fl/fl*^ and *Sim1-cre;mir-7*^*fl/fl*^ mice during a 12-hour dark phase following intraperitoneal injection of PBS (phosphate-buffered saline), AVP ((Arg8)-Vasopressin), or DDAVP (Desmopressin) (*n* = 8 animals). Data are presented as mean ± SD. *P < 0.05; **P < 0.01; ***P < 0.001; no asterisk indicates *P* > 0.05; two-tailed *t* test (**a–j**), 2-way repeated measures ANOVA (**k, m**), or 2-way repeated measures ANOVA with Sidak’s multiple comparisons test (**l, n, o**). Source data are provided as a Source Data file.

Subsets of Sim1 neurons release neuropeptides from axon terminals in the posterior pituitary and hypophyseal portal system to control endocrine axes involved in growth, stress response, and fluid homeostasis. We found that the loss of miR-7 increased linear growth in chow-fed females but not males (Fig. 3a, Supplementary Fig. 3a), and in both HFD-fed males and females (Fig. 3b, Supplementary Fig. 3b). Despite their obesity, we found increased levels of growth hormone (GH) mRNA and protein in the pituitary, as well as increased insulin-like growth factor 1 (IGF-1) protein levels in the liver and plasma of *Sim1-cre;mir-7*^*fl/fl*^ mice compared to *mir-7*^*fl/fl*^ controls (Fig. 3c–f). These findings align with results in human MC4R deficiency, where the pulsatile secretion of GH is retained compared to weight-matched controls^42^. Our findings in *Sim1-cre;mir-7*^*fl/fl*^ mice indicate that increased linear growth may be a consequence of both reduced somatostatin (increasing GH secretion) and increased insulin secretion, as also seen in mice and humans with MC4R deficiency^39,42^.

In *Sim1-cre;mir-7*^*fl/fl*^ mice, pituitary *Pomc* expression was unchanged (Fig. 3g), but pituitary adrenocorticotropic hormone (ACTH) content, plasma ACTH levels, and plasma corticosterone levels were reduced (Fig. 3h–j), indicating a suppression of the hypothalamic-pituitary-adrenal (HPA) stress-response axis downstream of CRH-expressing Sim1 neurons.

In metabolic cages, *Sim1-cre;mir-7*^*fl/fl*^ mice exhibited increased water intake compared to *mir-7*^*fl/fl*^ controls (Fig. 3k–n, Supplementary Fig. 3c, d). We found that this phenotype could be reversed by administration of AVP or Desmopressin (DDAVP), a synthetic vasopressin analog that acts on the V2 receptor to mediate the antidiuretic effect of AVP in renal tubular cells (Fig. 3o). These results suggest that inadequate levels of AVP released from Sim1 neurons caused impaired water retention and excess water intake in *Sim1-cre;mir-7*^*fl/fl*^ mice.

### Deletion of *mir-7* in POMC- and LepR-expressing neurons causes weight gain

We also performed the metabolic phenotyping screen (Fig. 2a) in the other conditional knockout mouse models. In *Pomc-cre;mir-7*^*fl/fl*^ mice, we observed no change in body weight on chow diet (Supplementary Fig. 4a, b), but detected a slight exacerbation of HFD-induced obesity in females, with a similar trend toward weight gain in males (Supplementary Fig. 4c, d). In metabolic cage experiments, we measured no differences in food intake, water intake, energy expenditure, RER, or locomotor activity of chow-fed males or females (Supplementary Fig. 4e–x). Finally, we found slightly elevated blood glucose levels and impaired glucose tolerance in *Pomc-cre;mir-7*^*fl/fl*^ females, but not males (Supplementary Fig. 4y–ah). Loss of miR-7 in LepR-expressing neurons yielded a similar phenotype as the POMC neuron-specific ablation of *mir-7* (Supplementary Fig. 5a–ah). In *Agrp-cre;mir-7*^*fl/fl*^ mice, we observed no changes in body weight or glucose homeostasis in chow or HFD-fed mice of either sex (Supplementary Fig. 6a–ah). These data indicate that loss of miR-7 in POMC and LepR neurons leads to a mild exacerbation of diet-induced obesity and impaired glucose tolerance, whereas miR-7 does not appear to modulate any metabolic functions of AgRP neurons.

### Reduced neuron abundance and neuropeptide expression in Sim1 neurons lacking miR-7

**Fig. 4:**
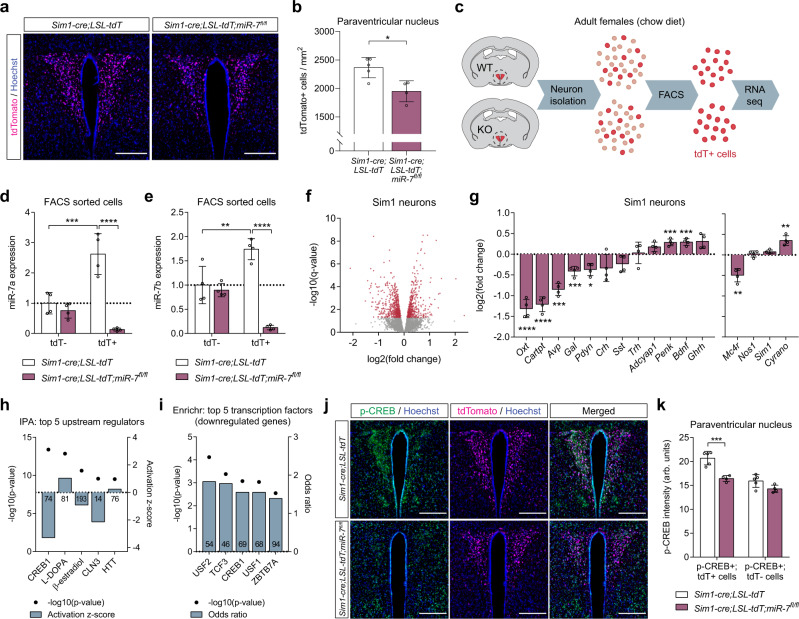
Cellular dysfunction of Sim1 neurons upon *mir-7* ablation. **a–b** Representative image (scale bar: 250 μm) (**a**) and quantification (*n* = 5 and 4 animals) (**b**) of tdTomato-positive cells within the PVN of *Sim1-cre;LSL-tdTomato* and *Sim1-cre;LSL-tdTomato;mir-7*^*fl/fl*^ mice. Magenta, tdTomato; blue, Hoechst. **c** Schematic of sample preparation for RNA sequencing of Sim1 neurons from *Sim1-cre;LSL-tdTomato* (“WT”) and *Sim1-cre;LSL-tdTomato;mir-7*^*fl/fl*^ (“KO”) mice. **d–e** miR-7a (**d**) and miR-7b (**e**) expression in tdTomato-negative and tdTomato-positive FACS-sorted cells from hypothalamus of *Sim1-cre;LSL-tdTomato* and *Sim1-cre;LSL-tdTomato;mir-7*^*fl/fl*^ mice (*n* = 4 samples, with 4 mice pooled per sample). Expression is relative to tdT-negative cells from *Sim1-cre;LSL-tdTomato* mice. **f** –log10(*q*-value) and log2(fold change) of all expressed genes in *Sim1-cre;LSL-tdTomato;mir-7*^*fl/fl*^ versus *Sim1-cre;LSL-tdTomato* RNA sequencing data. Significantly regulated genes (*q* < 0.05) are indicated in red. **g** log2(fold change) of key neuropeptide-encoding genes (left panel), and *Mc4r*, *Nos1*, *Sim1*, and *Cyrano* (right panel) in *Sim1-cre;LSL-tdTomato;mir-7*^*fl/fl*^ versus *Sim1-cre;LSL-tdTomato* RNA sequencing data (*n* = 4 samples, with 4 mice pooled per sample). **h** –log10(p-value) and activation z-scores of top upstream regulators identified by QIAGEN Ingenuity Pathway Analysis of *Sim1-cre;LSL-tdTomato;mir-7*^*fl/fl*^ versus *Sim1-cre;LSL-tdTomato* RNA sequencing data. Numbers in the bars indicate the number of genes contributing to the prediction. **i** –log10(p-value) and odds ratio of top transcription factors identified by Enrichr “ENCODE and ChEA Consensus Transcription Factors from ChIP-X” of downregulated genes in *Sim1-cre;LSL-tdTomato;mir-7*^*fl/fl*^ versus *Sim1-cre;LSL-tdTomato* RNA sequencing data. Numbers in the bars indicate the number of genes contributing to the prediction. **j–k** Representative immunofluorescence (scale bar: 250 μm) (**j**) and quantification (*n* = 5 and 4 animals) (**k**) of p-CREB intensity in p-CREB+ /tdTomato+ cells and p-CREB+ /tdTomato– cells in the PVN of *Sim1-cre;LSL-tdTomato* and *Sim1-cre;LSL-tdTomato;mir-7*^*fl/fl*^ mice. Green, p-CREB; magenta, tdTomato; blue, Hoechst. Data are presented as mean ± SD, where error bars are present. *P < 0.05; **P < 0.01; ***P < 0.001; ****P < 0.0001; no asterisk indicates P > 0.05; two-tailed *t* test (**b**), 2-way ANOVA with Sidak’s multiple comparisons test (**d, e, k**), or Fisher’s exact test (**h, i**). *q < 0.05; **q < 0.01; ***q < 0.001; ****q < 0.0001; quasi-likelihood (QL) differential expression test (**f, g**). Source data are provided as a Source Data file.

Sim1 haploinsufficiency in mice is associated with a 24% reduction in the number of PVN neurons^8^. We examined the PVN in mice expressing tdTomato in Sim1 neurons and observed an 18% loss of Sim1 neurons in *Sim1-cre; LSL-tdTomato; mir-7*^*fl/fl*^ mice compared to *Sim1-cre;LSL-tdTomato* control mice (Fig. 4a, b). Whilst neuronal loss may contribute to the metabolic phenotype, it is notable that postnatal deletion of Sim1 induces a similar phenotype without affecting cell number^10,44^. We next performed RNA sequencing of FACS-sorted Sim1 neurons from *Sim1-cre;LSL-tdTomato* and *Sim1-cre;LSL-tdTomato;mir-7*^*fl/fl*^ mice (Fig. 4c). In control mice, expression of miR-7a and miR-7b was enriched in tdTomato+ neurons compared to tdTomato- neurons of the hypothalamus (Fig. 4d, e). Furthermore, miR-7 expression was only reduced in tdTomato+, but not tdTomato- neurons of *Sim1-cre;LSL-tdTomato;mir-7*^*fl/fl*^ mice compared to controls (Fig. 4d, e).

RNA sequencing revealed 522 upregulated transcripts and 651 downregulated transcripts in Sim1 neurons lacking miR-7 (Fig. 4f). In particular, we observed reduced expression of *Mc4r* and several key neuropeptides, including *Oxt*, *Avp*, and Prodynorphin (*Pdyn*) (Fig. 4g). An unbiased QIAGEN Ingenuity Pathway Analysis (IPA) of RNA sequencing data identified CREB1 as the top predicted regulator of gene expression changes in *Sim1-cre;LSL-tdTomato;mir-7*^*fl/fl*^ versus *Sim1-cre;LSL-tdTomato* mice, with a negative z-score indicating inhibited function (Fig. 4h). In addition, CREB1 was among the top 5 Enrichr results from “ENCODE and ChEA Consensus Transcription Factors from ChIP-X” for significantly downregulated genes (Fig. 4i). Immunofluorescence analysis of phosphorylated CREB (p-CREB) in Sim1 neurons revealed reduced intensity of p-CREB signal in double-positive p-CREB+/tdTomato+ cells, but not in p-CREB+/tdTomato- cells in *Sim1-cre;LSL-tdTomato;mir-7*^*fl/fl*^ mice compared to control mice (Fig. 4j, k). CREB mediates the effects of canonical MC4R signalling through the G_αs_–cAMP–PKA cascade^45–47^, and mice with genetic deletion of *Creb1* in Sim1 neurons develop obesity, impaired thermogenesis, and reduced AVP expression^48^. Therefore, reduced CREB activation likely contributes to the metabolic defects in mice lacking miR-7 in Sim1 neurons.

No change was found in the expression of *Nos1*, a marker of the Sim1 neuron subpopulation that contains the OXT-expressing neurons^49^. This suggests that the downregulation of specific neuropeptides such as *Oxt* represents a gene expression defect, rather than a subtype-specific loss of cell populations (Fig. 4g). Immunofluorescence analysis of OXT in Sim1 neurons confirmed that subtype-specific cell loss was not responsible for the downregulation of *Oxt* expression, as the number of OXT+ neurons was reduced to a similar extent as that of Sim1 neurons (Supplementary Fig. 7a, b). PDYN and MC4R are produced in distinct subsets of Sim1 neurons that act independently to reduce feeding^6^; additionally, both AVP- and OXT-expressing neurons have been implicated in Sim1 neuron-induced satiety^9,50^. Thus, dysfunction of several Sim1 neuron subpopulations likely contributes to the hyperphagic obesity of *Sim1-cre;mir-7*^*fl/fl*^ mice.

In the brain, miR-7 forms a unique regulatory network with Cdr1as and Cyrano. While Cyrano has been previously shown to induce degradation of miR-7 in the hypothalamus, a reciprocal effect of miR-7 on hypothalamic Cyrano expression was not observed^35^. Interestingly, Cyrano was 1.3-fold upregulated in Sim1 neurons lacking miR-7 (Fig. 4g). As we have found that miR-7 is enriched in Sim1 neurons compared to the rest of the hypothalamus (Fig. 4d, e), it is plausible that its particularly high expression in Sim1 neurons enables miR-7 to regulate Cyrano in a cell type-specific manner that is not detectable at the whole-tissue level.

### The miR-7 target genes *Snca* and *Igsf8* act in Sim1 neurons to regulate body weight

**Fig. 5:**
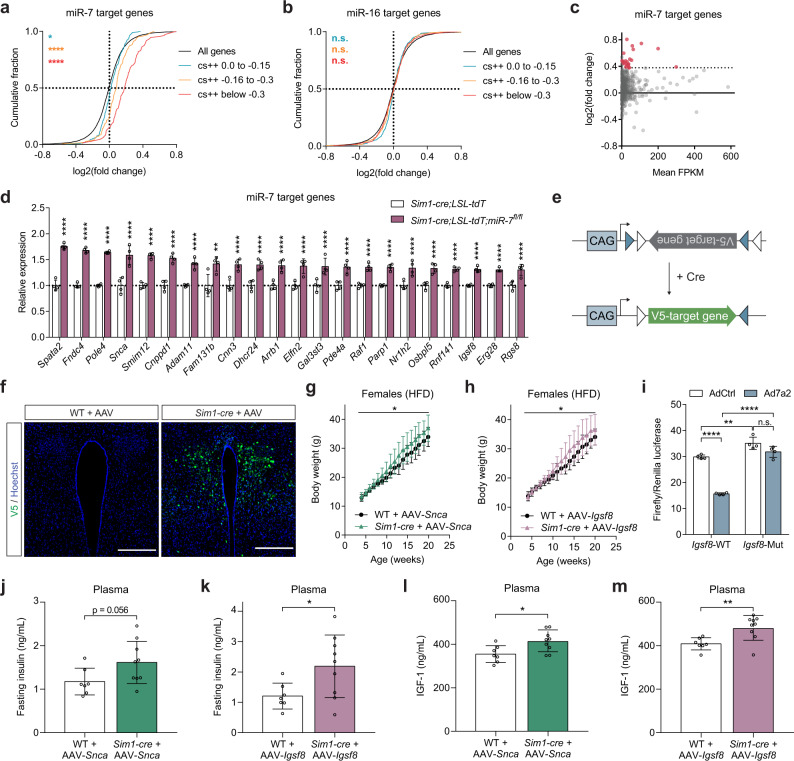
Identification of miR-7 target genes involved in Sim1 neuron function. **a–b** Cumulative frequency distribution of mRNA changes for predicted targets of miR-7 (**a**) or miR-16 (**b**) in *Sim1-cre;LSL-tdTomato;mir-7*^*fl/fl*^ versus *Sim1-cre;LSL-tdTomato* RNA sequencing data. Targets are binned by cumulative weighted context ++  score (cs + +) and compared to all expressed genes. Number of genes per bin for miR-7: black, 16830; blue, 225; orange, 149; red, 112. Number of genes per bin for miR-16: black, 16830; blue, 332; orange, 408; red, 425. **c** log2(fold change) and mean FPKM (fragments per kilobase of transcript per million fragments mapped) of all predicted conserved miR-7 target genes in *Sim1-cre;LSL-tdTomato;mir-7*^*fl/fl*^ versus *Sim1-cre;LSL-tdTomato* RNA sequencing data. Targets with >1.3-fold upregulation are shown in red. **d** Expression (relative to *Sim1-cre;LSL-tdTomato*) of upregulated targets in *Sim1-cre;LSL-tdTomato;mir-7*^*fl/fl*^ versus *Sim1-cre;LSL-tdTomato* RNA sequencing data (*n* = 4 samples, with 4 mice pooled per sample). **e** Strategy of Cre-dependent target overexpression using a pAAV-FLEx vector. **f** V5 immunofluorescence in the PVN of WT (representative of 3 mice) and *Sim1-cre* mice (representative of 3 mice), 4 months after injection with AAV-*Snca* (scale bar: 250 μm). Green, V5; Blue, Hoechst. **g–h** Body weight of HFD-fed female WT and *Sim1-cre* mice injected with AAV-*Snca* (*n* = 7 and 9 animals) (**g**) or AAV-*Igsf8* (*n* = 7 and 9 animals) (**h**). **i** Luciferase assay of cells expressing the WT or mutated 3’UTR of *Igsf8*, with or without miR-7 overexpression (*n* = 4; representative of two independent experiments). **j–m** Plasma insulin (**j–k**) and IGF-1 (**l–m**) in WT and *Sim1-cre* mice injected with AAV-*Snca* (*n* = 7 and 9 animals) (**j, l**) or AAV-*Igsf8* (*n* = 7 and 9 animals) (**k, m**). Data are presented as mean ± SD, where error bars are present. *P < 0.05; **P < 0.01; ***P < 0.001; ****P < 0.0001; no asterisk or n.s. indicates *P* > 0.05; Kruskal-Wallis test with Dunn’s multiple comparisons test (**a, b**), 2-way repeated measures ANOVA (**g, h**; genotype-by-time interaction), 2-way ANOVA with Sidak’s multiple comparisons test (i), or two-tailed *t* test (**j–m**). **q < 0.01; ****q < 0.0001; quasi-likelihood (QL) differential expression test (**c, d**). Source data are provided as a Source Data file.

MiRNAs directly repress target transcripts possessing a specific miRNA binding site in their 3’UTR. We found that mRNAs with a conserved 8mer, 7mer-m8, or 7mer-A1 binding site for miR-7 in their 3’UTR (classified by TargetScanMouse release 7.2^51^) were preferentially upregulated in Sim1 neurons of *Sim1-cre;LSL-tdTomato;mir-7*^*fl/fl*^ mice compared to *Sim1-cre;LSL-tdTomato* controls (Fig. 5a). In comparison, predicted targets of miR-16, which is ubiquitously expressed and is abundant in the mouse hypothalamus^52^, were not enriched (Fig. 5b). After applying a threshold of 1.3-fold upregulation, RNA sequencing revealed 22 predicted target genes of miR-7 (Fig. 5c, d). In mammals, miRNAs predominantly regulate their target protein levels through destabilization and degradation of their target mRNA; as such, regulation of target mRNA expression and protein translation is tightly correlated^53^. By manipulating miR-7 expression in neuronal cells, we confirmed that miR-7 regulates its target mRNA and protein levels in a consistent manner (Supplementary Fig. 8a–d).

To investigate the role of the identified miR-7 target genes in Sim1 neuron function, we performed AAV-mediated, Cre-dependent overexpression of selected targets in Sim1 neurons using a pAAV-FLEx vector in an AAV-PHP.eB capsid that efficiently transduces the CNS upon intravenous administration^54,55^ (Fig. 5e). For this analysis, we selected seven of the 22 target genes that were upregulated upon *mir-7* ablation: the five most upregulated genes (*Spata2*, *Fndc4*, *Pole4*, *Snca*, and *Smim12*), as well as *Arrb1* (the gene encoding Beta-arrestin-1), due to its known role in MC4R signalling^56–58^, and *Igsf8*, which had the highest baseline expression among the upregulated target genes and is known to modulate neuronal function^59,60^. We verified that these constructs induced expression that persisted in the PVN for at least 4 months after injection (Fig. 5f, Supplementary Fig. 8e, f). Comparing the body weight of AAV-injected *Sim1-cre* mice versus AAV-injected wild-type littermates, we found that overexpression of *Snca* (Fig. 5g) and *Igsf8* (Fig. 5h) caused an exacerbation of HFD-induced obesity, whereas overexpression of *Spata2*, *Pole4*, *Fndc4*, *Smim12*, or *Arrb1* had no impact on body weight (Supplementary Fig. 8g–k). Both *Snca* (encoding α-Synuclein) and *Igsf8* (encoding Immunoglobulin Superfamily Member 8) contain a conserved 8-mer binding site for miR-7, but only *Snca* has been previously confirmed as a direct miR-7 target gene^29,31^. We therefore cloned the 3’UTR of *Igsf8*, containing either the wild-type or mutated miR-7 binding sites, into a luciferase reporter construct and confirmed by luciferase assay that *Igsf8* is a direct target of miR-7 (Fig. 5i). In addition to their effects on body weight, overexpression of both *Snca* and *Igsf8* in Sim1 neurons led to an increase in plasma insulin (Fig. 5j, k) and plasma IGF-1 (Fig. 5l, m), whereas no differences were observed upon overexpression of *Spata2*, *Pole4*, *Fndc4*, *Smim12*, or *Arrb1* (Supplementary Fig. 8l–p). The fact that, among all investigated targets, only *Snca* and *Igsf8* have a detectable function in Sim1 neurons may be related to their enriched expression in these cells; *Snca* and *Igsf8* display the highest baseline expression out of the 22 upregulated targets in Sim1 neurons (Supplementary Fig. 8q). Corticosterone levels and water intake were unchanged upon overexpression of *Snca* or *Igsf8* (Supplementary Fig. 8r–u), suggesting that the function of CRH- and AVP-expressing Sim1 neurons was not impaired by these targets. Food intake was slightly increased in mice with *Igsf8* overexpression (Supplementary Fig. 8v, w), while energy expenditure was slightly reduced in mice with *Snca* overexpression (Supplementary Fig. 8x, y). Taken together, overexpression of *Snca* and *Igsf8* in Sim1 neurons each partly recapitulates the phenotype of *Sim1-cre;mir-7*^*fl/fl*^ mice. These data identify *Snca* and *Igsf8* as direct targets of miR-7 that play a functional role in Sim1 neurons to regulate body weight and endocrine axes.

### Human variants in the locus encompassing *MIR7-1* are associated with height and adiposity

*MIR7-1/mir-7a1* is located in the last intron of a ubiquitously expressed gene, *HNRNPK* (Heterogeneous Nuclear Riboucleoprotein K), an RNA-binding protein that complexes with pre-mRNAs in the nucleus to influence their processing and transport. *MIR7-1* is co-transcribed with its host gene, and its tissue specificity is achieved by inhibition of miRNA maturation in non-neuronal cells^61–63^. To investigate the potential contribution of variation in miR-7 expression to human adiposity, height and metabolic traits, we interrogated Genome-Wide Association Studies (GWAS) and expression quantitative trait loci (eQTL) using publicly available resources which collate numerous large datasets (OpenTargets^64^, FIVEx^65^, PhenomeXcan^66^).

**Fig. 6:**
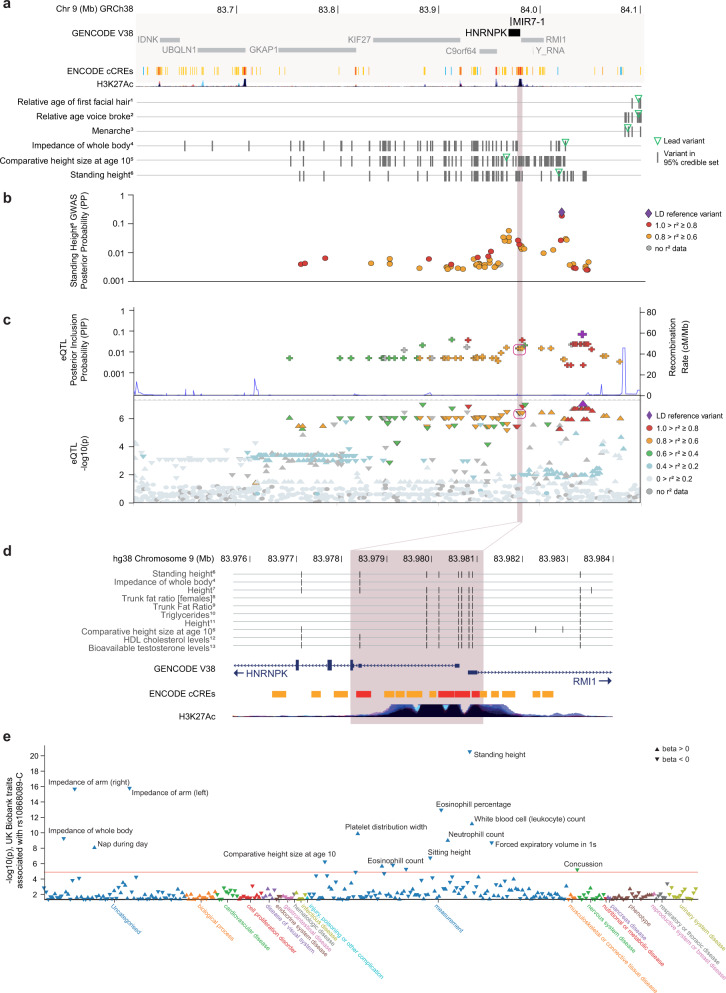
Association of human variants in the locus encompassing *MIR7-1* with height, adiposity and related traits. **a** *MIR7-1* is located in the last intron of *HNRNPK* (dark grey, GENCODE V38; chr9:83.6-84.1 Mb). Tracks display ENCODE candidate *cis-*Regulatory Elements (ENCODE cCREs^112^; red, promotor-like; orange, proximal enhancer-like; yellow: distal enhancer-like; blue: CTCF-only) and histone acetylation (H3K27Ac) tracks (UCSC browser hg38, (http://genome.ucsc.edu^113^). Tracks labelled with phenotypic traits (UK Biobank Neale v2, 2018) show the position of fine-mapped variants with Open Targets variant-to-gene (V2G) annotations for *HNRNPK* within this chromosomal region. Trait superscripts 1-6 refer to study-locus summary statistics in Supplementary Data 1b. Summary statistics for study-locus associations which had an Open Targets locus-to-gene (L2G) annotation for *HNRNPK* are provided in Supplementary Data 1. **b** Fine-mapping of GWAS locus for standing height, shown as posterior probability (PP) for each variant in the 95% credible set (UK Biobank-derived trait *NEALE2_50_raw*, lead variant chr9:84,020,284 G > C; data from Open Targets; Supplementary Data 1). The shaded area indicates a region where credible variants overlap with predicted regulatory regions immediately 5’ of *HNRNPK*. Six of these seven variants are in high pairwise LD (r^2^ > 0.95). **c** LocusZoom of the reported *cis-*eQTL for *HNRNPK* expression in monocytes (BLUEPRINT); data from FIVEx, https://fivex.sph.umich.edu. Open Targets reported evidence of colocalisation with standing height (UKB Neale v2, 2018; *coloc*^114^, H4 = 0.81, QTL beta = 0.04). eQTL minimum p-value was at rs10868089-C (chr9:84,042,868 T > C, LD reference variant). **d** Zoomed-in view showing predicted ENCODE cis-Regulatory Elements immediately 5’ of *HNRNPK* (chr9:83.976-83.984 Mb). Displays variants which are contained in a credible set of a selected trait (for UK Biobank-derived traits) or are in linkage disequilibrium (*r*^*2*^ > 0.8) with the lead variant (for non-UK Biobank studies). Trait superscripts 4-13 refer to study-locus summary statistics in Supplementary Data 1b. **e** Single-variant summary statistics (p-value and direction of effect) for the *HNRNPK* eQTL lead variant shown in (**c**) (rs10868089-C, chr9:84042868 T > C) obtained from Open Targets. Points represent UK Biobank-derived traits (UK Biobank Neale v2, 2018) with single-variant P < 0.05.

Loci linked to *HNRNPK* by the Open Targets locus-to-gene pipeline^67^ were associated with several traits including height, trunk fat ratio and triglyceride levels (GWAS threshold *P* < 5 × 10^−8^) (Fig. 6a, Supplementary Data 1). Credible sets contained 7 variants overlapping putative *HNRNPK* promoter regions immediately 5’ of the *HNRNPK* coding region (Fig. 6b). In neuronal cells, increased transcription of *Hnrnpk* induces an equivalent increase in pre-miR-7a1 and mature miR-7a levels^61^; therefore, variants affecting transcription of *HNRNPK* would similarly affect miR-7 expression. To investigate whether human noncoding variants were associated with altered expression of *HNRNPK*, we interrogated data from a series of human eQTL datasets. Among single-variant eQTL summary statistics reporting nominal association (*P* < 0.05) with *HNRNPK* expression^65^, 22 of the 24 studies showed that variants were associated with decreased expression, including four datasets derived from brain samples. Six variants overlapping the *HNRNPK* 5’ promotor region were part of the credible set (monocyte, BLUEPRINT study; posterior inclusion probability, PIP = 0.016 for each of these 6 variants, pairwise *r*^2^ > 0.9; credible set size = 72) (Fig. 6c, d); that is, evidence for a putative causal variant rather than in linkage disequilibrium (LD) with a causal variant. Open Targets reported colocalisation of this monocyte eQTL with height and comparative height at age 10. The directions of effect at these loci are consistent with decreased *HNRNPK* expression associated with increased height and trunk fat percentage (Fig. 6e). We conclude that common variants in the region upstream of *HNRNPK* are associated with decreased *HNRNPK* expression and increased height and trunk fat in humans.

Additionally, we used PhenomeXcan^66^ to interrogate gene-tissue-trait associations for the host gene *HNRNPK* and target genes *IGSF8* and *SNCA*. For each gene, we identified consensus associations across GTEx tissues and investigated the direction of effect for human traits (Supplementary Data 2). For top-ranked relevant traits, we inspected the direction of effect per tissue (Supplementary Data 2). We found that increased expression of *HNRNPK* is associated with reduced height and adiposity traits (Supplementary Data 2a–e), while increased expression of *IGSF8* tends to be associated with increased height and adiposity traits (Supplementary Data 2f). *SNCA* expression was not consistently associated with increases or decreases in height or adiposity traits (Supplementary Data 2i–j). However, the per-tissue associations of *SNCA* expression with the two top-ranked height-related traits (each showing different direction of effect in consensus analysis across tissues) suggest that higher expression of *SNCA* in some neural and endocrine tissues (including pituitary, pancreas, adipose tissues, nucleus accumbens, and basal ganglia) is associated with increased height (Supplementary Data 2k). Therefore, we found that the direction of effect for associations between variants that alter the expression of *HNRNPK* and *IGSF8*, and *SNCA* in some cell types, are consistent with the mouse phenotypes observed.

## Discussion

**Fig. 7:**
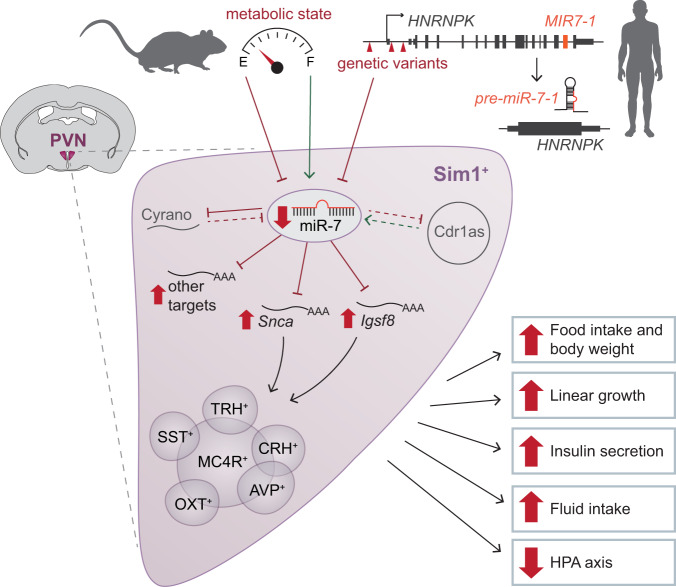
The role of miR-7 in Sim1 neurons to regulate mammalian energy homeostasis and neuroendocrine function. In mice, hypothalamic expression of miR-7 is regulated by metabolic state. In humans, variants in the locus encompassing miR-7 are associated with reduced expression of *HNRNPK/MIR-7-1*. miR-7 regulates the expression of the noncoding RNAs Cyrano and Cdr1as (dashed lines indicate previously published data^35^). Loss of miR-7 expression in Sim1 neurons leads to upregulation of miR-7 target genes, including *Snca* and *Igsf8*, to disrupt the function of multiple Sim1 neuron subpopulations. As a result, the absence of miR-7 causes hyperphagic obesity, increased linear growth, increased insulin secretion, increased fluid intake, and suppressed HPA axis function. Green arrows indicate a positive effect on expression; red blunt-headed lines indicate a negative effect on expression.

The evolutionary conservation of miR-7, the redundancy of three independently regulated miR-7-encoding genes, and its tissue-specific expression pattern suggest that this miRNA family carries out an important functional role in the hypothalamus. Here, by deploying a systematic approach to investigate the physiological consequences of *mir-7* deletion from several hypothalamic cell populations critical to the regulation of mammalian energy homeostasis, we discovered that miR-7 plays an essential role in Sim1 neurons to maintain energy homeostasis (Fig. 7). The slight weight gain observed in *Pomc-cre;mir-7*^*fl/fl*^ and *Lepr-cre;mir-7*^*fl/fl*^ mice, combined with the absence of weight gain in *Agrp-cre;mir-7*^*fl/fl*^ mice, is consistent with miR-7 coordinately regulating gene expression responses to defend against starvation, in keeping with the regulation of this microRNA by states of negative and positive energy balance.

Although several miRNAs have been implicated in peripheral metabolism, only a few studies have linked miRNAs to the hypothalamic control of energy homeostasis^68–71^. A previous study overexpressing a miR-7 sponge in POMC neurons found only a minor effect on body weight^70^, in line with our results upon genetic deletion of *mir-7* in these neurons. Here, by examining the effects of *mir-7* deletion in *Lepr, Pomc, Agrp* and *Sim1*-expressing neurons, we demonstrate that miR-7 in Sim1 neurons plays a critical role in energy balance control. The indispensability of miR-7 in the melanocortin circuit controlling body weight is remarkable, as deletion of individual miRNAs rarely causes marked phenotypes^72,73^.

The loss of miR-7 from Sim1 neurons induces severe obesity, increased energy intake, decreased energy expenditure, increased linear growth, and hyperinsulinemia due to increased insulin secretion. These phenotypes precisely mirror those seen in mice and humans with SIM1 haploinsufficiency. Amongst the different neuronal populations in the PVN, the effects of miR-7 on food intake, energy expenditure, linear growth and insulin secretion are likely to be mediated by the disrupted function of MC4R-expressing neurons (Fig. 7). These phenotypes overlap with those seen in humans (and mice) with loss-of-function mutations in MC4R, which represent the commonest monogenic cause of obesity. Similarly, disruption of genes which affect the development and/or function of hypothalamic melanocortin circuits cause obesity and increased growth in multiple species^74–76^. To that end, it is notable that the miR-7 sequence is highly conserved across zebrafish, mice, and humans and may similarly regulate the melanocortin pathway across these species. In *ob/ob* mice with leptin deficiency and an associated reduction in MC4R signalling, the observed upregulation of hypothalamic miR-7 expression may represent a compensatory mechanism to dampen the obesogenic effect of a suppressed melanocortin system.

This study demonstrates that miR-7 is required for the sexual dimorphism of body weight control, as female mice lacking miR-7 in Sim1 neurons exhibit enhanced obesity and ultimately attain the same body weight as male mice. Estrogens contribute to the sexual dimorphism of obesity susceptibility, such that males and ovariectomized females are more obesity-prone than intact female mice^77,78^. Furthermore, estrogen has recently been shown to regulate melanocortin circuits^79^. Notably, we have previously reported a reciprocal relationship between miR-7 and estradiol, in which estradiol regulates miR-7 expression^28^ and *mir-7a2*^*–/–*^ mice develop hypogonadotropic hypogonadism and suppression of circulating estradiol^27^. Further studies are warranted to determine whether miR-7 plays a role in the protective effects of estrogens on diet-induced obesity. The sex-specific effects of miR-7 depletion may also arise from the fact that Sim1 neurons themselves contribute to energy balance control in a sexually dimorphic manner. For example, it has been shown that both *Sim1* deficiency and Sim1 neuron ablation elicit greater obesity in females compared to males^10,44,80^.

*UBC-cre/ERT2; mir-7*^*fl/fl*^ mice displayed a more pronounced obesity phenotype than *Sim1-cre;mir-7*^*fl/fl*^ mice, suggesting that miR-7 in other unidentified cell population(s) or an additive effect of miR-7 in multiple hypothalamic cell types may be implicated in weight regulation. In addition to the ARC and PVN, miR-7 is also highly expressed in the suprachiasmatic nucleus (SCN) and the supraoptic nucleus (SON). The SCN is the circadian pacemaker in mammals; it controls behavioural, neuroendocrine, and autonomic functions to coordinate the daily rhythm of neural and metabolic processes^81^. As SCN disruption leads to metabolic dysfunction and obesity^82–84^, loss of miR-7 in this hypothalamic region may account for the stronger obesity phenotype of *UBC-cre/ERT2;miR-7*^*fl/fl*^ mice compared to *Sim1-cre;miR-7*^*fl/fl*^ mice. The SON is comprised of magnocellular OXT- and AVP-expressing neurons that release these neuropeptides into circulation through axon terminals in the posterior pituitary. Sim1 is expressed in neurons of both the PVN and SON, as these two populations arise from a common developmental lineage^5,43^. Thus, deletion of *mir-7* in the SON of *Sim1-cre;miR-7*^*fl/fl*^ mice may contribute to phenotypes related to OXT and AVP release, such as water intake.

Using an in vivo, Cre-dependent viral overexpression approach, we identified *Igsf8* and *Snca* as direct miR-7 targets that are individually sufficient to regulate energy homeostasis and neuroendocrine axes through Sim1 neurons. IgSF8 localizes to presynaptic axon terminals^60^ and has been recently described to regulate synaptic function in hippocampal circuits^59^. In line with our findings of increased plasma insulin upon *Igsf8* overexpression, *Igsf8* knockout mice have been reported to exhibit impaired glucose tolerance by the International Mouse Phenotyping Consortium (www.mousephenotype.org)^85^. Alpha-synuclein is known for its pathological aggregation in neurodegenerative disorders and is associated with dopaminergic neuron loss in Parkinson’s disease^86^. While its physiological function is only partly understood, accumulation of α-synuclein has been linked to ER stress, altered mitochondrial function, impaired vesicle trafficking, and synaptic dysfunction^87^. Of note, the overexpression of these two target genes did not fully recapitulate the obesity phenotype induced by *mir-7* deletion. These intermediate phenotypes likely reflect the fact that miRNAs typically act via the cumulative contributions of several target genes to simultaneously regulate numerous cellular processes in a sensitized background such as metabolic stress conditions^88^. In genetic mouse models harbouring mutated miRNA binding sites within target genes, only rare targets containing multiple binding sites tend to elicit strong phenotypes^89^, whereas most targets typically have negligible or partial contribution to the miRNA phenotypes^90^. The fact that *Snca* and *Igsf8* overexpression did not affect corticosterone levels or water intake may suggest that other targets mediate the effects of miR-7 in specific Sim1 neuron subpopulations. Alternatively, as miR-7 is known to regulate neuronal development^30^, the post-developmental timing of AAV-mediated target overexpression may have precluded the detection of important developmental roles for these targets in specific Sim1 neuron subpopulations.

By interrogating genome-wide association studies based on multiple cohorts involving >500,000 people, as well as large-scale eQTL resources, we found that variants at the *HNRNPK/MIR7-1* locus were associated with increased height and truncal fat and with other related measures such as bioelectrical impedance. We also observed associations with phenotypes that are directly modulated by the degree of adiposity, in particular the timing of puberty. Several studies have shown that variants associated with body mass index (BMI) or obesity are also associated with the timing of pubertal onset (recorded by age of menarche in females or timing of voice break in males^91,92^). These associations with the timing of puberty could also be linked to the necessity of miR-7 for the maintenance of pituitary hormone production and fertility^27,28^. Recognised limitations of current genetic resources include uncertainty in predicting causal genes at GWAS and eQTL loci^67^, a lack of validation data for the predicted direction of tissue-specific gene expression^93^, and distinguishing causal genes in the presence of regulatory pleiotropy^66,94^. Further work is therefore warranted to delineate the roles of genetic regulation by individual alleles and other mechanisms on the expression of *HNRNPK* and intron-embedded *MIR7-1*, as well as *RMI1* (located head-to-head on the opposite strand) and other proximal genes, and to assess their role in the regulation of metabolic traits. Additional studies are also needed to test whether variants in the loci surrounding *IGSF8* and *SNCA* are associated with anthropometric and/or metabolic traits and whether rare variants in these genes are associated with obesity in clinical cohorts.

In summary, we demonstrate that miR-7 regulates the hypothalamic melanocortin pathway. It is clear that quantitative variation in MC4R signalling is a major regulator of human body weight, as loss-of-function *MC4R* mutations cause obesity^13^ and gain-of-function mutations in *MC4R* protect against obesity^58^. Moreover, MC4R agonists lead to weight loss in people with obesity due to genetic disruption of the melanocortin pathway^17–19^. In view of our findings, dissection of the gene network regulated by miR-7 may reveal new insights into the regulation of mammalian energy homeostasis and identify potential targets for weight loss therapy.

## Methods

### Mouse strains and animal husbandry

All animal experiments were approved by the Kantonale Veterinäramt Zürich (Veterinary Office of the Canton of Zürich). *Pomc-cre* (B6.FVB-Tg(Pomc-cre)1Lowl/J)^95^, *Lepr-cre* (B6.129-*Lepr*^tm2(cre)Rck^/J)^96^, *Agrp-cre* (STOCK *Agrp*^tm1(cre)Lowl^/J)^97^, *Sim1-cre* (B6.FVB(129 × 1)-Tg(Sim1-cre)1Lowl/J)^43^, *UBC-cre/ERT2* (B6.Cg-*Ndor1*^Tg(UBC-cre/ERT2)1Ejb^/1J)^98^, and *Rosa26-LSL-tdTomato* (B6.Cg-*Gt(ROSA)26Sor*^tm14(CAG-tdTomato)Hze^/J)^99^ mice were purchased from The Jackson Laboratory. *Agrp-cre* mice were backcrossed five times into a C57BL/6 background before being used for experiments. Generation of *mir-7a1*^*fl/fl*^, *mir-7a2*^*fl/fl*^, *mir-7b*^*fl/fl*^ mice has been previously described^27,29^, and mice harbouring all three floxed alleles are referred to as *mir-7*^*fl/fl*^ mice. All mice were maintained on a C57BL/6 background. Mice were housed in a pathogen-free animal facility at the Institute of Molecular Health Sciences at ETH Zürich, in a temperature-controlled room (22 °C) with 55% humidity and a 12-hour light/12-hour dark cycle (lights on from 06:00 to 18:00). Mice were fed a standard laboratory chow diet (Kliba Nafag 3437; containing 3 kcal/g metabolizable energy with 4.5% fat, 18.5% protein, and 38% carbohydrate [w/w]) or high-fat diet (HFD) (SAFE diets 260HF; containing 5.5 kcal/g metabolizable energy with 36% fat, 20% protein, 37% carbohydrate, and 18% sucrose [w/w]). Feeding with HFD began at 4–5 weeks of age. Weekly body weight and blood glucose measurements were performed between 4–25 weeks of age. All other measurements were performed between 8–25 weeks of age. Mice were monitored for signs of distress at least three times per week, or twice daily during metabolic cage experiments. Euthanasia was performed by placing mice in a plastic container that was slowly flooded with carbon dioxide. After breathing stopped, the head of the mouse was removed to ensure death.

### Tamoxifen administration

Four-week-old *mir-7*^*fl/fl*^ and *UBC-cre/ERT2; mir-7*^*fl/fl*^ littermate mice were administered daily intraperitoneal injections of 2 mg tamoxifen (T5648, Sigma), dissolved at a concentration of 20 mg/ml in 10% ethanol/90% corn oil, for 5 days. In HFD-fed cohorts, feeding with HFD began one week after the last injection.

### Body composition and length

Fat and lean mass composition was measured using an EchoMRI instrument. Fat pad weights were determined by post-mortem dissection. Body length was measured as the distance from nose to base of tail.

### Metabolic cage measurements

Food intake, water intake, energy expenditure, locomotor activity, and RER were measured in up to 16 cages simultaneously using the PhenoMaster home cage phenotyping system (TSE Systems) in the ETH Phenomics Center Phenotyping Unit. After two days of habituation, measurements were recorded for 72 hours. Data are presented as both the hourly data over the 72 hour period as well as the average values across all three recorded light (06:00–18:00) and dark phases (18:00–06:00). For water intake measurements upon PBS, AVP, and DDAVP administration, injections were performed over a period of 20 minutes between 17:20 and 17:40. Mice were injected intraperitoneally with PBS, 0.16 µg/g body weight AVP (H-1775, Bachem), or 0.25 µg/g body weight DDAVP (H-7675, Bachem). Water intake was subsequently recorded for 12 h during the dark phase.

### Blood glucose measurements, ipGTT, and ipITT

Blood glucose was measured from a tail nick using a Contour XT glucometer (Bayer). For ipGTT, mice were fasted for 6 hours prior to intraperitoneal injection with D-glucose in PBS (2.5 g/kg body weight for chow-fed mice or 1.25 g/kg body weight for HFD-fed mice). For ipITT, mice were fasted for 6 hours prior to intraperitoneal injection with insulin (I9278, Sigma) in PBS (0.5 U/kg body weight for chow-fed mice or 0.7 U/kg body weight for HFD-fed mice).

### Hormone measurements in plasma and tissues

To collect tissues for hormone measurements, mice were euthanized by CO_2_ inhalation and tissues were dissected, flash frozen on dry ice, and stored at −80 °C. Tissues were homogenized in PBS supplemented with protease inhibitor (11697498001, Roche) and lysed by sonication. Protein concentration was measured by BCA assay. Blood was collected from a tail nick into a tube containing EDTA (0.5 mM final concentration) and plasma was separated by centrifugation at 8000 rcf for 8 minutes. For plasma insulin measurements, mice were fasted for 6 hours prior to blood collection.

The following assays were used for hormone measurements: ACTH Ultrasensitive lumELISA (AC562T-100, Calbiotech), Corticosterone ELISA (ADI-900-097, Enzo), Growth Hormone ELISA (EZRMGH-45K, Millipore), IGF-I/IGF-1 Quantikine ELISA (MG100, R&D Systems), and Ultrasensitive Insulin ELISA (80-INSRTU, Alpco).

### Cryosections and immunofluorescence

Mice were euthanized by CO_2_ inhalation and perfused intracardially with 10 mL of PBS, followed by 10 mL of 4% paraformaldehyde. Tissues were dissected and fixed in 4% paraformaldehyde for 24 h at 4 °C, followed by 30% sucrose for at least 24 hours at 4 °C. Samples were then frozen in O.C.T. compound (361603E, VWR), cryosectioned, mounted onto glass slides, and stored at −80 °C.

Cryosections (30 μm) were permeabilized in 95% ethanol at −20 °C for 5 minutes. For immunostainings with anti-Oxytocin or anti-V5 antibodies, antigen retrieval was performed in 10 mM sodium citrate buffer (pH 6.0) for 10 minutes at 95 °C. Sections were blocked at room temperature in PBS containing 0.1% Triton X-100, 1% bovine serum albumin, and 5% goat serum for 1 hour. Primary antibodies were applied at 4 °C overnight and secondary antibodies were applied at room temperature for 1 hour. Hoechst dye was applied to stain nuclei, and then slides were mounted using CC/Mount (C9368, Sigma).

Imaging was performed with a 20x objective on a Pannoramic 250 Slide Scanner (3D Histech). Image quantification was performed using QuPath software version 0.1.2^100^ by an experimenter who was blinded to the genotypes. Quantification of tdTomato+ and p-CREB+ cells within the PVN was performed in three sections per mouse, at 120 μm intervals between −0.7 mm to −0.96 mm to bregma^101^, and is reported as the average per mouse. P-CREB staining intensity was determined as the mean grey value within each p-CREB+ cell, from which the background value (mean grey value of p-CREB-negative regions) was subtracted.

### Quantification of microRNA and mRNA by qPCR

Mice were euthanized by CO_2_ inhalation and tissues were dissected, frozen on dry ice, and stored at −80 °C. Pancreatic islet isolation was performed as previously described^102^. RNA isolation was performed with TRI reagent (T9424, Sigma) according to the manufacturer’s protocol. For mRNA measurements, reverse transcription was performed with High Capacity cDNA Reverse Transcription kit (4368814, Applied Biosystems) and qPCR was performed with SYBR Fast Universal Mastermix (KK4600, Kapa) and gene-specific primers (listed in Supplementary Table 1) in a LC480 II Lightcycler (Roche). MicroRNA measurements were performed with TaqMan microRNA assay kits (4440887, Applied Biosystems). Measurements of mRNA were normalised to 36b4, and miRNA measurements were normalised to sno-202. Absolute quantification of miR-7a and miR-7b was determined using a spike-in of synthetic RNA comprised of the mature miR-7a or miR-7b sequences. To differentiate between absolute levels of miR-7a1 and miR-7a2, mature miR-7a was quantified in the hypothalamus of *mir-7a2*^*+/+*^ *and mir-7a2*^*–/–*^ mice.

### Neuron isolation, FACS, and RNA extraction

Hypothalami from four 8–10 week old female mice were pooled for each sample. The hypothalamus was dissected in a manner that included the entire PVN region and excluded the SON region (which also contains Sim1-expressing neurons). Tissues were dissected into cold Hibernate-A (A1247501, Gibco) and cut into small pieces with a scalpel blade. Tissues were then incubated in dissociation media (Hibernate-A minus calcium (HA-CA, Brainbits) with 2 mg/ml papain (LS003120, Worthington), 100 U/ml DNase I (LK003172, Worthington), 0.5 mM GlutaMAX (35050-038, Gibco), 5% w/v trehalose (T0167, Sigma), 0.5 mM EDTA, 5.5 mM L-cysteine, and 0.067 mM β-mercaptoethanol) for 60 minutes at 37 °C with agitation. After the papain digest, the dissociation media was replaced with pre-warmed trituration media (Hibernate A with 1x B27 supplement (17504044, Gibco), 0.5 mM GlutaMAX, and 5% w/v trehalose). Samples were triturated using a 200 μL pipette, and then pelleted by centrifugation at 100 rcf for 10 minutes. Media was replaced with fresh trituration media and the trituration and centrifugation steps were repeated. The cell pellet was washed once with FACS media (Leibowitz’s L15 medium (21083027, Gibco) with 10 mM Hepes, 1 mg/mL bovine serum albumin (126579, Calbiochem), 5% trehalose, and 50 U/mL DNase I) to remove traces of phenol red, and then was resuspended in FACS media. Prior to sorting, cells were passed through a 35 μm mesh filter (352235, Falcon). Dead cells were stained with Sytox blue (S34857, Invitrogen). Sorting was performed by the ETH Zürich Flow Cytometry Core Facility with a BD FACSAriaIII Cell Sorter. 10% of the sorted cells were used for microRNA quantification by qPCR. For RNA sequencing, the remaining 90% of tdTomato+ cells were used for RNA isolation with PicoPure RNA Isolation Kit (KIT0204, Applied Biosystems) according to manufacturers’ instructions with DNase treatment (79254, Qiagen).

### RNA sequencing and analysis

Library preparation and sequencing were performed by the Functional Genomics Center Zürich. Sequencing libraries were prepared by SmartSeq2 and sequencing was performed with the Illumina NovaSeq 6000. Raw reads were cleaned by removing adapter sequences, trimming low quality ends, and filtering reads with low quality (phred quality <20) using Trimmomatic (Version 0.36)^103^. Sequence pseudo alignment of the resulting high-quality reads to the mouse reference genome (GRCm38.p6 assembly) and quantification of gene level expression (gene models from GENCODE release M32) were carried out using Kallisto (Version 0.44)^104^. Differentially expressed (DE) genes were identified using the R package edgeR^105^ from Bioconductor Version 20, using a generalised linear model (glm) regression, a quasi-likelihood (QL) differential expression test and the trimmed means of M-values (TMM) normalisation. Predicted conserved miR-7 and miR-16 targets were retrieved from TargetScanMouse release 7.2. The criteria for target site conservation in TargetScanMouse7.2 is defined by phylogenetic branch length, with each site type having a different threshold for conservation (8mer ≥ 0.6; 7mer-m8 ≥ 1.8; 7mer-1A ≥ 2.5)^51^.

### Cell culture

SH-SY5Y cells (94030304, Sigma) were cultured in a 1:1 mixture of MEM (10370021, Gibco) and Ham’s F-12 Nutrient Mix (11765054, Gibco), supplemented with 10% fetal bovine serum (F9665, Sigma), 0.5% GlutaMAX (35050061), 0.5% sodium pyruvate (11360070, Gibco), and 1% pen strep (11548876, Gibco). GH3 cells (CCL-82.1, ATCC) were cultured in Ham’s F-12K media (21127030, Gibco) supplemented with 15% horse serum (26050088, Gibco), 2.5% fetal bovine serum (F9665, Sigma), and 1% pen strep (11548876, Gibco). Cells were plated at a density of 50,000 cells/cm^2^ for experiments.

### Luciferase assay

To assess the interaction of miR-7 with the 3’UTR of *Igsf8*, we utilized the pmirGLO vector (E1330, Promega), which expresses firefly luciferase (as an experimental reporter that is subject to the effect of miRNA regulation) and *Renilla* luciferase (as an internal control). 160 bp fragments of the *Igsf8* 3’UTR containing either the WT (GTCTTCCA) or mutated (GCCCTTCA) binding site for miR-7 were *do novo* synthesized (Genscript; sequences listed in Supplementary Table 2) and cloned into SacI/XbaI sites of pmirGLO. GH3 cells on poly-D-lysine coated plates (P6407, Sigma) were transduced with miR-7a2 and control recombinant adenoviruses^29^, and then were transfected 24 hours later with pmirGLO-Igsf8-WT or pmirGLO-Igsf8-Mut using Lipofectamine 2000 (11668019, Invitrogen). Cells were harvested and assayed 24 hours post-transfection using the Dual-Luciferase Reporter Assay System (Promega). Firefly luciferase activity was normalized to the activity of the *Renilla* luciferase control.

### Target gene AAV production and administration

pAAV-FLEx-GFP was a gift from Edward Boyden (Addgene plasmid #28304). Annealed oligo cloning was performed to replace the GFP sequence with new multiple cloning sites containing SpeI/NsiI sites and an N-terminal or C-terminal V5 tag (oligo sequences listed in Supplementary Table 3). Target genes were PCR amplified from cDNA of mouse brain (primers listed in Supplementary Table 3) and inserted into the SpeI/NsiI sites of the pAAV-FLEx-N-V5 or pAAV-FLEx-C-V5 vector. The resulting plasmids were transfected with or without a Cre-expressing plasmid (gift from Tyler Jacks; Addgene plasmid #17408) into GH3 cells using Lipofectamine 2000 (11668019, Invitrogen) and were harvested 48 hours post-transfection for verification of V5 expression by Western blot. Production of AAV-PHP.eB viral vectors was performed by the EPFL Bertarelli Foundation Gene Therapy Platform. For each construct, AAV-PHP.eB vectors were generated by transient transfection of the HEKExpress XLG1.0 cell line (ExcellGene SA) maintained in suspension in Freestyle F17 medium supplemented with 4 mM GlutaMAX (ThermoFisher), as previously described^106^. The pUCmini-iCAP-PHP.eB was a gift from Viviana Gradinaru (Addgene plasmid #103005). Transfection conditions were defined as previously described^107^. For vector production, cells were maintained for 7 days after transfection in medium supplemented with 4 mM valproic acid (Sigma). Cell supernatant was collected on days 4 and 7 after transfection and the cell pellet was collected on day 7. DNA present in the cell pellet was eliminated by SAN nuclease digestion (ArcticZymes). For downstream purification, AAV particles present in both the cell pellet and culture medium were isolated by affinity chromatography using an AKTA Pure chromatography apparatus (GE Healthcare) connected to a 5 mL pre-packed POROS CaptureSelect AAV9 column (ThermoFisher). Buffer exchange (PBS supplemented with 0.001% Pluronic F-68 (Gibco)) and vector concentration were performed using a 100 kDa cut-off Amicon Ultra-15 device (Millipore). The final concentration of genome-containing AAV particles in the vector suspension was determined by digital PCR (QIAcuity Digital PCR System, QIAGEN) using TaqMan primers specific for the WPRE sequence. AAV vectors were administered intravenously to 3–4 week-old mice at a dose of 2.5 × 10^11^ vg/mouse.

### Western blot

Cells were washed once with PBS, then lysed in RIPA buffer (150 nM NaCl, 1% Triton-X, 50 mM Tris, 0.5% sodium deoxycholate, 0.1% SDS) supplemented with cOmplete, EDTA-free Protease inhibitors (11873580001, Roche). Protein concentrations were measured by bicinchoninic acid assay. Proteins were separated by SDS-PAGE, transferred to nitrocellulose membranes, and blocked in 5% milk in Tris-buffered saline with Tween-20 (TBS-T) for 1 h. Membranes were incubated with primary antibody at 4 °C overnight followed by secondary antibody at room temperature for 1 h, and were developed using ECL Western Blotting Substrate. For protein quantification, densitometry was performed using ImageJ version 1.53c and values were normalised to GAPDH. Uncropped images of blots are provided in the Source Data file.

### Antibodies

The following antibodies were used for immunofluorescence: guinea pig anti-insulin (A056401, Dako, 1:1000), rabbit anti-phospho-CREB (Ser133) (9198, Cell Signaling, 1:250), rabbit anti-Oxytocin (T-4084, Peninsula Laboratories, 1:1000), mouse anti-V5 tag (R960-25, Invitrogen, 1:250), goat anti-guinea pig conjugated to Alexa Fluor 488 (A11073, Invitrogen, 1:500), goat anti-rabbit conjugated to Cy5 (A10523, Invitrogen, 1:500), and goat anti-mouse conjugated to Alexa647 (115-605-003, Jackson ImmunoResearch, 1:500). The following antibodies were used for Western blot: mouse anti-V5 tag (R960-25, Invitrogen, 1:5000), goat anti-IgSF8 (AF3117, R&D Systems, 1:500), mouse anti-Alpha-synuclein (610786, BD Biosciences, 1:500), rabbit anti-Beta-arrestin (ab32099, Abcam, 1:1000), rabbit anti-c-Raf (53745, Cell Signalling, 1:500), rabbit anti-GAPDH (2118, Cell Signalling, 1:1000), goat anti-rabbit conjugated to HRP (401393, Sigma, 1:10,000), rabbit anti-goat conjugated to HRP (401515, Sigma, 1:10,000), and goat anti-mouse conjugated to HRP (401253, Sigma, 1:10,000).

### Statistics

Statistical analysis was performed using GraphPad Prism 7. Data are presented as mean ± standard deviation (where error bars are present). Statistical tests are indicated in the figure legends. Sample size (n) represents number of animals or number of wells in a multi-well culture plate, unless otherwise stated. A *p* value of less than 0.05 was considered statistically significant.

### Open Targets Genetics GWAS loci and gene prioritization

We defined a list of relevant human traits (anthropometry, adiposity, puberty and other related traits) prior to selection and analysis of GWAS loci. We used the pre-computed Open Targets Genetics (Version 5)^64^ locus-to-gene (L2G) resource^67^ to obtain all GWAS loci (locus-trait associations, *p* < 10^−8^) that had an L2G annotation for *HNRNPK*. Gene prioritization scores and Partial gene prioritization scores were inspected at each study-locus for distance, eQTL, colocalisation and other evidence used by the L2G pipeline to prioritize genes at a GWAS locus (https://genetics.opentargets.org/study-locus/[Study-ID]/[lead-variant-ID]). Fine-mapping results (credible sets, available for UK Biobank Neale v2 GWAS^108^) and linkage disequilibrium (LD) with respect to lead variants were downloaded from Open Targets LocusPlot variant-to-gene (V2G) tables.

### FIVEx eQTL repository

We obtained eQTL summary statistics and credible sets from the FIVEx (Functional Interpretation and Visualization of Expression) instance at https://fivex.sph.umich.edu^65^ (accessed 25 October 2021), which is based on data from the EBI eETL Catalogue^109^. In this FIVEx instance, credible sets (posterior inclusion probability, PIP) are calculated using SuSiE^110^, which aims to highlight significantly associated variants in the presence of high LD. We used the FIVEx gene-search facility for *HNRNPK* cis-eQTLs and found one reported significant eQTL (BLUEPRINT: monocyte). We used the LocusZoom utility in FIVEx to obtain PIP and -log(P) plots for the credible set. Linkage disequilibrium is displayed using the European (EUR) population. Single- variant *cis*-eQTL nominal associations (P < 0.05) with *HNRNPK* expression were obtained from the FIVEx single-variant view for variant rs796004 C > T (chr9:83,979,883 C > T).

### PhenomeXcan analysis of genes-tissues-traits in human biobanks

We interrogated the PhenomeXcan repository^66^ (http://phenomexcan.org) which aims to prioritize likely causal gene-trait associations by synthesizing GWAS on 4,091 traits with transcriptome regulation data from 49 tissues in GTEx v8. The GWAS traits are predominantly from the Neale v2 GWAS resource^108^ using UK Biobank data^111^. For each gene (host gene *HNRNPK*, and target genes *IGSF8* and *SNCA*) we performed a *PhenomeXscan* search (*P* value<0.05) using two different thresholds of regional colocalization probability, RCP > 0.1 and RCP > 0.01 (less conservative)^66^. Among top-ranked traits (by p-value) with a relevant phenotype, we performed a *PhenomeXscan_SingleTissue* search among all tissues (*p* < 0.05) and inspected the direction of effect in each tissue.

### Reporting summary

Further information on research design is available in the Nature Research Reporting Summary linked to this article.

## Supplementary information

Supplementary Information

Peer Review File

Description of Additional Supplementary Files

Supplementary Data 1

Supplementary Data 2

Reporting Summary

## Data Availability

RNA sequencing data have been deposited to the European Nucleotide Archive (accession ID PRJEB48660). The following third-party data sources were used in this study: mouse reference genome GRCm38.p6 assembly (https://www.ncbi.nlm.nih.gov/assembly/GCF_000001635.26/), Open Targets Genetics (https://genetics.opentargets.org), FIVEx (https://fivex.sph.umich.edu), PhenomeXcan (http://phenomexcan.org), PhenomeXcan fastENLOC results (https://github.com/hakyimlab/phenomexcan), PrediXcan GTEx v8 expression predictors (https://www.predictdb.org), GTEx v8 eQTLs (accessible via https://www.gtexportal.org/home/datasets). All other data generated or analysed during this study are included in this published article (and its supplementary information files). Source data are provided with this paper.

## Source data

Source data

## References

[1] Gautron L, Elmquist JK, Williams KW (2015). Neural control of energy balance: Translating circuits to therapies. Cell.

[2] Clemmensen C (2017). Gut-Brain Cross-Talk in Metabolic Control. Cell.

[3] Friedman JM (2019). Leptin and the endocrine control of energy balance. Nat. Metab..

[4] Yeo, G. S. H. et al. The melanocortin pathway and energy homeostasis: From discovery to obesity therapy. *Mol. Metab*. 101206 (2021). 10.1016/j.molmet.2021.101206 PMC8050006 33684608

[5] Michaud JL, Rosenquist T, May NR, Fan CM (1998). Development of neuroendocrine lineages requires the bHLH-PAS transcription factor SIM1. Genes Dev..

[6] Li MM (2019). The Paraventricular Hypothalamus Regulates Satiety and Prevents Obesity via Two Genetically Distinct Circuits. Neuron.

[7] Gold RM, Jones AP, Sawchenko PE, Kapatos G (1977). Paraventricular area: Critical focus of a longitudinal neurocircuitry mediating food intake. Physiol. Behav..

[8] Michaud JL (2001). Sim1 haploinsufficiency causes hyperphagia, obesity and reduction of the paraventricular nucleus of the hypothalamus. Hum. Mol. Genet.

[9] Kublaoui BM, Gemelli T, Tolson KP, Wang Y, Zinn AR (2008). Oxytocin Deficiency Mediates Hyperphagic Obesity of Sim1 Haploinsufficient Mice. Mol. Endocrinol..

[10] Tolson KP (2010). Postnatal Sim1 deficiency causes hyperphagic obesity and reduced Mc4r and oxytocin expression. J. Neurosci..

[11] Holder JL (2004). Sim1 gene dosage modulates the homeostatic feeding response to increased dietary fat in mice. Am. J. Physiol. - Endocrinol. Metab..

[12] Ramachandrappa S (2013). Rare variants in single-minded 1 (SIM1) are associated with severe obesity. J. Clin. Invest..

[13] Farooqi IS (2003). Clinical Spectrum of Obesity and Mutations in the Melanocortin 4 Receptor Gene. N. Engl. J. Med..

[14] Huszar D (1997). Targeted disruption of the melanocortin-4 receptor results in obesity in mice. Cell.

[15] Van Der Klaauw AA, Farooqi IS (2015). The hunger genes: Pathways to obesity. Cell.

[16] Loos, R. J. F. & Yeo, G. S. H. The genetics of obesity: from discovery to biology. *Nat. Rev. Genet*. (2021). 10.1038/s41576-021-00414-z 10.1038/s41576-021-00414-z PMC8459824 34556834

[17] Kühnen P (2016). Proopiomelanocortin Deficiency Treated with a Melanocortin-4 Receptor Agonist. N. Engl. J. Med..

[18] Clément K (2018). MC4R agonism promotes durable weight loss in patients with leptin receptor deficiency. Nat. Med..

[19] Clément K (2020). Efficacy and safety of setmelanotide, an MC4R agonist, in individuals with severe obesity due to LEPR or POMC deficiency: single-arm, open-label, multicentre, phase 3 trials. Lancet Diabetes Endocrinol..

[20] Barroso I, McCarthy MI (2019). The Genetic Basis of Metabolic Disease. Cell.

[21] French JD, Edwards SL (2020). The Role of Noncoding Variants in Heritable Disease. Trends Genet.

[22] Friedman RC, Farh KKH, Burge CB, Bartel DP (2009). Most mammalian mRNAs are conserved targets of microRNAs. Genome Res.

[23] Prochnik SE, Rokhsar DS, Aboobaker AA (2007). Evidence for a microRNA expansion in the bilaterian ancestor. Dev. Genes Evol..

[24] Farh KK-H (2005). The widespread impact of mammalian MicroRNAs on mRNA repression and evolution. Science.

[25] Landgraf P (2007). A Mammalian microRNA Expression Atlas Based on Small RNA Library Sequencing. Cell.

[26] Correa-Medina M (2009). MicroRNA miR-7 is preferentially expressed in endocrine cells of the developing and adult human pancreas. Gene Expr. Patterns.

[27] Ahmed K (2017). Loss of microRNA-7a2 induces hypogonadotropic hypogonadism and infertility. J. Clin. Invest.

[28] LaPierre MP (2021). MicroRNA-7a2 Regulates Prolactin in Developing Lactotrophs and Prolactinoma Cells. Endocrinology.

[29] Latreille M (2014). MicroRNA-7a regulates pancreatic β cell function. J. Clin. Invest..

[30] Pollock A, Bian S, Zhang C, Chen Z, Sun T (2014). Growth of the developing cerebral cortex is controlled by MicroRNA-7 through the p53 pathway. Cell Rep..

[31] McMillan KJ (2017). Loss of MicroRNA-7 Regulation Leads to α-Synuclein Accumulation and Dopaminergic Neuronal Loss In Vivo. Mol. Ther..

[32] Bak M (2008). MicroRNA expression in the adult mouse central nervous system. RNA.

[33] Herzer S, Silahtaroglu A, Meister B (2012). Locked Nucleic Acid-Based In Situ Hybridisation Reveals miR-7a as a Hypothalamus-Enriched MicroRNA with a Distinct Expression Pattern. J. Neuroendocrinol..

[34] Bartel DP (2018). Metazoan MicroRNAs. Cell.

[35] Kleaveland B, Shi CY, Stefano J, Bartel DP (2018). A Network of Noncoding Regulatory RNAs Acts in the Mammalian Brain. Cell.

[36] Piwecka M (2017). Loss of a mammalian circular RNA locus causes miRNA deregulation and affects brain function. Science.

[37] Sinden DS (2019). Knockout of the X-linked Fgf13 in the hypothalamic paraventricular nucleus impairs sympathetic output to brown fat and causes obesity. FASEB J..

[38] Granneman JG, Lahners KN (1992). Differential adrenergic regulation of beta 1- and beta 3-adrenoreceptor messenger ribonucleic acids in adipose tissues. Endocrinology.

[39] Fan W (2000). The Central Melanocortin System Can Directly Regulate Serum Insulin Levels. Endocrinology.

[40] Kublaoui BM, Holder JL, Gemelli T, Zinn AR (2006). Sim1 Haploinsufficiency Impairs Melanocortin-Mediated Anorexia and Activation of Paraventricular Nucleus Neurons. Mol. Endocrinol..

[41] Greenfield JR (2009). Modulation of Blood Pressure by Central Melanocortinergic Pathways. N. Engl. J. Med..

[42] Martinelli CE (2011). Obesity due to melanocortin 4 receptor (MC4R) deficiency is associated with increased linear growth and final height, fasting hyperinsulinemia, and incompletely suppressed growth hormone secretion. J. Clin. Endocrinol. Metab..

[43] Balthasar N (2005). Divergence of melanocortin pathways in the control of food intake and energy expenditure. Cell.

[44] Tolson KP (2014). Inducible neuronal inactivation of Sim1 in adult mice causes hyperphagic obesity. Endocrinology.

[45] Harris M (2001). Transcriptional regulation of the thyrotropin-releasing hormone gene by leptin and melanocortin signaling. J. Clin. Invest..

[46] Sarkar S, Légrádi G, Lechan RM (2002). Intracerebroventricular administration of α-melanocyte stimulating hormone increases phosphorylation of CREB in TRH- and CRH-producing neurons of the hypothalamic paraventricular nucleus. Brain Res.

[47] Caruso C (2012). Melanocortin 4 receptor activation induces brain-derived neurotrophic factor expression in rat astrocytes through cyclic AMP - Protein kinase A pathway. Mol. Cell. Endocrinol..

[48] Chiappini F, Cunha LL, Harris JC, Hollenberg AN (2011). Lack of cAMP-response Element-binding Protein 1 in the Hypothalamus Causes Obesity. J. Biol. Chem..

[49] Sutton AK (2014). Control of food intake and energy expenditure by Nos1 neurons of the paraventricular hypothalamus. J. Neurosci..

[50] Pei H, Sutton AK, Burnett KH, Fuller PM, Olson DP (2014). AVP neurons in the paraventricular nucleus of the hypothalamus regulate feeding. Mol. Metab..

[51] Agarwal V, Bell GW, Nam J-W, Bartel DP (2015). Predicting effective microRNA target sites in mammalian mRNAs. Elife.

[52] Choi J-W (2013). MicroRNA profiling in the mouse hypothalamus reveals oxytocin-regulating microRNA. J. Neurochem..

[53] Guo H, Ingolia NT, Weissman JS, Bartel DP (2010). Mammalian microRNAs predominantly act to decrease target mRNA levels. Nature.

[54] Chan KY (2017). Engineered AAVs for efficient noninvasive gene delivery to the central and peripheral nervous systems. Nat. Neurosci..

[55] Luoni M (2020). Whole brain delivery of an instability-prone Mecp2 transgene improves behavioral and molecular pathological defects in mouse models of Rett syndrome. Elife.

[56] Breit A (2006). The natural inverse agonist agouti-related protein induces arrestin-mediated endocytosis of melanocortin−3 and −4 receptors. J. Biol. Chem..

[57] Shinyama H, Masuzaki H, Fang H, Flier JS (2003). Regulation of melanocortin-4 receptor signaling: Agonist-mediated desensitization and internalization. Endocrinology.

[58] Lotta LA (2019). Human Gain-of-Function MC4R Variants Show Signaling Bias and Protect against Obesity. Cell.

[59] Apóstolo N (2020). Synapse type-specific proteomic dissection identifies IgSF8 as a hippocampal CA3 microcircuit organizer. Nat. Commun..

[60] Ray A, Treloar HB (2012). IgSF8: A developmentally and functionally regulated cell adhesion molecule in olfactory sensory neuron axons and synapses. Mol. Cell. Neurosci..

[61] Fernández-de Frutos M (2019). MicroRNA 7 Impairs Insulin Signaling and Regulates Aβ Levels through Posttranscriptional Regulation of the Insulin Receptor Substrate 2, Insulin Receptor, Insulin-Degrading Enzyme, and Liver X Receptor Pathway. Mol. Cell. Biol..

[62] Choudhury NR (2013). Tissue-specific control of brain-enriched miR-7 biogenesis. Genes Dev..

[63] Kumar, S., Velasco, A. D. R. & Michlewski, G. Oleic Acid induces miR-7 processing through remodelling of pri-miR-7/protein complex. *J. Mol. Biol*. (2017).10.1016/j.jmb.2017.05.001 10.1016/j.jmb.2017.05.001 PMC5462424 28483648

[64] Ghoussaini M (2021). Open Targets Genetics: Systematic identification of trait-associated genes using large-scale genetics and functional genomics. Nucleic Acids Res.

[65] Kwong, A. et al. FIVEx: an interactive eQTL browser across public datasets. *Bioinformatics* btab614 (2021). 10.1093/bioinformatics/btab614 10.1093/bioinformatics/btab614 PMC8723151 34459872

[66] Pividori M (2020). PhenomeXcan: Mapping the genome to the phenome through the transcriptome. Sci. Adv..

[67] Mountjoy, E. et al. An open approach to systematically prioritize causal variants and genes at all published human GWAS trait-associated loci. *Nat. Genet*. (2021). 10.1038/s41588-021-00945-5 10.1038/s41588-021-00945-5 PMC7611956 34711957

[68] Vinnikov IA (2014). Hypothalamic miR-103 Protects from Hyperphagic Obesity in Mice. J. Neurosci..

[69] Crépin D (2014). The over-expression of miR-200a in the hypothalamus of ob/ob mice is linked to leptin and insulin signaling impairment. Mol. Cell. Endocrinol..

[70] Gao Y (2019). MicroRNA miR-7 and miR-17-92 in the Arcuate Nucleus of Mouse Hypothalamus Regulate Sex-Specific Diet-Induced Obesity. Mol. Neurobiol..

[71] Ma Y (2022). Neuronal miR-29a protects from obesity in adult mice. Mol. Metab..

[72] Ebert MS, Sharp PA (2012). Roles for MicroRNAs in Conferring Robustness to Biological Processes. Cell.

[73] Miska EA (2007). Most Caenorhabditis elegans microRNAs are individually not essential for development or viability. PLoS Genet.

[74] van der Klaauw AA (2019). Human Semaphorin 3 Variants Link Melanocortin Circuit Development and Energy Balance. Cell.

[75] Sebag JA, Zhang C, Hinkle PM, Bradshaw AM, Cone RD (2013). Developmental Control of the Melanocortin-4 Receptor by MRAP2 Proteins in Zebrafish. Science.

[76] Asai M (2013). Loss of function of the melanocortin 2 receptor accessory protein 2 is associated with mammalian obesity. Science.

[77] Rogers NH, Li JWP, Strissel KJ, Obin MS, Greenberg AS (2009). Reduced energy expenditure and increased inflammation are early events in the development of ovariectomy-induced obesity. Endocrinology.

[78] Shi H, Seeley RJ, Clegg DJ (2009). Sexual differences in the control of energy homeostasis. Front. Neuroendocrinol..

[79] Krause WC (2021). Oestrogen engages brain MC4R signalling to drive physical activity in female mice. Nature.

[80] Xi, D., Gandhi, N., Lai, M. & Kublaoui, B. M. Ablation of Sim1 neurons causes obesity through hyperphagia and reduced energy expenditure. *PLoS One* **7**, e36453 (2012). 10.1371/journal.pone.0036453 PMC3338647 22558467

[81] Hastings MH, Maywood ES, Brancaccio M (2018). Generation of circadian rhythms in the suprachiasmatic nucleus. Nat. Rev. Neurosci..

[82] Adlanmerini M (2021). REV-ERB nuclear receptors in the suprachiasmatic nucleus control circadian period and restrict diet-induced obesity. Sci. Adv..

[83] Grippo RM (2020). Dopamine Signaling in the Suprachiasmatic Nucleus Enables Weight Gain Associated with Hedonic Feeding. Curr. Biol..

[84] Coomans CP (2013). The suprachiasmatic nucleus controls circadian energy metabolism and hepatic insulin sensitivity. Diabetes.

[85] Dickinson ME (2016). High-throughput discovery of novel developmental phenotypes. Nature.

[86] Bridi JC, Hirth F (2018). Mechanisms of α-Synuclein Induced Synaptopathy in Parkinson’s Disease. Front. Neurosci..

[87] Bernal-Conde LD (2020). Alpha-Synuclein Physiology and Pathology: A Perspective on Cellular Structures and Organelles. Front. Neurosci..

[88] Peláez N, Carthew RW (2012). Biological robustness and the role of microRNAs: a network perspective. Curr. Top. Dev. Biol..

[89] Title, A. C. et al. Genetic dissection of the miR-200–Zeb1 axis reveals its importance in tumor differentiation and invasion. *Nat. Commun*. **9**, 4671 (2018). 10.1038/s41467-018-07130-z PMC6220299 30405106

[90] Goga A (2021). miR-802 regulates Paneth cell function and enterocyte differentiation in the mouse small intestine. Nat. Commun..

[91] Perry JRB (2014). Parent-of-origin-specific allelic associations among 106 genomic loci for age at menarche. Nature.

[92] Elks CE (2010). Thirty new loci for age at menarche identified by a meta-analysis of genome-wide association studies. Nat. Genet..

[93] Li B (2018). Evaluation of PrediXcan for prioritizing GWAS associations and predicting gene expression. Pac. Symp. Biocomput.

[94] GTEx Consortium. (2020). The GTEx Consortium atlas of genetic regulatory effects across human tissues. Science.

[95] McHugh TJ (2007). Dentate Gyrus NMDA Receptors Mediate Rapid Pattern Separation in the Hippocampal Network. Science.

[96] DeFalco J (2001). Virus-assisted mapping of neural inputs to a feeding center in the hypothalamus. Science.

[97] Tong Q, Ye C-P, Jones JE, Elmquist JK, Lowell BB (2008). Synaptic release of GABA by AgRP neurons is required for normal regulation of energy balance. Nat. Neurosci..

[98] Ruzankina Y (2007). Deletion of the Developmentally Essential Gene ATR in Adult Mice Leads to Age-Related Phenotypes and Stem Cell Loss. Cell Stem Cell.

[99] Madisen L (2010). A robust and high-throughput Cre reporting and characterization system for the whole mouse brain. Nat. Neurosci..

[100] Bankhead P (2017). QuPath: Open source software for digital pathology image analysis. Sci. Rep..

[101] Paxinos, G. & Franklin, K. *The Mouse Brain in Stereotaxic Coordinates*. (Academic, 2001).

[102] Zmuda, E. J., Powell, C. A. & Hai, T. A Method for Murine Islet Isolation and Subcapsular Kidney Transplantation. *J. Vis. Exp*. e2096 (2011). 10.3791/2096 10.3791/2096 PMC3169267 21525838

[103] Bolger AM, Lohse M, Usadel B (2014). Trimmomatic: a flexible trimmer for Illumina sequence data. Bioinformatics.

[104] Bray NL, Pimentel H, Melsted P, Pachter L (2016). Near-optimal probabilistic RNA-seq quantification. Nat. Biotechnol..

[105] Robinson MD, McCarthy DJ, Smyth GK (2010). edgeR: a Bioconductor package for differential expression analysis of digital gene expression data. Bioinformatics.

[106] Blessing D (2019). Scalable Production of AAV Vectors in Orbitally Shaken HEK293 Cells. Mol. Ther. - Methods Clin. Dev..

[107] Challis RC (2019). Systemic AAV vectors for widespread and targeted gene delivery in rodents. Nat. Protoc..

[108] Neale, B. M. UK Biobank GWAS Results. http://www.nealelab.is/uk-biobank/. http://www.nealelab.is/uk-biobank/http://www.nealelab.is/uk-biobank/ (2018).

[109] Kerimov N (2021). A compendium of uniformly processed human gene expression and splicing quantitative trait loci. Nat. Genet..

[110] Wang G, Sarkar A, Carbonetto P, Stephens M (2020). A simple new approach to variable selection in regression, with application to genetic fine mapping. J. R. Stat. Soc. Ser. B Stat. Methodol..

[111] Bycroft C (2018). The UK Biobank resource with deep phenotyping and genomic data. Nature.

[112] ENCODE Project Consortium. (2020). Expanded encyclopaedias of DNA elements in the human and mouse genomes. Nature.

[113] Kent WJ (2002). The Human Genome Browser at UCSC. Genome Res.

[114] Giambartolomei C (2014). Bayesian Test for Colocalisation between Pairs of Genetic Association Studies Using Summary Statistics. PLoS Genet.

