# Supplementary material for: MicroRNA-7 regulates melanocortin circuits involved in mammalian energy homeostasis
Source: Nat Commun. 2022 Sep 29;13:5733. doi: 10.1038/s41467-022-33367-w (PMC9522793; doi:10.1038/s41467-022-33367-w)
Supplement: Supplementary file 1 — Supplementary Information [file 41467_2022_33367_MOESM1_ESM.pdf]

SUPPLEMENTARY FIGURES

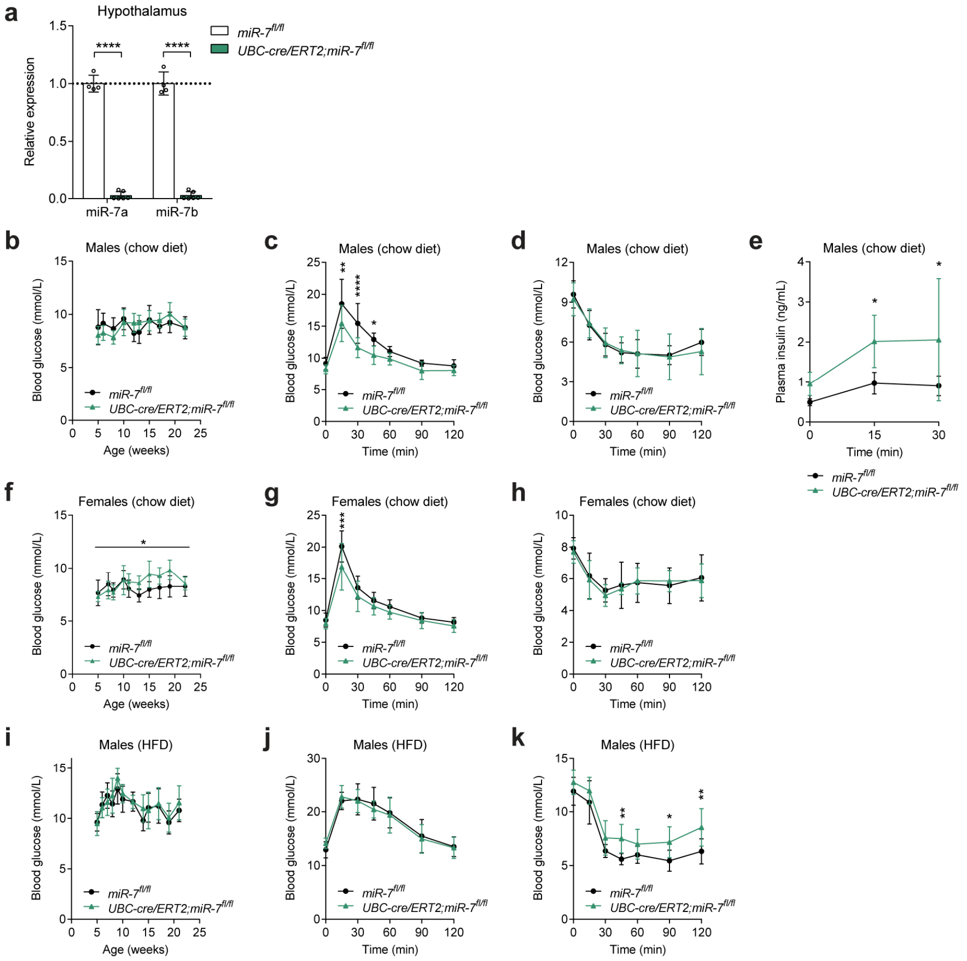

Supplementary Figure 1. Metabolic phenotypes of *UBC-cre/ERT2;mir-7$^{fl/fl}$* mice.

(a) Hypothalamic miR-7a and miR-7b expression in *mir-7$^{fl/fl}$* and *UBC-cre/ERT2;mir-7$^{fl/fl}$* mice (relative to *mir-7$^{fl/fl}$*) (n = 4 and 6 animals).

(b–e) Random fed blood glucose levels (n = 8 and 10 animals) (b), blood glucose levels during an ipGTT (n = 8 and 10 animals) (c), blood glucose levels during an ipITT (n = 8 and 10 animals) (d), and plasma insulin levels during an ipGTT (n = 6 and 8 animals) (e) in chow-fed male *mir-7$^{fl/fl}$* and *UBC-cre/ERT2;mir-7$^{fl/fl}$* mice.

(f–h) Random fed blood glucose levels (n = 12 and 8 animals) (f), blood glucose levels during an ipGTT (n = 12 and 8 animals) (g), and blood glucose levels during an ipITT (n = 12 and 8 animals) (h) in chow-fed female *mir-7$^{fl/fl}$* and *UBC-cre/ERT2;mir-7$^{fl/fl}$* mice.

(i–k) Random fed blood glucose levels (n = 12 and 8 animals) (i), blood glucose levels during an ipGTT (n = 12 and 8 animals) (j), and blood glucose levels during an ipITT (n = 12 and 8 animals) (k) in HFD-fed male *mir-7$^{fl/fl}$* and *UBC-cre/ERT2;mir-7$^{fl/fl}$* mice.

Data are presented as mean ± SD. *P < 0.05; **P < 0.01; ***P < 0.001; no asterisk indicates P > 0.05; two-tailed *t* test (a), 2-way repeated measures ANOVA (b, f, i), or 2-way repeated measures ANOVA with Sidak's multiple comparisons test (c, d, e, g, h, j, k). Source data are provided as a Source Data file.

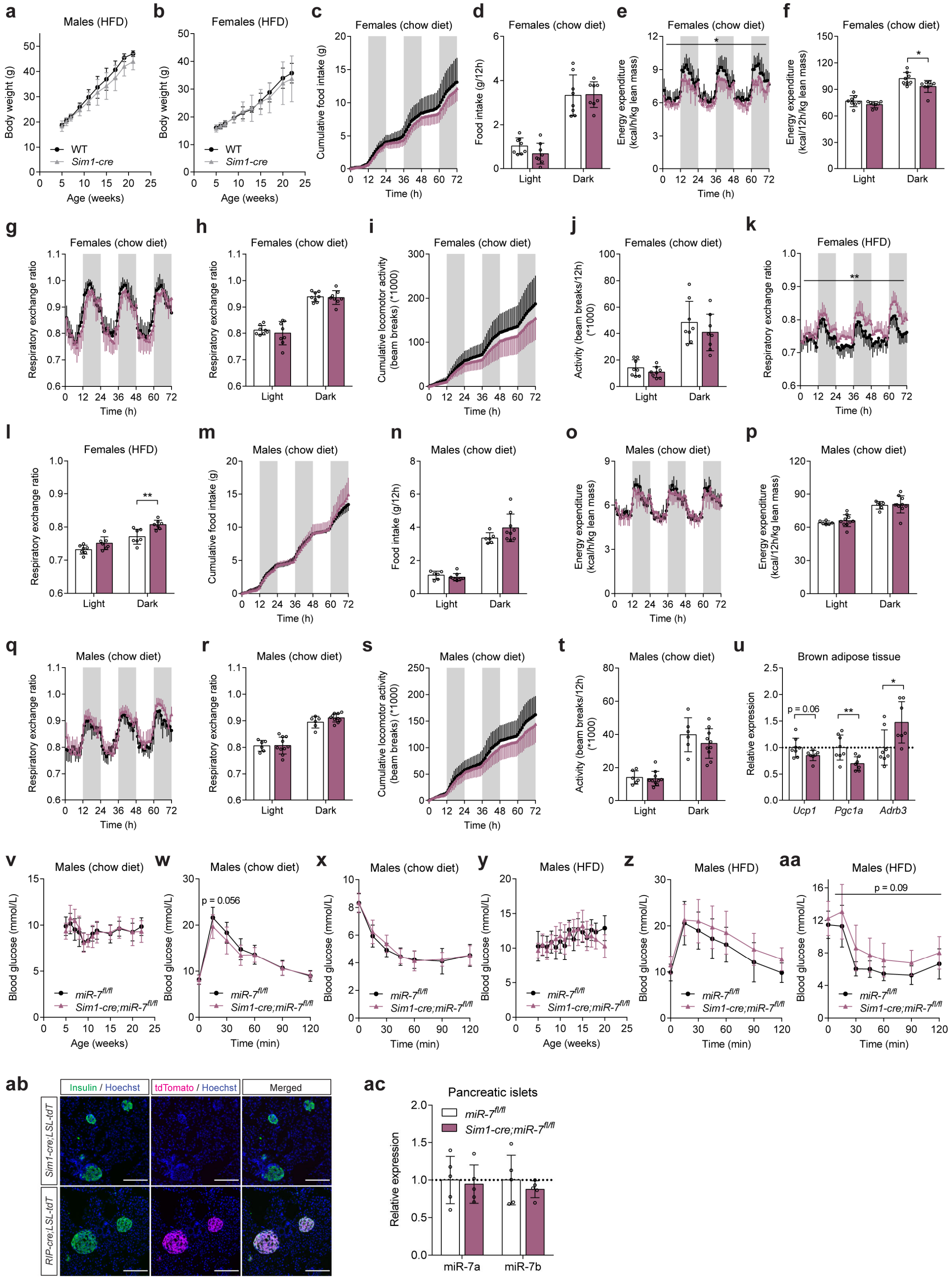

Supplementary Figure 2. Metabolic phenotypes of *Sim1-cre;mir-7$^{fl/fl}$* mice. ***(continued on next page)***

Supplementary Figure 2. Metabolic phenotypes of *Sim1-cre;mir-7$^{fl/fl}$* mice (*continued*).

(a–b) Body weight curves of HFD-fed male (n = 5 and 6 animals) (a) and HFD-fed female (n = 7 and 6 animals) (b) wild-type and *Sim1-cre* mice.

(c–t) 72-hour metabolic cage measurements of chow-fed female (n = 8 animals) (c-j), HFD-fed female (n = 7 animals) (k-l), and chow-fed male (n = 6 and 10 animals) (m-t) *mir-7$^{fl/fl}$* and *Sim1-cre;mir-7$^{fl/fl}$* mice. (c, m) cumulative food intake; (d, n) average food intake per 12-hour phase; (e, o) hourly energy expenditure; (f, p) average energy expenditure per 12-hour phase; (g, k, q) hourly RER; (h, l, r) average RER per 12-hour phase; (i, s) cumulative locomotor activity; (j, t) average locomotor activity per 12-hour phase. Dark phases are shaded in grey.

(u) *Ucp1*, *Pgc1a*, and *Adrb3* expression (relative to *mir-7$^{fl/fl}$*) in iBAT of *mir-7$^{fl/fl}$* and *Sim1-cre;mir-7$^{fl/fl}$* mice (n = 8 and 7 animals).

(v–x) Random fed blood glucose levels (n = 15 and 13 animals) (v), blood glucose levels during an ipGTT (n = 15 and 13 animals) (w), and blood glucose levels during an ipITT (n = 15 and 12 animals) (x) in chow-fed male *mir-7$^{fl/fl}$* and *Sim1-cre;mir-7$^{fl/fl}$* mice.

(y–aa) Random fed blood glucose levels (n = 10 and 19 animals) (y), blood glucose levels during an ipGTT (n = 6 and 13 animals) (z), and blood glucose levels during an ipITT (n = 6 and 13 animals) (aa) in HFD-fed male *mir-7$^{fl/fl}$* and *Sim1-cre;mir-7$^{fl/fl}$* mice.

(ab) Immunofluorescence of insulin and tdTomato in the pancreatic islets of *Sim1-cre;LSL-tdTomato* mice (representative of 3 mice), with *RIP-cre;LSL-tdTomato* (representative of 1 mouse) as a positive control (scale bar: 150 µm). Green, insulin; magenta, tdTomato; blue, Hoechst.

(ac) miR-7a and miR-7b expression (relative to *mir-7$^{fl/fl}$*) in pancreatic islets of *mir-7$^{fl/fl}$* and *Sim1-cre;mir-7$^{fl/fl}$* mice (n = 5 animals).

Data are presented as mean ± SD. *P < 0.05; **P < 0.01; no asterisk indicates P > 0.05; 2-way repeated measures ANOVA with Sidak's multiple comparisons test (a, b, d, f, h, j, l, n, p, r, t, w, x, z, aa), 2-way repeated measures ANOVA (c, e, g, i, k, m, o, q, s, v, y), or two-tailed *t* test (u, ac). Source data are provided as a Source Data file.

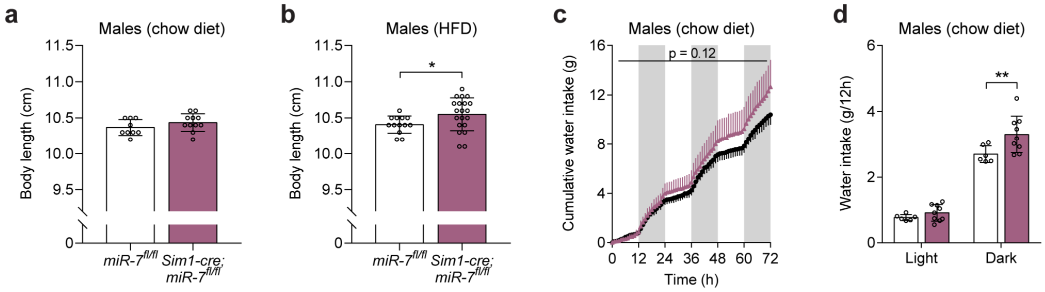

Supplementary Figure 3. Neuroendocrine phenotypes of *Sim1-cre;mir-7$^{fl/fl}$* mice.

(a–b) Body length of chow-fed male (n = 9 and 11 animals) (a) and HFD-fed male (n = 13 and 20 animals) (b) *mir-7$^{fl/fl}$* and *Sim1-cre;mir-7$^{fl/fl}$* mice.

(c–d) 72-hour metabolic cage measurements of chow-fed male *mir-7$^{fl/fl}$* and *Sim1-cre;mir-7$^{fl/fl}$* mice (n = 6 and 9 animals). (c) cumulative water intake; (d) average water intake per 12-hour phase. Dark phases are shaded in grey.

Data are presented as mean ± SD. *P < 0.05; **P < 0.01; no asterisk indicates P > 0.05; two-tailed *t* test (a, b), 2-way repeated measures ANOVA (c), or 2-way repeated measures ANOVA with Sidak's multiple comparisons test (d). Source data are provided as a Source Data file.

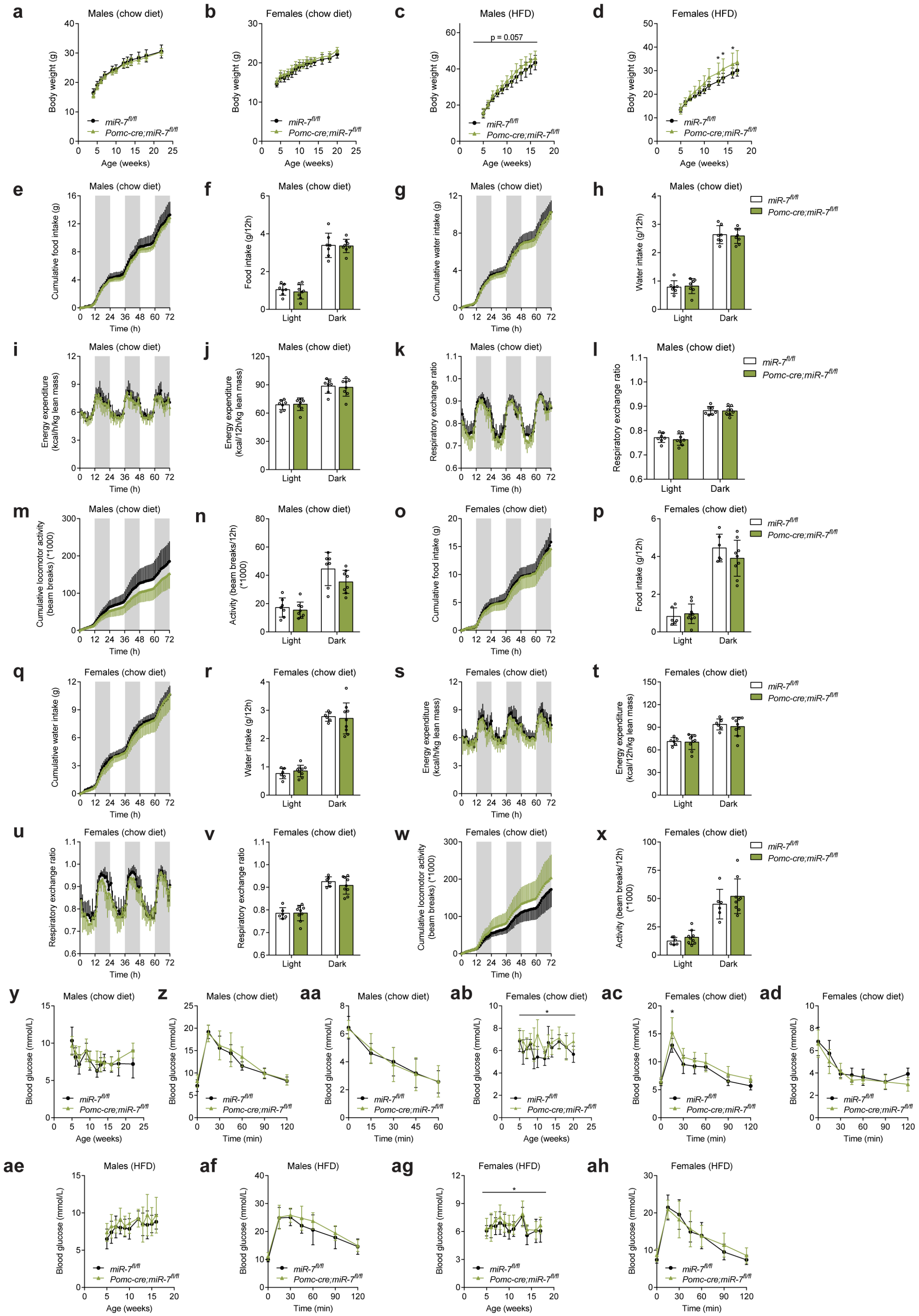

Supplementary Figure 4. Metabolic phenotypes of *Pomc-cre;mir-7$^{fl/fl}$* mice. ***(continued on next page)***

Supplementary Figure 4. Metabolic phenotypes of *Pomc-cre;mir-7*$^{fl/fl}$ mice *(continued)*.

(a–d) Body weight curves of chow-fed male (n = 7 and 8 animals) (a), chow-fed female (n = 6 and 9 animals) (b), HFD-fed male (n = 14 and 13 animals) (c), and HFD-fed female (n = 9 and 15 animals) (d) *mir-7*$^{fl/fl}$ and *Pomc-cre;mir-7*$^{fl/fl}$ mice.

(e–x) 72-hour metabolic cage measurements of chow-fed male (n= 7 and 8 animals) (e-n) and chow-fed female (n = 6 and 9 animals) (o-x) *mir-7*$^{fl/fl}$ and *Pomc-cre;mir-7*$^{fl/fl}$ mice. (e, o) Cumulative food intake; (f, p) average food intake per 12-hour phase; (g, q) cumulative water intake; (h, r) average water intake per 12-hour phase; (i, s) hourly energy expenditure; (j, t) average energy expenditure per 12-hour phase; (k, u) hourly RER; (l, v) average RER per 12-hour phase; (m, w) cumulative locomotor activity; (n, x) average locomotor activity per 12-hour phase. Dark phases are shaded in grey.

(y–aa) Random fed blood glucose levels (n = 7 and 8 animals) (y), blood glucose levels during an ipGTT (n = 7 and 8 animals) (z), and blood glucose levels during an ipITT (n = 5 and 7 animals) (aa) in chow-fed male *mir-7*$^{fl/fl}$ and *Pomc-cre;mir-7*$^{fl/fl}$ mice.

(ab–ad) Random fed blood glucose levels (n = 6 and 9 animals) (ab), blood glucose levels during an ipGTT (n = 6 and 9 animals) (ac), and blood glucose levels during an ipITT (n = 7 and 6 animals) (ad) in chow-fed female *mir-7*$^{fl/fl}$ and *Pomc-cre;mir-7*$^{fl/fl}$ mice.

(ae–af) Random fed blood glucose levels (n = 14 and 13 animals) (ae) and blood glucose levels during an ipGTT (n = 7 animals) (af) in HFD-fed male *mir-7*$^{fl/fl}$ and *Pomc-cre;mir-7*$^{fl/fl}$ mice.

(ag–ah) Random fed blood glucose levels (n = 9 and 15 animals) (ac) and blood glucose levels during an ipGTT (n = 8 and 10 animals) (ad) in HFD-fed female *mir-7*$^{fl/fl}$ and *Pomc-cre;mir-7*$^{fl/fl}$ mice.

Data are presented as mean ± SD. *P < 0.05; no asterisk indicates P > 0.05; 2-way repeated measures ANOVA with Sidak's multiple comparisons test (a, b, c, d, f, h, j, l, n, p, r, t, v, x, z, aa, ac, ad, af, ah), or 2-way repeated measures ANOVA (e, g, i, k, m, o, q, s, u, w, y, ab, ae, ag). Source data are provided as a Source Data file.

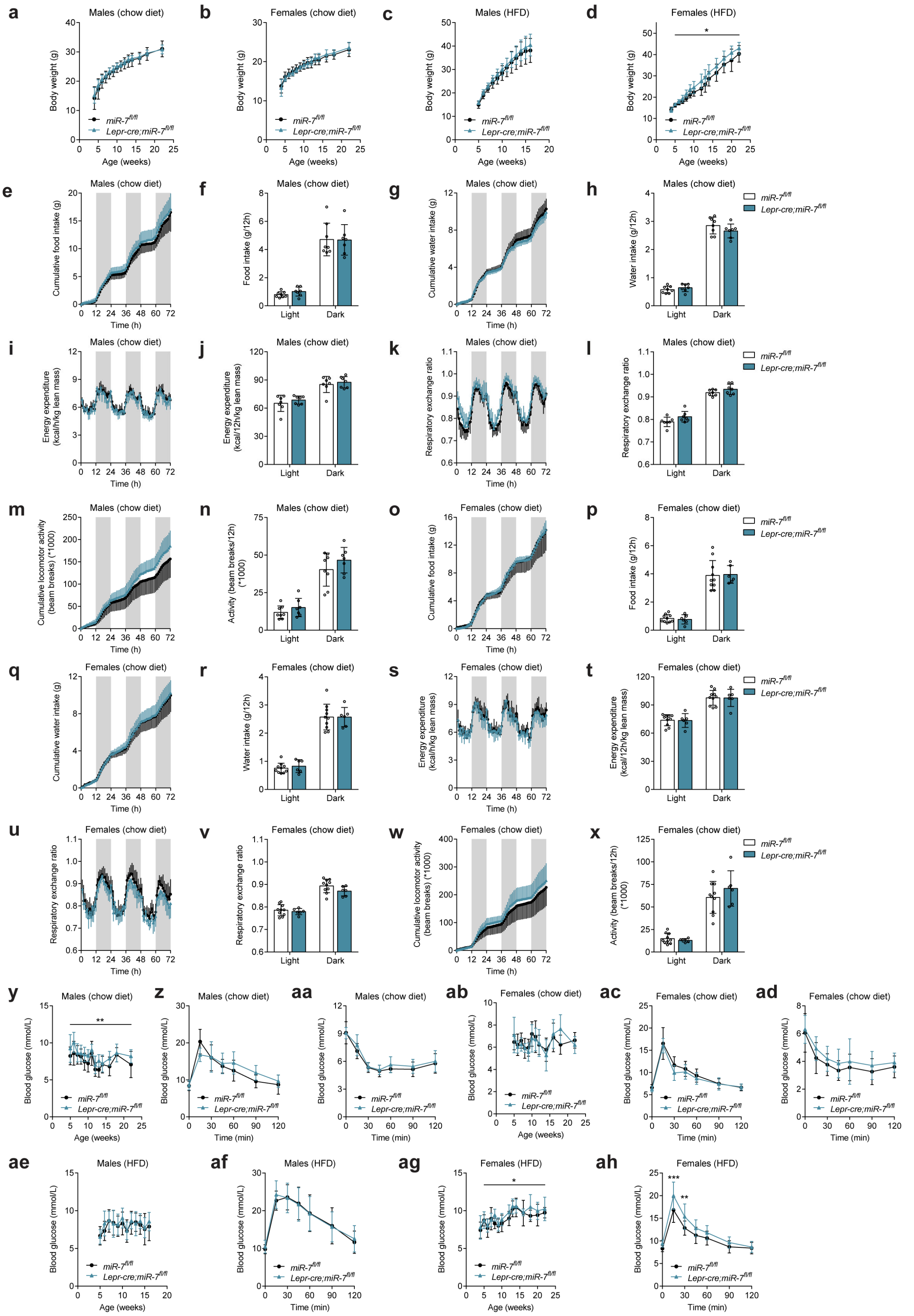

Supplementary Figure 5. Metabolic phenotypes of *Lepr-cre;mir-7^fl/fl^* mice. ***(continued on next page)***

Supplementary Figure 5. Metabolic phenotypes of *Lepr-cre;mir-7$^{fl/fl}$* mice *(continued)*.

(a–d) Body weight curves of chow-fed male (n = 8 and 7 animals) (a), chow-fed female (n = 13 and 6 animals) (b), HFD-fed male (n = 14 and 10 animals) (c), and HFD-fed female (n = 11 animals) (d) *mir-7$^{fl/fl}$* and *Lepr-cre;mir-7$^{fl/fl}$* mice.

(e–x) 72-hour metabolic cage measurements of chow-fed male (n = 7 and 9 animals) (e-n) and chow-fed female (n = 10 and 6 animals) (o-x) *mir-7$^{fl/fl}$* and *Lepr-cre;mir-7$^{fl/fl}$* mice. (e, o) Cumulative food intake; (f, p) average food intake per 12-hour phase; (g, q) cumulative water intake; (h, r) average water intake per 12-hour phase; (i, s) hourly energy expenditure; (j, t) average energy expenditure per 12-hour phase; (k, u) hourly RER; (l, v) average RER per 12-hour phase; (m, w) cumulative locomotor activity; (n, x) average locomotor activity per 12-hour phase. Dark phases are shaded in grey.

(y–aa) Random fed blood glucose levels (n = 8 and 7 animals) (y), blood glucose levels during an ipGTT (n = 8 and 6 animals) (z), and blood glucose levels during an ipITT (n = 10 and 9 animals) (aa) in chow-fed male *mir-7$^{fl/fl}$* and *Lepr-cre;mir-7$^{fl/fl}$* mice.

(ab–ad) Random fed blood glucose levels (n = 13 and 6 animals) (ab), blood glucose levels during an ipGTT (n = 13 and 6 animals) (ac), and blood glucose levels during an ipITT (n = 10 and 7 animals) (ad) in chow-fed female *mir-7$^{fl/fl}$* and *Lepr-cre;mir-7$^{fl/fl}$* mice.

(ae–af) Random fed blood glucose levels (n = 14 and 10 animals) (ae) and blood glucose levels during an ipGTT (n = 14 and 10 animals) (af) in HFD-fed male *mir-7$^{fl/fl}$* and *Lepr-cre;mir-7$^{fl/fl}$* mice.

(ag–ah) Random fed blood glucose levels (n = 11 animals) (ac) and blood glucose levels during an ipGTT (n = 11 animals) (ad) in HFD-fed female *mir-7$^{fl/fl}$* and *Lepr-cre;mir-7$^{fl/fl}$* mice.

Data are presented as mean ± SD. *P < 0.05; **P < 0.01; ***P < 0.001; no asterisk indicates P > 0.05; 2-way repeated measures ANOVA with Sidak's multiple comparisons test (a, b, c, d, f, h, j, l, n, p, r, t, v, x, z, aa, ac, ad, af, ah), or 2-way repeated measures ANOVA (e, g, i, k, m, o, q, s, u, w, y, ab, ae, ag). Source data are provided as a Source Data file.

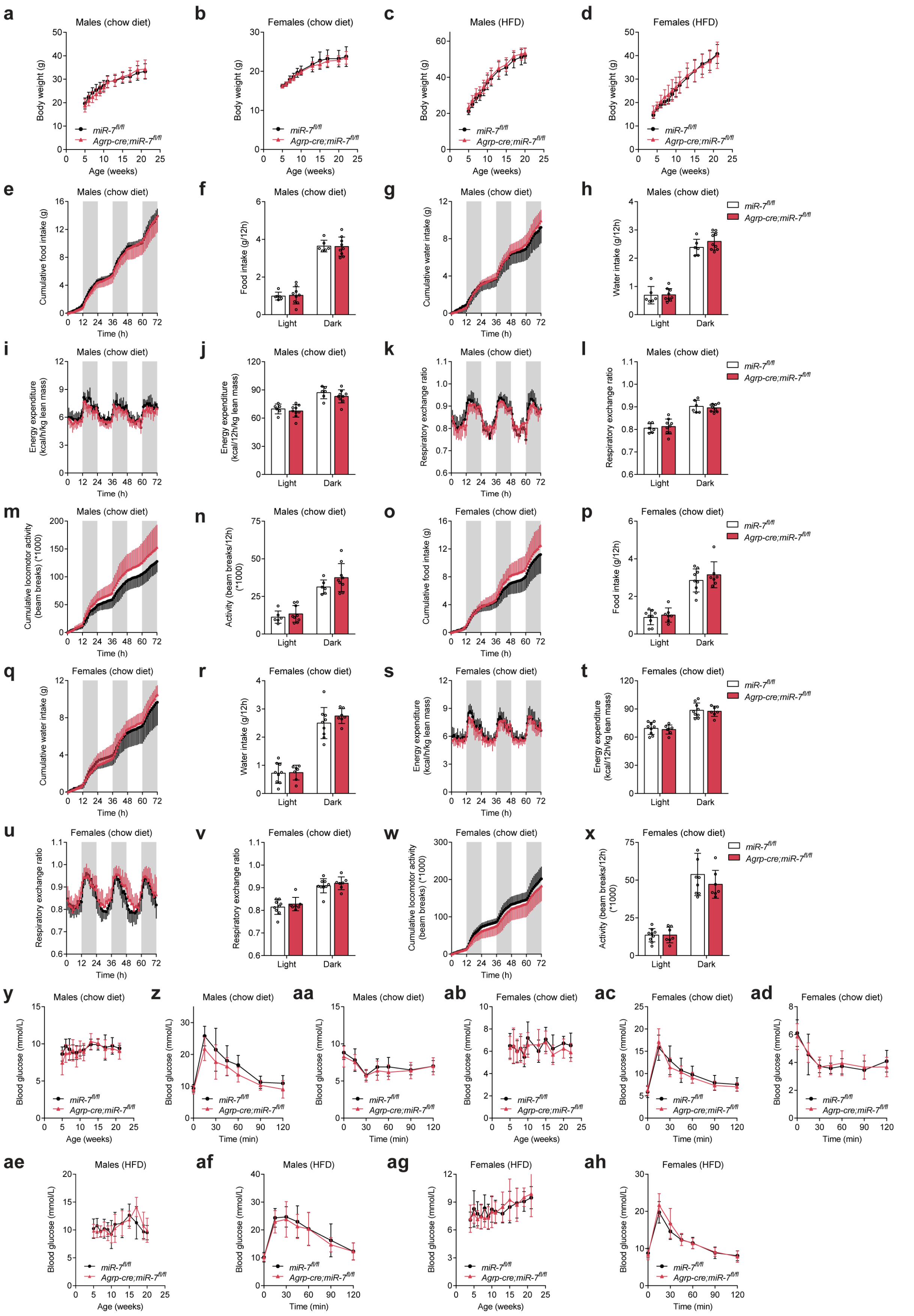

Supplementary Figure 6. Metabolic phenotypes of *Agrp-cre;mir-7*$^{fl/fl}$ mice. ***(continued on next page)***

Supplementary Figure 6. Metabolic phenotypes of *Agrp-cre;mir-7*$^{fl/fl}$ mice *(continued)*.

(a–d) Body weight curves of chow-fed male (n = 6 and 9 animals) (a), chow-fed female (n = 9 and 7 animals) (b), HFD-fed male (n = 11 and 6 animals) (c), and HFD-fed female (n = 16 and 7 animals) (d) *mir-7*$^{fl/fl}$ and *Agrp-cre;mir-7*$^{fl/fl}$ mice.

(e–x) 72-hour metabolic cage measurements of chow-fed male (n = 6 and 9 animals) (e-n) and chow-fed female (n = 9 and 7 animals) (o-x) *mir-7*$^{fl/fl}$ and *Agrp-cre;mir-7*$^{fl/fl}$ mice. (e, o) Cumulative food intake; (f, p) average food intake per 12-hour phase; (g, q) cumulative water intake; (h, r) average water intake per 12-hour phase; (i, s) hourly energy expenditure; (j, t) average energy expenditure per 12-hour phase; (k, u) hourly RER; (l, v) average RER per 12-hour phase; (m, w) cumulative locomotor activity; (n, x) average locomotor activity per 12-hour phase. Dark phases are shaded in grey.

(y–aa) Random fed blood glucose levels (n = 6 and 9 animals) (y), blood glucose levels during an ipGTT (n = 6 and 9 animals) (z), and blood glucose levels during an ipITT (n = 6 and 9 animals) (aa) in chow-fed male *mir-7*$^{fl/fl}$ and *Agrp-cre;mir-7*$^{fl/fl}$ mice.

(ab–ad) Random fed blood glucose levels (n = 9 and 7 animals) (ab), blood glucose levels during an ipGTT (n = 11 and 7 animals) (ac), and blood glucose levels during an ipITT (n = 11 and 7 animals) (ad) in chow-fed female *mir-7*$^{fl/fl}$ and *Agrp-cre;mir-7*$^{fl/fl}$ mice.

(ae–af) Random fed blood glucose levels (n = 11 and 6 animals) (ae) and blood glucose levels during an ipGTT (n = 11 and 6 animals) (af) in HFD-fed male *mir-7*$^{fl/fl}$ and *Agrp-cre;mir-7*$^{fl/fl}$ mice.

(ag–ah) Random fed blood glucose levels (n = 16 and 7 animals) (ac) and blood glucose levels during an ipGTT(n = 16 and 7 animals) (ad) in HFD-fed female *mir-7*$^{fl/fl}$ and *Agrp-cre;mir-7*$^{fl/fl}$ mice.

Data are presented as mean ± SD. No asterisk indicates P > 0.05; 2-way repeated measures ANOVA with Sidak's multiple comparisons test (a, b, c, d, f, h, j, l, n, p, r, t, v, x, z, aa, ac, ad, af, ah), or 2-way repeated measures ANOVA (e, g, i, k, m, o, q, s, u, w, y, ab, ae, ag). Source data are provided as a Source Data file.

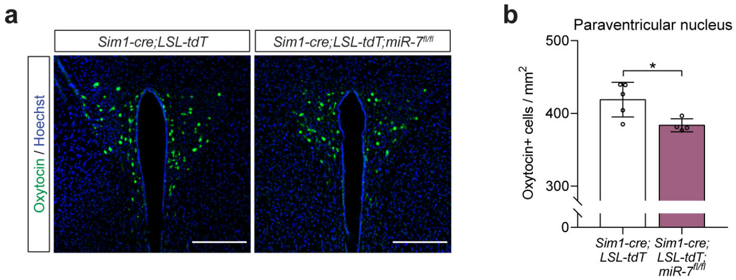

Supplementary Figure 7. Characterization of Sim1 neuron defects.

(a–b) Representative image (scale bar: 250 μm) (a) and quantification (b) of oxytocin-positive cell number within the PVN of *Sim1-cre;LSL-tdTomato* and *Sim1-cre;LSL-tdTomato;mir-7*$^{fl/fl}$ mice (n = 5 and 4 animals). Green, oxytocin; blue, Hoechst.

Data are presented as mean ± SD. *P < 0.05; two-tailed *t* test (b). Source data are provided as a Source Data file.

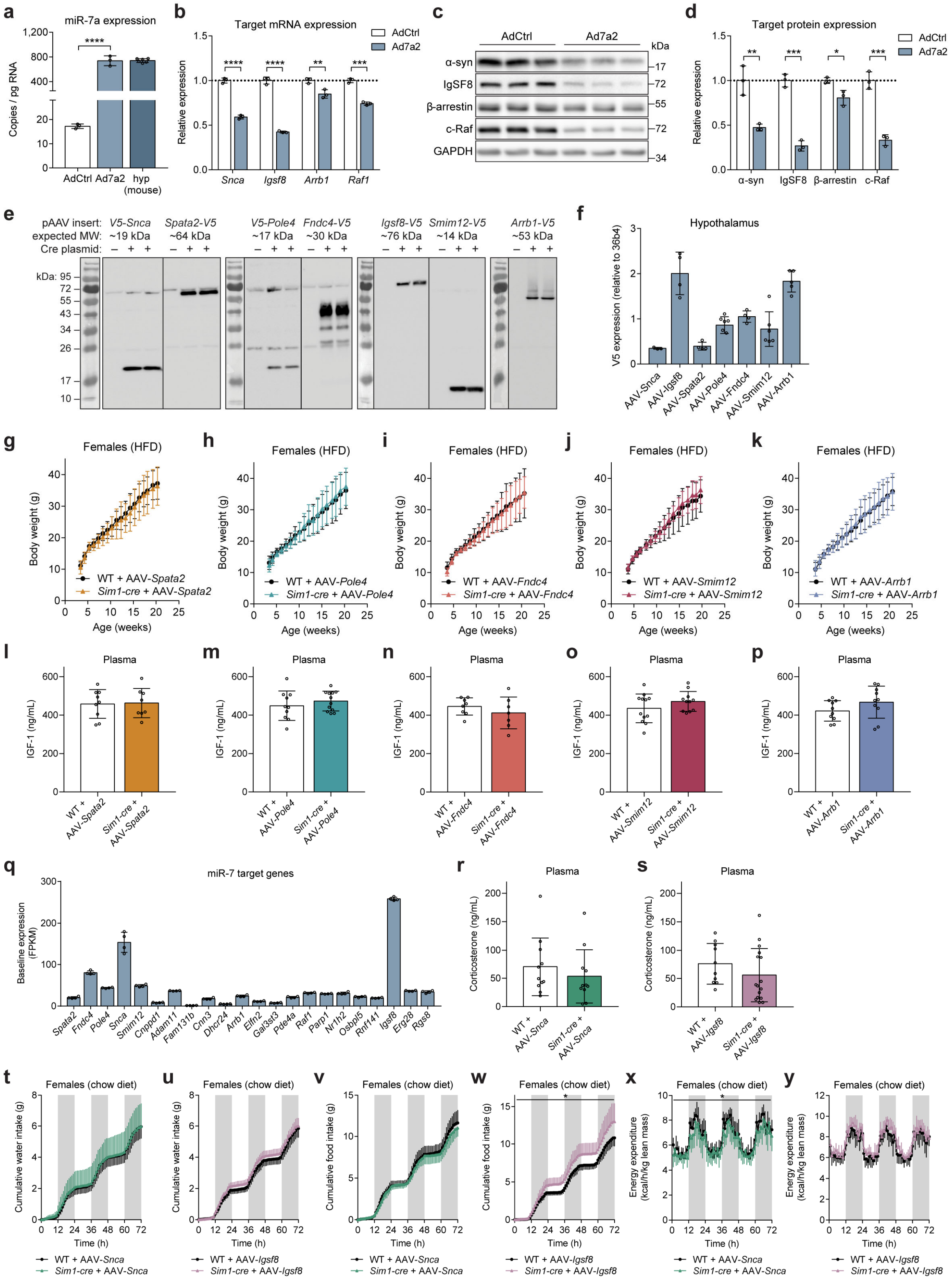

Supplementary Figure 8. Characterization of target regulation by miR-7 and AAV-mediated target gene overexpression. ***(continued on next page)***

Supplementary Figure 8. Characterization of target regulation by miR-7 and AAV-mediated target gene overexpression *(continued).*

(a–d) Analyses of SH-SY5Y cells transfected with AdCtrl or Ad7a2 (n = 3; representative of two independent experiments). (a) miR-7a expression in SH-SY5Y cells compared to mouse hypothalamus; (b) mRNA expression of miR-7 targets; (c) Western blot of miR-7 targets; (d) quantification of Western blot shown in (c).

(e) Western blot of V5 in cells transfected with pAAV-FLEx-target gene plasmids, with or without co-expression of a plasmid encoding Cre recombinase. Each lane represents one well of transfected cells, obtained from one independent experiment. Expected molecular weight (MW) was determined by the amino acid sequence.

(f) V5 expression (relative to 36b4) in the hypothalamus of *Sim1-cre* mice, 4 months after injection of AAV-*Snca* (n = 3 animals), AAV-*Igsf8* (n = 4 animals), AAV-*Spata2* (n = 4 animals), AAV-*Pole4* (n = 7 animals), AAV-*Fndc4* (n = 4 animals), AAV-*Smim12* (n = 6 animals), or AAV-*Arrb1* (n = 5 animals).

(g–k) Body weight curves of HFD-fed female WT and *Sim1-cre* mice injected with AAV-*Spata2* (n = 9 and 8 animals) (g), AAV-*Pole4* (n = 10 and 12 animals) (h), AAV-*Fndc4* (n = 8 and 7 animals) (i), AAV-*Smim12* (n = 12 and 11 animals) (j), or AAV-*Arrb1* (n = 10 animals) (k).

(l–p) Plasma IGF-1 levels in female WT and *Sim1-cre* mice injected with AAV-*Spata2* (n = 9 and 8 animals) (l), AAV-*Pole4* (n = 10 and 12 animals) (m), AAV-*Fndc4* (n = 8 and 7 animals) (n), AAV-*Smim12* (n = 12 and 11 animals) (o), or AAV-*Arrb1* (n = 10 animals) (p).

(q) Baseline expression (*i.e.* expression in *mir-7$^{fl/fl}$* mice) of the 22 upregulated miR-7 target genes identified in Fig. 5c,d (n = 4 samples, with 4 mice pooled per sample). FPKM, Fragments Per Kilobase of transcript per Million.

(r–s) Plasma corticosterone levels in WT and *Sim1-cre* mice injected with AAV-*Snca* (n = 11 animals) (r) or AAV-*Igsf8* (n = 10 and 16 animals) (s).

(t–y) 72-hour metabolic cage measurements of WT and *Sim1-cre* mice injected with AAV-*Snca* (n = 8 animals) (t, v, x) or AAV-*Igsf8* (n = 8 animals) (u, w, y). (t, u) cumulative water intake; (v, w) cumulative food intake; (x, y) hourly energy expenditure. Dark phases are shaded in grey.

Data are presented as mean ± SD. *P < 0.05; **P < 0.01; ***P < 0.001; ****P < 0.0001; no asterisk indicates P > 0.05; 1-way ANOVA with Tukey's multiple comparisons test (a), two-tailed *t* test (b, d, l, m, n, o, p, r, s), or 2-way repeated measures ANOVA (g–k, t–y; genotype-by-time interaction in v, w). Source data are provided as a Source Data file.

SUPPLEMENTARY TABLES

Supplementary Table 1. Primers used for qPCR

Species	Gene	Forward primer	Reverse primer
Mouse	*36b4*	GCCGTGATGCCCAGGGAAGACA	CATCTGCTTGGAGCCCACGTTG
Mouse	*Adrb3*	CCTTCAACCCGGTCATCTAC	GAAGATGGGGATCAAGCAAGC
Mouse	*Cdr1as*	CATTCAGGTTTTCTGGTGTCTGC	GACCTGGAGATGTTGGAAGACC
Mouse	*Cyrano*	TAGCATGTGAGACTGGGTTGG	AGGGTCCACCACATCACCTT
Mouse	*Gh*	GCTTGGCAATGGCTACAGA	GGAAAAGCACTAGCCTCCTG
Mouse	*Pgc1a*	GAAAGGGCCAAACAGAGAGA	GTAAATCACACGGCGCTCTT
Mouse	*Pomc*	CCATAGATGTGTGGAGCTGGT	AGCGAGAGGTCGAGTTTGC
Mouse	*Ucp1*	CGACTCAGTCCAAGAGTACTTCT CTT	GCCGGCTGAGATCTTGTTTC
Human	*Snca*	TCGACGACAGTGTGGTGTAA	TCCCTCCTTGGTTTTGGAGC
Human	*Igsf8*	GCTTCCTGCCCTCAACCTG	GGGCCCAGCATCCCATTC
Human	*Arrb1*	CAAAGGGACCCGAGTGTTCA	GCAGGTCAGCGTCACATAGA
Human	*Raf1*	AAAGCACTCAAGGTGAGGGG	AGCTTCAGGAACGTCTTCCG
Human	*Rplp0*	CGTCCTCGTGGAAGTGACAT	ATCTGCTTGGAGCCCACATT
	V5 tag	GGTAAGCCTATCCCTAAC	CGTAGAATCGAGACCGAG

Supplementary Table 2. Sequences of synthesized Igsf8 3'UTR fragments

Igsf8 3'UTR-WT	GAGCTCTCTGTAGCACCCCAGTTCTTGCAGGTGTTGACTGTCTT CCAGCCAGTGCCACCCCTCCTCTGGTTGCCTGGATACCCTCTCC CTCTGTCCAGCCCACCTTTAATTTATTTGACCAGCCATCACCTA GAGTGGGAGACATGGCTCCCCTGTTCCCTCCTTCTCTAGA
Igsf8 3'UTR-Mut	GAGCTCTCTGTAGCACCCCAGTTCTTGCAGGTGTTGACTGCCCT TCAGCCAGTGCCACCCCTCCTCTGGTTGCCTGGATACCCTCTCC CTCTGTCCAGCCCACCTTTAATTTATTTGACCAGCCATCACCTA GAGTGGGAGACATGGCTCCCCTGTTCCCTCCTTCTCTAGA

Supplementary Table 3. Oligos and primers used for generation of pAAV-FLEx-target gene vectors

N-V5-MCS_fw annealing oligo	CGCCACCATGGGTAAGCCTATCCCTAACCCTCTCCTCGGTCTC GATTCTACGACTAGTACGCGTATGCATC
N-V5-MCS_rev annealing oligo	TCGAGATGCATACGCGTACTAGTCGTAGAATCGAGACCGAGG AGAGGGTTAGGGATAGGCTTACCCATGGTGGCGGTAC
C-V5-MCS_fw annealing oligo	CACTAGTACGCGTATGCATGGTAAGCCTATCCCTAACCCTCTC CTCGGTCTCGATTCTACGTAAC
C-V5-MCS_rev annealing oligo	TCGAGTTACGTAGAATCGAGACCGAGGAGAGGGTTAGGGATA GGCTTACCATGCATACGCGTACTAGTGGTAC
Snca_N-V5_fw	AAAAACTAGTGGTGGTGGTGGTTCTGATGTGTTCATGAAAGG
Snca_N-V5_rev	CGCCGCATGCATTTAGGCTTCAGGCTCATAG
Igsf8_C-V5_fw	AAAAACTAGTCCGCCACCATGGGCGTCCCTAGCC
Igsf8_C-V5_rev	CGCCGCATGCATAGAACCACCACCACCCCGCTTCCGCATCCTC
Spata2_C-V5_fw	AAAAACTAGTCCGCCACCATGGATACGAAGTACAAGG
Spata2_C-V5_ref	CGCCGCATGCATAGAACCACCACCACCTCTGTACACGAGCTG GG
Pole4_N-V5_fw	AAAAACTAGTGGTGGTGGTGGTTCTGCGGCAGCGGCGGCT
Pole4_N-V5_rev	CGCCGCATGCATTTAATCCAAAGTGCCTTCTAG
Fndc4_C-V5_fw	AAAAACTAGTCCGCCACCATGCCTCTGGCCCCTC
Fndc4_C-V5_rev	CGCCGCATGCATAGAACCACCACCACCAACATCAATGGTGTT GATTGATGG
Smim12_C-V5_fw	AAAAACTAGTCCGCCACCATGTGGCCTGTGCTTTG
Smim12_C-V5_rev	CGCCGCATGCATAGAACCACCACCACCGTTCTTCTCTGGACG GTTTC
Arrb1_C-V5_fw	AAAAACTAGTCCGCCACCATGGGCGACAAAGGGAC
Arrb1_C-V5_rev	CGCCGCATGCATAGAACCACCACCACCTCTGTTGTTGAGGTG CG
