# Supplementary material for: MicroRNA-7 regulates melanocortin circuits involved in mammalian energy homeostasis
Source: Nat Commun. 2022 Sep 29;13:5733. doi: 10.1038/s41467-022-33367-w (PMC9522793; doi:10.1038/s41467-022-33367-w)
Supplement: Supplementary file 3 — Description of Additional Supplementary Files [file 41467_2022_33367_MOESM3_ESM.pdf]

Description of Additional Supplementary Files

File Name: Supplementary Data 1
Description: **Locus-to-gene scores for loci associated with HNRNPK by the OpenTargets L2G pipeline (Version 5).**
Data from Open Targets Genetics, https://genetics.opentargets.org/ (Version 5).
(**a**) All loci with an L2G score for HNRNPK.
(**b**) Selected traits only, grouped by traits.
(**c**) Selected traits only, ordered by L2G score for HNRNPK.

File Name: Supplementary Data 2
Description: **PhenomeXcan and PhenomeXcan_SingleTissue results for HNRNPK, IGSF8 and SNCA.**
(**a–b**) PhenomeXcan gene search for HNRNPK, (a) RCP > 0.1, (b) RCP>0.01 (RCP, Regional Colocalization Probability).
(**c–e**) PhenomeXcan Single Tissue gene search for HNRNPK with trait Standing height (c), Impedance of whole body (d), Trunk fat percentage (e).
(**f–g**) PhenomeXcan gene search for IGSF8, (f) RCP > 0.1, (g) RCP>0.01.
(**h**) PhenomeXcan Single Tissue gene search for IGSF8 with trait Leg fat percentage (right).
(**i–j)** PhenomeXcan gene search for SNCA, (i) RCP > 0.1, (j) RCP>0.01.
(**k–l**) PhenomeXcan Single Tissue gene search for SNCA with trait Comparative height size at age 10 (k), Standing height (l).
(**m**) Variant-level GWAS-eQTL colocalisation as reported by PhenomeXcan's fastENLOC step, displayed for the gene-trait pairs and GTEx tissues highlighted in Table S2 tissue-level tables. Link-out URLs are provided to Open Targets Genetics variant-level browser (to facilitate variant-level browsing across other genetic datasets).
(**n**) Variants comprising the expression prediction models of PhenomeXcan's PrediXcan steps (to illustrate how many and which variants are used to predict genetically-regulated gene expression of HNRNPK, IGSF8 and SNCA) and URLs to Open Targets Genetics variant-level browser (to facilitate variant-level browsing across other genetic datasets).
Rows referring to pre-selected traits (PhenomeXcan consensus results across tissues) or neuro/endocrine tissues and cell types (PhenomeXcan_SingleTissue tables) are highlighted by direction of effect, blue (negative) or red (positive).
Column descriptions for PhenomeXcan tables (adapted from https://phenomexcan.org) are phenotype_source: 'UK Biobank' or other cohorts; rcp: Regional Colocalization Probability (fastENLOC/ENLOC); pvalue: p-value from S-MultiXcan; best_sign: sign of effect of the most significant tissue ['+' ('-') means higher (lower) expression is associated with higher risk or higher value of phenotype]; consensus_sign: sign of effect of the consensus among those tissues with pvalue < 1e-4 ['+' and '-' as for best_sign column]; n_tissues: number of tissues available to S-MultiXcan when computing significance for a gene; n_indep: number of independent components of variations among n_tissues.
Column descriptions for PhenomeXcan_SingleTissue tables are P value: p-value from S-PrediXcan; zscore: Z-score from S-PrediXcan; effect_size: effect size from S-PrediXcan.
PIP: posterior inclusion probability.
