# Supplementary material for: MicroRNA-7 regulates melanocortin circuits involved in mammalian energy homeostasis
Source: Nat Commun. 2022 Sep 29;13:5733. doi: 10.1038/s41467-022-33367-w (PMC9522793; doi:10.1038/s41467-022-33367-w)
Supplement: Supplementary file 6 — Reporting Summary [file 41467_2022_33367_MOESM6_ESM.pdf]

nature portfolio

Corresponding author(s): Markus Stoffel

Last updated by author(s): Aug 19, 2022

Reporting Summary

Nature Portfolio wishes to improve the reproducibility of the work that we publish. This form provides structure for consistency and transparency in reporting. For further information on Nature Portfolio policies, see our Editorial Policies and the Editorial Policy Checklist.

Statistics

For all statistical analyses, confirm that the following items are present in the figure legend, table legend, main text, or Methods section.

n/a	Confirmed	
☐	☒	The exact sample size (*n*) for each experimental group/condition, given as a discrete number and unit of measurement
☐	☒	A statement on whether measurements were taken from distinct samples or whether the same sample was measured repeatedly
☐	☒	The statistical test(s) used AND whether they are one- or two-sided<br>*Only common tests should be described solely by name; describe more complex techniques in the Methods section.*
☐	☒	A description of all covariates tested
☐	☒	A description of any assumptions or corrections, such as tests of normality and adjustment for multiple comparisons
☐	☒	A full description of the statistical parameters including central tendency (e.g. means) or other basic estimates (e.g. regression coefficient) AND variation (e.g. standard deviation) or associated estimates of uncertainty (e.g. confidence intervals)
☐	☒	For null hypothesis testing, the test statistic (e.g. *F*, *t*, *r*) with confidence intervals, effect sizes, degrees of freedom and *P* value noted<br>*Give P values as exact values whenever suitable.*
☒	☐	For Bayesian analysis, information on the choice of priors and Markov chain Monte Carlo settings
☒	☐	For hierarchical and complex designs, identification of the appropriate level for tests and full reporting of outcomes
☒	☐	Estimates of effect sizes (e.g. Cohen's *d*, Pearson's *r*), indicating how they were calculated

Our web collection on statistics for biologists contains articles on many of the points above.

Software and code

Policy information about availability of computer code

Data collection	No specific software was used
Data analysis	For RNA sequencing analysis, raw reads were cleaned using Trimmomatic (Version 0.36). Sequence pseudo alignment of the resulting high-quality reads to the mouse reference genome and quantification of gene level expression were carried out using Kallisto (Version 0.44).<br>Differentially expressed genes were identified using the R package edgeR from Bioconductor Version 20.<br>Image quantification was performed using QuPath software version 0.1.2.<br>Western Blot densitometry was performed using ImageJ version 1.53c.<br>Statistical analysis was performed using GraphPad Prism version 7.

For manuscripts utilizing custom algorithms or software that are central to the research but not yet described in published literature, software must be made available to editors and reviewers. We strongly encourage code deposition in a community repository (e.g. GitHub). See the Nature Portfolio guidelines for submitting code & software for further information.

Data

Policy information about availability of data

All manuscripts must include a data availability statement. This statement should provide the following information, where applicable:

- Accession codes, unique identifiers, or web links for publicly available datasets
- A description of any restrictions on data availability
- For clinical datasets or third party data, please ensure that the statement adheres to our policy

RNA sequencing data have been deposited to the European Nucleotide Archive (accession ID PRJEB48660). Source data are provided with this paper. The following third-party data sources were used in this study: mouse reference genome GRCm38.p6 assembly (https://www.ncbi.nlm.nih.gov/assembly/GCF_000001635.26/)

Open Targets Genetics (https://genetics.opentargets.org), FIVEx (https://fivex.sph.umich.edu), PhenomeXcan (http://phenomexcan.org), PhenomeXcan fastENLOC results (https://github.com/hakyimlab/phenomexcan), PrediXcan GTEx v8 expression predictors (https://www.predictdb.org), GTEx v8 eQTLs (accessible via https://www.gtexportal.org/home/datasets).

Field-specific reporting

Please select the one below that is the best fit for your research. If you are not sure, read the appropriate sections before making your selection.

☒ Life sciences ☐ Behavioural & social sciences ☐ Ecological, evolutionary & environmental sciences

For a reference copy of the document with all sections, see nature.com/documents/nr-reporting-summary-flat.pdf

Life sciences study design

All studies must disclose on these points even when the disclosure is negative.

Sample size	No statistical methods were used to calculate sample size. Sample sizes were determined based on previously published data using similar methods (e.g. Xi et al, 2012 (doi: 10.1371/journal.pone.0036453); Latreille et al, 2014 (doi: 10.1172/JCI73066)).
Data exclusions	No data were excluded from analyses.
Replication	In all animal experiments, one sample (n) represents one animal (except RNA sequencing, in which four mice were pooled per sample). For immunostaining, three brain sections per mouse were analysed and reported as the average per mouse. In cell culture experiments, one sample (n) represents one well of a multi-well plate. In Western blots, samples were assayed in unicates and quantifications are representative of at least 2 experiments. In qPCR and hormone measurements, samples were assayed in duplicates and reported as the average per sample. All attempts at replication were successful.
Randomization	For most animal experiments, the breeding strategy was such that all mice in a litter were either controls or transgenic/knockouts; therefore, all mice in a litter were used for experiments. Multiple litters were combined for each cohort to achieve sufficient sample sizes. For experiments that could only include a portion of the cohort for technical reasons (e.g. metabolic cages) or for allocation to experimental groups, mice were randomised in a manner that distributed littermates evenly across groups, and to ensure that their average body weight matched that of their respective group.
Blinding	Investigators were blinded to group allocation during data collection, sample collection/processing, and analysis.

Reporting for specific materials, systems and methods

We require information from authors about some types of materials, experimental systems and methods used in many studies. Here, indicate whether each material, system or method listed is relevant to your study. If you are not sure if a list item applies to your research, read the appropriate section before selecting a response.

Materials & experimental systems

n/a	Involved in the study	
☐	☒	Antibodies
☐	☒	Eukaryotic cell lines
☒	☐	Palaeontology and archaeology
☐	☒	Animals and other organisms
☒	☐	Human research participants
☒	☐	Clinical data
☒	☐	Dual use research of concern

Methods

n/a	Involved in the study	
☒	☐	ChIP-seq
☒	☐	Flow cytometry
☒	☐	MRI-based neuroimaging

Antibodies

Antibodies used	guinea pig anti-insulin (A056401, Dako), rabbit anti-phospho-CREB (Ser133) (87G3) (9198, Cell Signaling), rabbit anti-Oxytocin (T-4084, Peninsula Laboratories), mouse anti-V5 tag (R960-25, Invitrogen), goat anti-IgSF8 (AF3117, R&D Systems), mouse anti-Alpha-synuclein (42) (610786, BD Biosciences), rabbit anti-Beta-arrestin (E274) (ab32099, Abcam), rabbit anti-c-Raf (D4B3J) (53745, Cell Signalling), rabbit anti-GAPDH (14C10) (2118, Cell Signalling), goat anti-guinea pig conjugated to Alexa Fluor 488 (A11073, Invitrogen), goat anti-rabbit conjugated to Cy5 (A10523, Invitrogen), goat anti-mouse conjugated to Alexa647 (115-605-003, Jackson ImmunoResearch), goat anti-mouse conjugated to HRP (401253 Sigma), goat anti-rabbit conjugated to HRP (401393, Sigma), and rabbit anti-goat conjugated to HRP (401515 Sigma).
Validation	Antibody validation is available through manufacturer's websites.<br>guinea pig anti-insulin (A056401, Dako): https://www.agilent.com/store/en_US/Prod-A056401-2/A056401-2<br>rabbit anti-phospho-CREB (Ser133) (9198, Cell Signaling): https://www.cellsignal.com/products/primary-antibodies/phospho-creb-ser133-87g3-rabbit-mab/9198<br>rabbit anti-Oxytocin (T-4084, Peninsula Laboratories): http://www.bma.ch/en/products/T-4084

mouse anti-V5 tag (R960-25, Invitrogen): https://www.thermofisher.com/antibody/product/V5-Tag-Antibody-Monoclonal/R960-25
goat anti-IgSF8 (AF3117, R&D Systems): https://www.rndsystems.com/products/human-mouse-rat-igsf8-cd316-antibody_af3117#product-citations
mouse anti alpha-synuclein (610786, BD Biosciences): https://www.bdbiosciences.com/en-au/products/reagents/microscopy-imaging-reagents/immunofluorescence-reagents/purified-mouse-anti-synuclein.610786
rabbit anti-Beta-arrestin (ab32099, Abcam): https://www.abcam.com/nav/primary-antibodies/rabbit-monoclonal-antibodies/beta-arrestin-1-antibody-e274-ab32099.html
rabbit anti-c-Raf (53745, Cell Signalling): https://www.cellsignal.com/products/primary-antibodies/c-raf-d4b3j-rabbit-mab/53745
rabbit anti-GAPDH (2118, Cell Signalling): https://www.cellsignal.com/products/primary-antibodies/gapdh-14c10-rabbit-mab/2118

Eukaryotic cell lines

Policy information about cell lines

Cell line source(s)	ATCC (CCL-82.1), Sigma (94030304), ExcellGene SA (HEKExpress XLG1.0)
Authentication	Cell lines were used as received without authentication.
Mycoplasma contamination	Cell lines were not tested for mycoplasma contamination.
Commonly misidentified lines (See ICLAC register)	No commonly misidentified cell lines were used in the study.

Animals and other organisms

Policy information about studies involving animals; ARRIVE guidelines recommended for reporting animal research

Laboratory animals	All mouse lines were established on a C57BL/6 genetic background. Pomc-cre (B6.FVB-Tg(Pomc-cre)1Lowl/J), Lepr-cre (B6.129-Leprtm2(cre)Rck/J), Agrp-cre (STOCK Agrptm1(cre)Lowl/J), Sim1-cre (B6.FVB(129X1)-Tg(Sim1-cre)1Lowl/J), UBC-cre/ERT2 (B6.Cg-Ndor1Tg(UBC-cre/ERT2)1Ejb/1J), and Rosa26-LSL-tdTomato (B6.Cg-Gt(ROSA)26Sortm14(CAG-tdTomato)Hze/J) mice were purchased from The Jackson Laboratory. B6.miR7a1tm1(fl/fl)ms, B6.miR7a2tm1(fl/fl)ms, and B6.miR7btm1(fl/fl)ms were generated in our lab and are previously described (Latreille et al., 2014). All mice were maintained on a C57BL/6 background. Both males and females between 4-25 weeks of age were used for experiments (as indicated). Mice were housed in a pathogen-free animal facility at the Institute of Molecular Health Sciences at ETH Zürich, in a temperature-controlled room (22°C) with 55% humidity and with a 12-hour light/12-hour dark cycle (lights on from 06:00 to 18:00). Mice were fed a standard laboratory chow diet (Kliba Nafag 3437; containing 3 kcal/g metabolizable energy with 4.5% fat, 18.5% protein, and 38% carbohydrate [w/w]) or high-fat diet (HFD) (SAFE diets 260HF; containing 5.5 kcal/g metabolizable energy with 36% fat, 20% protein, 37% carbohydrate, and 18% sucrose [w/w]). Feeding with HFD began at 4-5 weeks of age.
Wild animals	No wild animals were used in the study.
Field-collected samples	No field collected samples were used in the study
Ethics oversight	All animal experiments were approved by the Kantonale Veterinäramt Zürich.

Note that full information on the approval of the study protocol must also be provided in the manuscript.
