# Supplementary material for: MicroRNA-7 regulates melanocortin circuits involved in mammalian energy homeostasis
Source: Nat Commun. 2022 Sep 29;13:5733. doi: 10.1038/s41467-022-33367-w (PMC9522793; doi:10.1038/s41467-022-33367-w)
Supplement: Supplementary file 2 — Peer Review File [file 41467_2022_33367_MOESM2_ESM.pdf]

nature portfolio

Peer Review File

Title: MicroRNA-7 regulates melanocortin circuits involved in mammalian energy homeostasis

Open Access This file is licensed under a Creative Commons Attribution 4.0 International License, which permits use, sharing, adaptation, distribution and reproduction in any medium or format, as long as you give appropriate credit to the original author(s) and the source, provide a link to the Creative Commons license, and indicate if changes were made. In the cases where the authors are anonymous, such as is the case for the reports of anonymous peer reviewers, author attribution should be to 'Anonymous Referee' followed by a clear attribution to the source work. The images or other third party material in this file are included in the article's Creative Commons license, unless indicated otherwise in a credit line to the material. If material is not included in the article's Creative Commons license and your intended use is not permitted by statutory regulation or exceeds the permitted use, you will need to obtain permission directly from the copyright holder. To view a copy of this license, visit http://creativecommons.org/licenses/by/4.0/.

Editorial Note: Parts of this Peer Review File have been redacted as indicated to remove third-party material where no permission to publish could be obtained.

REVIEWER COMMENTS</B>

Reviewer #1 (Remarks to the Author):

LaPierre, et. al. combined mouse studies with human genetic data and provided solid evidence of the involvement of miR-7 in the hypothalamic melanocortin pathway to regulate mammalian energy homeostasis. They pinpointed the hypothalamic Sim neurons as the effect cell type to cause hyperphagia, obesity and increased linear growth, mirroring Sim1 and Melanocortin-4 receptor (MC4R) haplo-insufficiency in mice and humans. In addition, they identified two downstream genes Snca (α-Synuclein) and Igsf8 (Immunoglobulin Superfamily Member 8) as miR-7 target genes that act in Sim1 neurons to regulate body weight and endocrine axes. This is a well-conducted study.
I only have a few comments:
1, page 5, "Hypothalamic miR-7 was regulated by metabolic state, with reduced expression during fasting (Fig. 1e) and increased expression in obese ob/ob mice (Fig. 1f)". No leptin signaling and less MC4R activation is in ob/ob mice and miR is more expressed in ob/ob mice. Does that mean increased expression of miR7 does not "balance out" the hyperphagic effects of lack of leptin signaling in hypothalamus?
2, page 7, "In metabolic cage experiments performed one week after the onset of HFD feeding, Sim1-cre;mir-7fl/fl females exhibited increased food intake (Fig. 2i,j) and RER (Fig. 2k,l) compared to mir-7fl/fl controls." I could not see the changes of RER in Fig. 2k,l?
3, page 12, "As we have found that miR-7 is enriched in Sim1 neurons compared to the rest of the hypothalamus." Although metabolic effects were observed with deletion of mir-7 in Sim1 neurons, I cannot find supporting data of direct comparisons across different neurons.
4, page 13, the author reported genetic associations of variants around HNRNPK with anthropometric and metabolic traits. I am wondering whether there are genetic associations around MIR7-2 (chr15) and MIR7-3 (chr19) with these traits in human?
5, page 14, the author provided solid genetic evidence on genetic regulation of metabolic traits and relevant gene expressions in supplementary table 1 and table 2. Could the author add more information in Supplementary table 2 to integrate trait associations with gene expressions together. for example, for variants, the alleles associated with reduced HNRNPK expressions were associated with increased height, adiposity traits and other traits, etc.

Reviewer #2 (Remarks to the Author):

Previous work from Markus Stoffel's lab showed microRNA-7 (miR-7) contributes to secretory functions in the pituitary gland and pancreas. The current study pursues functions of miR-7 in the brain. More specifically, the authors carried out a genetic screen to understand how miR-7 expression in regions of

the hypothalamus regulates energy homeostasis. The study generates a number of new mouse lines and identifies an important relationship between miR-7 regulation and human variants at the HNRNPK locus that regulate body composition. This is an interesting and timely study that integrates microRNA biology with energy balance.

Experimentally, the experiments address how deletion of miR-7 results in obesity in mice on normal chow and on high-fat diet by cell-specific deletion of miR-7 in neurons expressing Leptin receptor (Lepr), Pro-opiomelanocortin (Pomc), Agouti-related protein (AgRP), and Single-minded homology 1 (Sim1). The authors argue that Sim1-specific miR-7 deletion results in obesity by increasing food intake, decreasing energy expenditure, and increasing insulin secretion. Overall, the results in mice are strong, though it would be nice to see future studies targeting the mechanism behind the sex differences within this phenotype.

-The authors demonstrated the single floxed knocked lines show reduced miR-7 expression in the earlier studies (References 27-29). However, the expression of miR-7 in the hypothalamic regions upon Cre recombination will increase the rigor of the study. Only miR-7 knockout is validated in the Sim1-Cre (Figure 4D). Furthermore, the study explored miR-7b conditional knockouts but this line seems completely new and without validation. In this reviewer's opinion, measurement of miR-7 levels is a critical addition to the study because of the small effect size of nutritional challenge on miR-7 expression (Figure 1E and Figure 1F).

-In Figure 5, the strategy for target nomination requires clarification. The authors reference mRNAs with conserved miR-7 binding sites, but the description of targets remained unaddressed. What defined the conservation of miR-7 targets? Do targets of miR-7 depressed in the conditional knockouts broadly enrich for pathways that act on feeding and/or other energy balance phenotypes?

-The study identified a strong sex difference. Results mostly describe an obesity phenotype in females, but the results do not receive sufficient elaboration or explanation. Are Sim1 neurons important for sex hormones and predominantly affect females? Besides a couple sentences in the discussion, there is not much said about the large sex difference observed.

-More information regarding the implications of Snca and Igsf8 targeting by miR-7 can expand the interpretation. The study does not demonstration a critical role for miR-7 regulation of SNCA and IGSF8 protein levels nor other potential targets.

-In Figure 1, authors show expression of miR-7 in hypothalamic nuclei. The diagram shows miR-7 also highly expressed in suprachiasmatic nuclei (SCN) and supraoptic nucleus (SON). Authors focused on periventricular nucleus (PVN) and arcuate nucleus (ARC) only. Can the authors expand upon a role in the other regions of the brain where miR-7 seems highly expressed?

Reviewer #3 (Remarks to the Author):

This study investigates the physiological role of the miR-7 family of microRNAs as expressed in four different hypothalamic neuronal populations, the Agrp, POMC, Lep-R and Sim-1 neurons. Mice were generated with knockout of the three miR-7s genes in each of these cell types and were phenotyped for various metabolic traits. Inactivation of miR-7 had a significant impact only when performed in Sim-1 neurons of the PVN. The phenotype was more marked in female mice than in male mice and included increased body weight, body length, and higher insulin secretion. This phenotype recapitulated in large part that of the whole body miR-7 knockout mice. Transcript profiling of Sim-1 neurons from control and knockout mice revealed a loss of expression of several key PVN-specific neuropeptide mRNAs and up-regulation of several genes. Overexpression in Sim-1 neurons of several of those by AAV-mediated gene delivery led to the identification of Snca and Igsf8 as mediators of miR-7 action on increased body weight, plasma insulin and Igf1 levels.

This is an extensive and important study that provides compelling evidence for the role of miR-7 in regulating Sim-1 neurons functions. Inactivation of this gene family in PVN neurons has pleiotropic effects on body weight, linear growth, the HPA axis, feeding, and fluid intake. This is in keeping with the reduced expression of most PVN neuropeptides. This study provides convincing data, which fully support an important role of miR-7 in the PVN and which also identifies two genes that mediate at least part of the knockout mouse phenotype.

I have only minor comments:

The two genes, Snca and Igsf8, are overexpressed in all Sim-1-Cre neurons of the PVN. But is it known that they are expressed in all Sim-1 neurons or are they expressed in specific Sim-1 neurons subpopulations that express different neuropeptides? If this is the case one could expect to observe specific phenotypes following their individual overexpression. Is this the case? What about the phenotype of the mice that have been injected with the AAV constructs for overexpression of the other investigated genes? Have all the different PVN-dependent regulatory axis been tested? For instance, corticosterone levels in the knockout mice are very much reduced compared to control mice (Fig 3J). Have the authors tested corticosterone levels following AAV-dependent gene transduction?

LaPierre *et al.*: Response to reviewer comments

We would like to thank all reviewers for taking the time to read our manuscript and for providing constructive comments. We have now added additional experiments and textual changes, which we believe have led to an improved manuscript. Please find below our point-by-point response to the reviewers' comments (with line references corresponding to the revised manuscript Word document in "track changes" mode):

Reviewer #1 (Remarks to the Author):

LaPierre, et. al. combined mouse studies with human genetic data and provided solid evidence of the involvement of miR-7 in the hypothalamic melanocortin pathway to regulate mammalian energy homeostasis. They pinpointed the hypothalamic Sim neurons as the effect cell type to cause hyperphagia, obesity and increased linear growth, mirroring Sim1 and Melanocortin-4 receptor (MC4R) haplo-insufficiency in mice and humans. In addition, they identified two downstream genes Snca (α-Synuclein) and Igsf8 (Immunoglobulin Superfamily Member 8) as miR-7 target genes that act in Sim1 neurons to regulate body weight and endocrine axes. This is a well-conducted study.

I only have a few comments:

1. page 5, "Hypothalamic miR-7 was regulated by metabolic state, with reduced expression during fasting (Fig. 1e) and increased expression in obese ob/ob mice (Fig. 1f)". No leptin signaling and less MC4R activation is in ob/ob mice and miR is more expressed in ob/ob mice. Does that mean increased expression of miR7 does not "balance out" the hyperphagic effects of lack of leptin signaling in hypothalamus?

RESPONSE: We agree with the reviewer's interpretation that increased miR-7 expression is not sufficient to balance out the obesogenic effect of complete leptin deficiency, since *ob/ob* mice gain more weight compared to their *ob/+* littermates. It is possible that the upregulation of miR-7 partially dampens the effects of leptin deficiency. However, to assess this experimentally would require a mouse model with both *ob/ob* and miR-7 knockout alleles to determine whether these mice have a stronger obesity phenotype compared to the *ob/ob* genotype alone. This would be an interesting experiment for further study, but the breedings to achieve this mouse model would take >1 year and are therefore not feasible to complete in the scope of this study. We have added a statement in the revised Discussion to highlight this possible compensatory role of miR-7 (**line 387-389**).

2. page 7, "In metabolic cage experiments performed one week after the onset of HFD feeding, Sim1-cre;mir-7fl/fl females exhibited increased food intake (Fig. 2i,j) and RER (Fig. 2k,l) compared to mir-7fl/fl controls." I could not see the changes of RER in Fig. 2k,l?

RESPONSE: This was an error in the text. It has been corrected to "(Supplementary Fig. 2k,l)" (**line 140**).

3. page 12, “As we have found that miR-7 is enriched in Sim1 neurons compared to the rest of the hypothalamus.” Although metabolic effects were observed with deletion of mir-7 in Sim1 neurons, I cannot find supporting data of direct comparisons across different neurons.

RESPONSE: In the results, we show that miR-7a and miR-7b expression is higher in tdTomato+ neurons (i.e. Sim1 neurons) compared to tdTomato- neurons (i.e. the rest of the hypothalamus) **(Fig. 4d,e)**; we have now added this figure reference in the relevant section of text **(line 257)**. Although we did not measure miR-7 expression in other specific neuron types, the purpose of this statement was to highlight that the previous study only examined the relationship between miR-7 and Cyrano in whole hypothalamus tissue, and may therefore have missed an effect that only becomes apparent when studying a cell population with high miR-7 expression (i.e. Sim1 neurons). We have revised the phrasing of the relevant paragraph to clarify this point **(line 251-259).**

4. page 13, the author reported genetic associations of variants around HNRNPK with anthropometric and metabolic traits. I am wondering whether there are genetic associations around MIR7-2 (chr15) and MIR7-3 (chr19) with these traits in human?

RESPONSE: Unlike *MIR7-1*, *MIR7-2* and *MIR7-3* are not embedded in the introns of protein-coding genes, so it is difficult to examine human variants that affect their expression. Nonetheless, we searched current eQTL resources and recent publications, as summarised below:

PhenomeXcan reports gene/trait correlations for *MIR7-3HG* (“*MIR7-3* Host Gene”) based on GTEx v8 and UK Biobank Rapid GWAS. In multi-tissue analysis, the S-MultiXcan p-values are not significant (predicted trait-expression correlations across tissues) and the Regional Colocalisation Probabilities are small (RCP < 0.1, summarising GWAS/eQTLs colocalisations) for all traits except for one exercise related trait. The smallest S-MultiXcan p-values are fat-free mass traits but these traits have small RCPs [http://apps.hakyimlab.org/phenomexcan/ , gene=MIR7-3HG, S-MultiXcan p-value threshold=0]. In single tissues, PhenomeXcan reports associations of predicted *MIR7-3HG* expression in brain and esophagal tissues with fat-free mass traits but with small effect sizes [http://apps.hakyimlab.org/phenomexcan/ ,PhenomeXcan_SingleTissue, gene=MIR7-3HG].

There was no data for *MIR7-2* in PhenomeXcan.

The FIVEx database of eQTL studies corroborates the *MIR7-3HG* PhenomeXcan analysis of GTEx: one SNP (rs3760955) with three significant *MIR7-3HG* eQTLs in brain tissues (GTEx, ROSMAP, BrainSeq), with no significant cis-eQTLs reported for this SNP for nearby genes **(Fig. R1)**. In Open Targets Genetics, PheWAS fat-free mass traits are reported at this locus but there no eQTL data reported for *MIR7-3HG* (https://genetics.opentargets.org/variant/19_4772190_A_C).

We searched two miRNA eQTL papers: (a) Huan *et al.,* (2015) [1]; and (b) Williamson *et al.,* (2015) [2].

a) Huan *et al.,* (2015) [1] reports five cis-eQTLs for *MIR7-3* (FDR<0.1; SupplData4, miR_7_5p). The most significant of these eQTLs (rs12710081) is nominally associated with leptin levels **(Table R1)** among Open Targets Genetics curated GWAS measurement traits. The direction of this effect is decreased *MIR7-3HG* (alt allele A, allele frequency = 15%) being associated with increased leptin levels. This association has a large p-value (p=0.0016), is not GWAS-wide

significant, is from a small cohort (n~1300), and the relation between quantitative leptin levels and other physiological traits is unclear and has unknown relevance. Therefore, this association would require further investigation.

b) Williamson *et al.,* (2015) [2] reported brain mir-eQTLs enriched for schizophrenia and bipolar disorder association signals. They report a mir7-1 eQTL ("mir-7-1-3p" in Table 1 of Williamson *et al*). This lead variant is reported to be associated with worry and neuroticism traits in Open Targets Genetics [https://genetics.opentargets.org/variant/9_82984665_C_T], not with height or impedance traits, and is not in LD with the variants we describe in Figure 6. Their paper does not report *MIR7-2* or *MIR7-3* eQTLs at their given reporting thresholds.

Taken together, current data supports a possible association between *MIR7-3* expression in brain tissues and fat-free mass traits. However, the reported associations are modest and require further investigation and validation at these loci so we have not reported these preliminary observations in the manuscript.

[redacted]

[redacted]

[redacted]

5. page 14, the author provided solid genetic evidence on genetic regulation of metabolic traits and relevant gene expressions in supplementary table 1 and table 2. Could the author add more information in Supplementary table 2 to integrate trait associations with gene expressions together. for example, for variants, the alleles associated with reduced HNRNPK expressions were associated with increased height, adiposity traits and other traits, etc.

RESPONSE: We recognise that additional clarity would be helpful here. We have now tried to present the information in a way that is both understandable and allows interested readers to perform a deep-dive into the data underlying these associations.

In Supplementary Data 2, we added PhenomeXcan's reported fastENLOC colocalisation signals in each tissue-level datasheet **(Supplementary Data 2C,D,E,H,K-L)**, a new datasheet containing the reported SNP-level colocalisation results **(Supplementary Data 2M)**, and a new datasheet containing the SNPs used by PredictDB's expression prediction step **(Supplementary Data 2N)**.

We chose to provide variant-level URLs that link-out to the Open Targets Genetics (OTG) browser **(Supplementary Data 2M column J; Supplementary Data 2N column J)**. The OTG variant-level resource is easily browsable for GWAS and eQTL directions of effect at individually significant loci (GTEx and non-GTEx eQTL studies). In addition, at each variant, OTG facilitates rapid exploration of pleiotropy (multiple GWAS traits) and regulatory pleiotropy (eQTL variants associated with more than one gene).

OTG uses different approaches and reporting thresholds than our inspection of PhenomeXcan's GTEx v8 analysis (Supplementary Data 2), and individually weak tissue-level eQTL signals from GTEx are not displayed in OTG. However, OTG displays individually significant eQTLs from GTEx and other eQTL studies, and we found that these studies tend to corroborate the GTEx-based PhenomeXcan estimates of directionality. GWAS/eQTL directions of effect at the variant level (inputs for PhenomeXcan's fastENLOC colocalisation step) were not readily available from the PhenomeXcan resource in a suitable format.

We also took the opportunity to more clearly highlight the limitations of current data by adding a statement in the revised Discussion **(line 452-459)**.

Reviewer #2 (Remarks to the Author):

Previous work from Markus Stoffel's lab showed microRNA-7 (miR-7) contributes to secretory functions in the pituitary gland and pancreas. The current study pursues functions of miR-7 in the brain. More specifically, the authors carried out a genetic screen to understand how miR-7 expression in regions of the hypothalamus regulates energy homeostasis. The study generates a number of new mouse lines and identifies an important relationship between miR-7 regulation and human variants at the HNRNPK locus that regulate body composition. This is an interesting and timely study that integrates microRNA biology with energy balance.

Experimentally, the experiments address how deletion of miR-7 results in obesity in mice on normal chow and on high-fat diet by cell-specific deletion of miR-7 in neurons expressing Leptin receptor (Lepr), Pro-opiomelanocortin (Pomc), Agouti-related protein (AgRP), and Single-minded homology 1 (Sim1). The authors argue that Sim1-specific miR-7 deletion results in obesity by increasing food intake, decreasing energy expenditure, and increasing insulin secretion. Overall, the results in mice are strong, though it would be nice to see future studies targeting the mechanism behind the sex differences within this phenotype.

1. The authors demonstrated the single floxed knocked lines show reduced miR-7 expression in the earlier studies (References 27-29). However, the expression of miR-7 in the hypothalamic regions upon Cre recombination will increase the rigor of the study. Only miR-7 knockout is validated in the Sim1-Cre (Figure 4D). Furthermore, the study explored miR-7b conditional knockouts but this line seems completely new and without validation. In this reviewer's opinion, measurement of miR-7 levels is a critical addition to the study because of the small effect size of nutritional challenge on miR-7 expression (Figure 1E and Figure 1F).

RESPONSE: As the reviewer pointed out, our original manuscript only presented miR-7a and miR-7b expression data in sorted Sim1 neurons **(Fig. 4d,e)**. Since each cell population (Sim1+, Pomc+, Lepr+, and Agrp+) represents only a small percentage of cells in the hypothalamus, it is not possible to detect the cell-type specific depletion of miR-7 by qPCR of the whole hypothalamus. For example, miR-7a and miR-7b levels are not significantly different in the whole hypothalamus of *miR-7*$^{fl/fl}$ versus *Sim1-cre;miR-7*$^{fl/fl}$ mice (**Figure R2)**, despite the nearly complete depletion of miR-7a and miR-7b from Sim1 neurons as shown in Figures 4d and 4e. To demonstrate the efficient knockout of all three conditional miR-7 alleles in the hypothalamus, we have added miR-7 expression data from *miR-7*$^{fl/fl}$ and *UBC-cre;miR-7*$^{fl/fl}$ mice, which have a global miR-7 knockout, to the revised manuscript (**Supplementary Fig. 1a**). Measuring miR-7 expression levels in the other conditional mouse lines would require extensive breedings and experimental work, as this would involve crossing a tdTomato reporter allele into each conditional knockout line (a total of 5 alleles in each mouse line, requiring >1 year of breeding), and performing cell sorting on isolated neurons to measure cell type-specific miR-7 expression. Since the Cre mouse lines used in this study are highly validated [3–17] and the miR-7 floxed alleles show efficient recombination by UBC-cre (**Supplementary Fig. 1a**) and Sim1-cre **(Fig. 4d,e)**, we do not believe it is essential to measure cell-type specific miR-7 expression in the other conditional mouse lines.

Regarding the reviewer's concerns about the validation of the miR-7b conditional knockout line, the miR-7b floxed allele used in this study has been described and validated in our previous publication by Ahmed *et al.* [18] (as stated in the Methods section; **line 482-483**). Figures S1A-S1D in Ahmed *et al.* show

that the miR-7b floxed allele was recombined by CMV-cre to generate global miR-7b$^{-/-}$ mice, which display a loss of miR-7b (but not miR-7a) expression [18]. We have also validated this miR-7b floxed allele by showing miR-7b depletion upon recombination by UBC-cre (**Supplementary Fig. 1a**) and Sim1-cre (**Fig. 4d,e**).

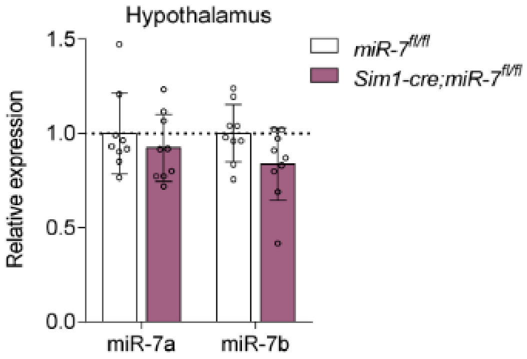

Figure R2. miR-7a and miR-7b expression in the hypothalamus of *miR-7$^{fl/fl}$* and *Sim1-cre;miR-7$^{fl/fl}$* mice.

2. In Figure 5, the strategy for target nomination requires clarification. The authors reference mRNAs with conserved miR-7 binding sites, but the description of targets remained unaddressed. What defined the conservation of miR-7 targets? Do targets of miR-7 depressed in the conditional knockouts broadly enrich for pathways that act on feeding and/or other energy balance phenotypes?

RESPONSE: To identify conserved miR-7 target genes, we used TargetScanMouse release 7.2 to extract a list of genes containing a conserved 8mer, 7mer-m8, or 7mer-A1 site for miR-7 in their 3'UTR. The criteria for target site conservation in TargetScanMouse7.2 is defined by phylogenetic branch length, with each site type having a different threshold for conservation (8mer ≥ 0.6; 7mer-m8 ≥ 1.8; 7mer-1A ≥ 2.5) [19]. We have added this information to the revised manuscript **(line 263-264 in Results; line 598-601 in Methods).**

As suggested by the reviewer, we attempted to identify pathway enrichment patterns within the regulated target genes, but this was unsuccessful. Using our cutoff of FDR <0.05 and fold-change >1.3, we only detected 22 upregulated target genes in this study **(Fig. 5 c,d)**. We used the PANTHER Overrepresentation Test tool to examine pathway enrichment among these 22 genes in several categories/pathways (GO Biological Process, GO Molecular Function, GO Cellular component, PANTHER pathways, Reactome pathways). We did not detect any significant enrichment in these analyses, which is to be expected given the small number of analysed genes.

3. The study identified a strong sex difference. Results mostly describe an obesity phenotype in females, but the results do not receive sufficient elaboration or explanation. Are Sim1 neurons important for sex hormones and predominantly affect females? Besides a couple sentences in the discussion, there is not much said about the large sex difference observed.

RESPONSE: We agree that the sexual dimorphism of the phenotype is interesting and warrants further elaboration in the manuscript. In order to emphasize these sex-specific effects of miR-7, we have added new figures to the revised manuscript highlighting that miR-7 in Sim1 neurons is required to maintain the sexual dimorphism of body weight in mice (**Fig. 2g,h; line 129-133 of Results section**). We have also added a statement in the discussion to elaborate on possible mechanisms underlying this sex-specific effect. In addition to the fact that Sim1 neurons *per se* seem to have a greater role in females, there is also a possibility that miR-7 plays a role in the protective effects of estrogens on diet-induced obesity **(line 390-403)**.

4. More information regarding the implications of Snca and Igsf8 targeting by miR-7 can expand the interpretation. The study does not demonstration a critical role for miR-7 regulation of SNCA and IGSF8 protein levels nor other potential targets.

RESPONSE: In mammals, it is well established that miRNAs predominantly regulate their target protein levels through destabilization and downregulation of their mRNA; as such, regulation of target mRNA expression and protein translation is tightly correlated [20]. In our original manuscript, we measured target gene mRNA regulation by RNA sequencing of sorted Sim1 neurons. However, as the reviewer pointed out, we did not experimentally demonstrate that miR-7 also regulates the protein levels of α-synuclein, IgSF8, and other targets.

With only ~3000 Sim1 neurons per mouse, this cell population represents only a small fraction of cells in the hypothalamus (approximately 1-2% of hypothalamic cells, as measured in our FACS sorting experiments). As a result, detecting miR-7 target protein regulation in the hypothalamus of *Sim1-cre;miR-7$^{fl/fl}$* mice would be methodologically challenging. To address whether miR-7 regulates both the mRNA and protein levels of its targets, we have therefore manipulated miR-7 levels in the SH-SY5Y neuronal cell line to examine the mRNA and protein expression of representative targets that were regulated by miR-7 in Sim1 neurons.

By overexpressing miR-7 to the same level as in the mouse hypothalamus (**Supplementary Fig. 8a**), we found that miR-7 regulates its target mRNA and protein levels in a consistent manner. For example, *Igsf8*/IgSF8 shows the strongest regulation of both mRNA and protein levels, while *Snca*/α-synuclein and *Raf1*/c-Raf show intermediate effects, and *Arrb1*/β-arrestin shows the weakest regulation of both mRNA and protein expression by miR-7 (**Supplementary Fig. 8b-d**). These new data have been included in the revised version of the manuscript (**line 269-273 in Results section**)

5. In Figure 1, authors show expression of miR-7 in hypothalamic nuclei. The diagram shows miR-7 also highly expressed in suprachiasmatic nuclei (SCN) and supraoptic nucleus (SON). Authors focused on periventricular nucleus (PVN) and arcuate nucleus (ARC) only. Can the authors expand upon a role in the other regions of the brain where miR-7 seems highly expressed?

Indeed, miR-7 is highly expressed in the ARC, PVN, SCN, and SON [21]. As the goal of our study was to investigate the obesity phenotype of mice lacking miR-7, we focused on the hypothalamic nuclei with a stronger link to energy homeostasis (i.e. the ARC and PVN). Nonetheless, the high expression of miR-7 in the SCN and SON suggests that it may also play important roles in these brain regions.

The SCN is the circadian pacemaker in mammals; it controls behavioural, neuroendocrine, and autonomic functions to coordinate the daily rhythm of neural and metabolic processes [23]. As disruption of SCN function leads to metabolic dysfunction and obesity [24–26], it is possible that this brain region accounts for the stronger obesity phenotype of *UBC-cre/ERT2;miR-7*$^{fl/fl}$ mice compared to *Sim1-cre;miR-7*$^{fl/fl}$ mice. Future studies investigating the role of miR-7 in the SCN (e.g. using Syt10-cre [27] or Six3-cre [28]) may reveal important roles of miR-7 in regulating circadian control of energy homeostasis and other physiological processes. We have added a statement in the revised Discussion addressing a possible role of miR-7 in the SCN **(line 407-415)**.

The SON is comprised of magnocellular OXT- and AVP-expressing neurons that release these neuropeptides into circulation through axon terminals in the posterior pituitary. Sim1 is expressed in neurons of both the PVN and SON, as these two populations arise from a common developmental lineage [3,22]. Therefore, miR-7 is also deleted in the SON of *Sim1-cre;miR-7*$^{fl/fl}$ mice and it is possible that phenotypes specifically related to circulating OXT and AVP (e.g. water intake) are partially caused by the loss of miR-7 in the SON. We have added this statement to the revised Discussion **(line 415-420)**. This also brings up an important point for the interpretation of our RNA sequencing data in sorted Sim1 neurons: as we were aware of *Sim1-cre* expression in the SON, we dissected the hypothalamus in a manner that included the entire PVN region and excluded the SON region in order to focus our analysis on the PVN. This detail was already shown in the experimental schematic (**Fig. 4c)**, and we have now added it to the revised Methods section (**line 565-567**).

Reviewer #3 (Remarks to the Author):

This study investigates the physiological role of the miR-7 family of microRNAs as expressed in four different hypothalamic neuronal populations, the Agrp, POMC, Lep-R and Sim-1 neurons. Mice were generated with knockout of the three miR-7s genes in each of these cell types and were phenotyped for various metabolic traits. Inactivation of miR-7 had a significant impact only when performed in Sim-1 neurons of the PVN. The phenotype was more marked in female mice than in male mice and included increased body weight, body length, and higher insulin secretion. This phenotype recapitulated in large part that of the whole body miR-7 knockout mice. Transcript profiling of Sim-1 neurons from control and knockout mice revealed a loss of expression of several key PVN-specific neuropeptide mRNAs and up-regulation of several genes. Overexpression in Sim-1 neurons of several of those by AAV-mediated gene delivery led to the identification of Snca and Igsf8 as mediators of miR-7 action on increased body weight, plasma insulin and Igf1 levels.

This is an extensive and important study that provides compelling evidence for the role of miR-7 in regulating Sim-1 neurons functions. Inactivation of this gene family in PVN neurons has pleiotropic effects on body weight, linear growth, the HPA axis, feeding, and fluid intake. This is in keeping with the reduced expression of most PVN neuropeptides. This study provides convincing data, which fully support an important role of miR-7 in the PVN and which also identifies two genes that mediate at least part of the knockout mouse phenotype.

I have only minor comments:

The two genes, Snca and Igsf8, are overexpressed in all Sim-1-Cre neurons of the PVN. But is it known that they are expressed in all Sim-1 neurons or are they expressed in specific Sim-1 neurons subpopulations that express different neuropeptides? If this is the case one could expect to observe specific phenotypes following their individual overexpression. Is this the case? What about the phenotype of the mice that have been injected with the AAV constructs for overexpression of the other investigated genes? Have all the different PVN-dependent regulatory axis been tested? For instance, corticosterone levels in the knockout mice are very much reduced compared to control mice (Fig 3J). Have the authors tested corticosterone levels following AAV-dependent gene transduction?

RESPONSE: To determine whether the candidate miR-7 target genes are differentially expressed across the subpopulations that express different neuropeptides, we analysed an existing dataset of single-cell RNA sequencing of adult mouse hypothalamus, which identified 45 transcriptionally distinct cell clusters in the hypothalamus [29]. After filtering for cells in putative PVN neuron clusters (Glu1, Glu5, Glu7, Glu8, Glu10, Glu14, Glu15), there were 105 *Avp*+, 82 *Oxt*+, 26 *Crh*+, 23 *Sst*+, and 77 *Trh*+ cells for target gene expression analysis in subpopulations that express different neuropeptides. Using a Fisher's exact test for co-occurrence, we found that *Snca* is expressed in significantly fewer *Avp*+, *Oxt*+, and *Crh*+ neurons than would be expected by random sampling, but it is nonetheless present in a substantial fraction of these cells **(Fig. R3a)**. Otherwise, no significant enrichment or depletion was observed for target genes in specific neuropeptide-expressing cells. Of note, the low sequencing depth of single-cell RNA sequencing can result in a "dropout", i.e. failure to detect transcripts with low expression values [30]. The depletion of *Snca* from specific cell types could therefore be attributed either to a lower fraction of cells expressing *Snca*, and/or to a lower expression of *Snca* in those cells. Within double-positive cells, target gene expression levels were not significantly different across neuropeptide-expressing cell types **(Fig. R3b)**. Based on these analyses, it is possible that *Snca* plays a slightly less important role in *Avp*+, *Oxt*+, and *Crh*+ cells compared to the other cell types. Nonetheless, as deletion of miR-7 leads to a de-repression of target genes, the upregulation of Snca may still have biologically meaningful effects in these cells despite a low baseline expression. As these results are derived from a small number of cells, we do not consider the data robust enough for publication and we have therefore provided this information for reviewers only. As no study to date has performed single-cell RNA sequencing of PVN neurons specifically, it would be informative to perform this analysis on sorted Sim1+ neurons from *Sim1-cre;LSL-tdTomato* mice. The resulting data would provide valuable depth of information on the transcriptional profiles of specific PVN neuron subpopulations. We believe this extensive analysis is outside the scope of this manuscript but will be an important topic that we are excited to pursue in a future study.

To address the reviewer's question on the regulation of other PVN-dependent axes controlled by specific subtypes of Sim1 neurons (e.g. corticosterone levels), we have generated new cohorts of mice with overexpression of *Snca* or *Igsf8* in Sim1 neurons. In these cohorts, we measured additional physiological parameters that were regulated in *Sim1-cre;miR-7$^{fl/fl}$* mice: plasma corticosterone, water intake, food intake, and energy expenditure. Corticosterone levels and water intake were unchanged upon overexpression of *Snca* or *Igsf8* (**Supplementary Fig. 8r–u**), suggesting that the function of CRH- and AVP-expressing Sim1 neurons was not impaired in these mice. Food intake was slightly increased in

mice with *Igsf8* overexpression (**Supplementary Fig. 8v,w**), while energy expenditure was slightly reduced in mice with *Snca* overexpression (**Supplementary Fig. 8x,y**), Taken together, overexpression of *Snca* and *Igsf8* in Sim1 neurons each partly recapitulates the phenotype of *Sim1-cre;mir-7*$^{fl/fl}$ mice. These new data have been included in the revised version of the manuscript (**line 298-304 in Results section**) and we have added a statement in the Discussion addressing the possible mechanisms underlying these partial phenotypes upon overexpression of *Snca* or *Igsf8* **(line 439-443)**.

To address the reviewer's questions regarding additional phenotypes of the mice injected with AAV constructs overexpressing the other investigated target genes, we have performed additional measurements of plasma IGF-1 in the other 5 cohorts of mice, as this parameter was significantly upregulated upon overexpression of *Snca* and *Igsf8*. Plasma IGF-1 was unchanged after Sim1 neuron-specific overexpression of *Spata2*, *Arrb1*, *Fndc4*, *Pole4*, or *Smim12,* further confirming the specificity of *Snca* and *Igsf8* as the effector targets of miR-7 (**Supplementary Fig. 8l-p)**. These new data have been added to the revised manuscript (**line 294-295 in Results)**. Fasting insulin levels were also significantly upregulated in mice with overexpression of *Snca* and *Igsf8*. Unfortunately, we only collected fasted plasma samples from the two cohorts that displayed a body weight phenotype (AAV-*Snca* and AAV-*Igsf8*), and therefore we do not have comparable samples for this analysis in the other 5 cohorts. Nonetheless, we measured insulin levels in the non-fasted plasma of these cohorts and found no significant differences upon overexpression of any of the 5 targets (**Fig. R4a-e**). However, the insulin values were quite elevated and variable compared to the AAV-*Snca* and AAV-*Igsf8* cohorts, as expected given the non-fasted state of the mice. We have not included these insulin measurements in the revised manuscript, as we do not feel it is appropriate to compare insulin levels between fasted and non-fasted measurements across cohorts. Of note, the original manuscript was lacking the detail that insulin was measured specifically in fasted plasma; we have now added this information to the revised manuscript (**line 524-525 in Methods**).

The reviewer's comment highlights the interesting point that only *Snca* and *Igsf8*, and not the other 5 tested targets, had any detectable role in Sim1 neurons. The fact that, among all investigated targets, only *Snca* and *Igsf8* have a detectable function in Sim1 neurons may be related to their enriched expression in these cells; we have added new data to the revised manuscript showing that *Snca* and *Igsf8* display the highest baseline expression out of the 22 upregulated targets in Sim1 neurons (**Supplementary Fig. 8q; line 295-298 in Results**).

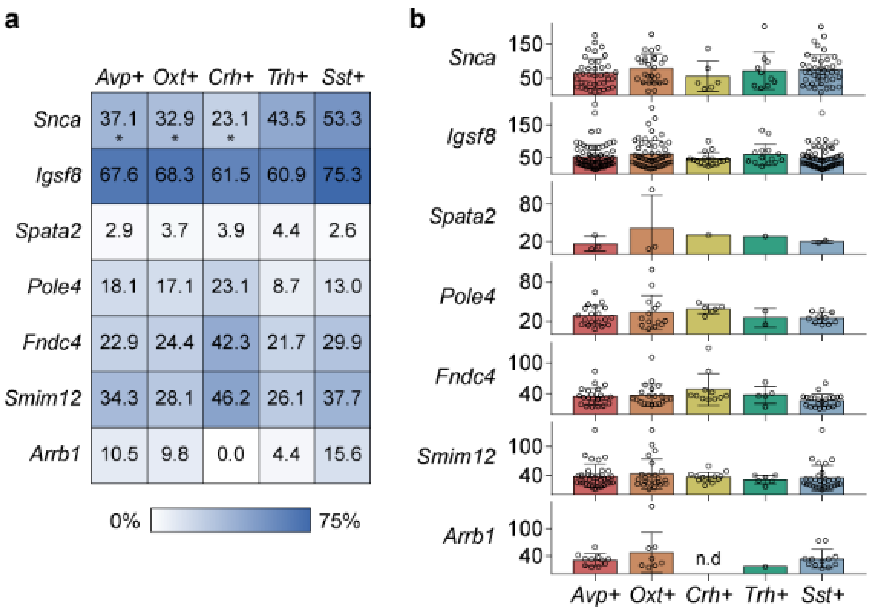

Figure R3. Target gene expression in neuropeptide-defined neuron subpopulations.

(a) Heat map illustrating the percentage of neurons in neuropeptide-defined subpopulations that express selected miR-7 target genes. *FDR < 0.05, Fisher's exact test. **(b)** Target gene expression (in RPM) in neuropeptide-defined neuron subpopulations.

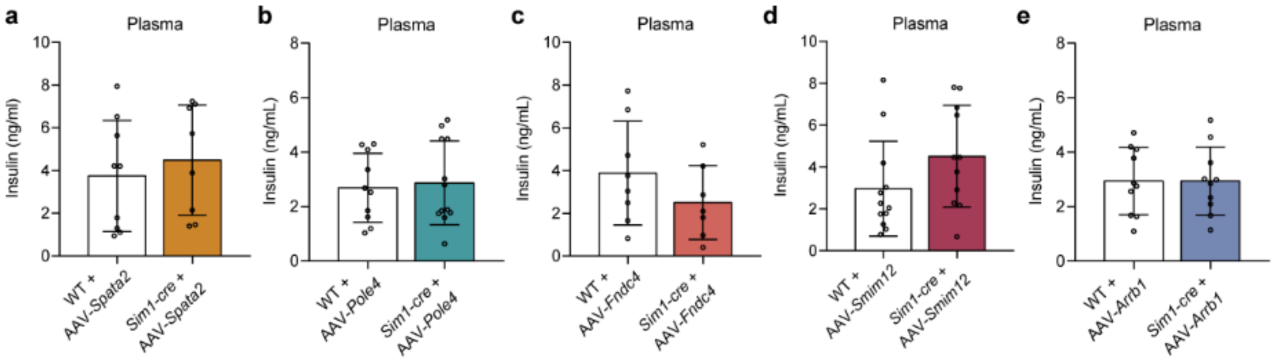

Figure R4. Plasma insulin levels upon overexpression of miR-7 targets *Spata2* (a), *Pole4* (b), *Fndc4* (c), *Smim12* (d), and *Arrb1* (e).

References

1. Huan, T. *et al.* Genome-wide identification of microRNA expression quantitative trait loci. *Nat. Commun.* **6,** 6601 (2015).
2. Williamson, V. S. *et al.* Expression quantitative trait loci (eQTLs) in microRNA genes are enriched for schizophrenia and bipolar disorder association signals. *Psychol. Med.* **45,** 2557–2569 (2015).
3. Balthasar, N. *et al.* Divergence of melanocortin pathways in the control of food intake and energy

expenditure. *Cell* **123,** 493–505 (2005).

4. McHugh, T. J. *et al.* Dentate Gyrus NMDA Receptors Mediate Rapid Pattern Separation in the Hippocampal Network. *Science* **317,** 94–99 (2007).

5. DeFalco, J. *et al.* Virus-assisted mapping of neural inputs to a feeding center in the hypothalamus. *Science* **291,** 2608–2613 (2001).

6. Tong, Q., Ye, C.-P., Jones, J. E., Elmquist, J. K. & Lowell, B. B. Synaptic release of GABA by AgRP neurons is required for normal regulation of energy balance. *Nat. Neurosci.* **11,** 998–1000 (2008).

7. Ruzankina, Y. *et al.* Deletion of the Developmentally Essential Gene ATR in Adult Mice Leads to Age-Related Phenotypes and Stem Cell Loss. *Cell Stem Cell* **1,** 113–126 (2007).

8. Campbell, J. N. *et al.* A molecular census of arcuate hypothalamus and median eminence cell types. *Nat. Neurosci.* **20,** 484–496 (2017).

9. Órdenes, P. *et al.* Lactate activates hypothalamic POMC neurons by intercellular signaling. *Sci. Rep.* **11,** 1–12 (2021).

10. Wei, Q. *et al.* Uneven balance of power between hypothalamic peptidergic neurons in the control of feeding. *Proc. Natl. Acad. Sci. U. S. A.* **115,** E9489–E9498 (2018).

11. Zhang, K. X. *et al.* Violet-light suppression of thermogenesis by opsin 5 hypothalamic neurons. *Nature* **585,** 420–425 (2020).

12. Kohno, D. *et al.* Dnmt3a in Sim1 Neurons Is Necessary for Normal Energy Homeostasis. *J. Neurosci.* **34,** 15288–15296 (2014).

13. Kim, E. R. *et al.* Paraventricular hypothalamus mediates diurnal rhythm of metabolism. *Nat. Commun.* **11,** 3794 (2020).

14. Krashes, M. J. *et al.* An excitatory paraventricular nucleus to AgRP neuron circuit that drives hunger. *Nature* **507,** 238–242 (2014).

15. Caglar, C. & Friedman, J. Restriction of food intake by PPP1R17-expressing neurons in the DMH. *Proc. Natl. Acad. Sci.* **118,** e2100194118 (2021).

16. Lu, J. *et al.* Spata7 is required for maintenance of the retinal connecting cilium. *Sci. Rep.* **12,** 5575 (2022).

17. Huo, Y. *et al.* Tumor suppressor PALB2 maintains redox and mitochondrial homeostasis in the brain and cooperates with ATG7/autophagy to suppress neurodegeneration. *PLOS Genet.* **18,** e1010138 (2022).

18. Ahmed, K. *et al.* Loss of microRNA-7a2 induces hypogonadotropic hypogonadism and infertility. *J. Clin. Invest.* **127,** 1061–1074 (2017).

19. Agarwal, V., Bell, G. W., Nam, J.-W. & Bartel, D. P. Predicting effective microRNA target sites in mammalian mRNAs. *Elife* **4,** e05005 (2015).

20. Guo, H., Ingolia, N. T., Weissman, J. S. & Bartel, D. P. Mammalian microRNAs predominantly act to decrease target mRNA levels. *Nature* **466,** 835–840 (2010).

21. Herzer, S., Silahtaroglu, A. & Meister, B. Locked Nucleic Acid-Based In Situ Hybridisation Reveals miR-7a as a Hypothalamus-Enriched MicroRNA with a Distinct Expression Pattern. *J. Neuroendocrinol.* **24,** 1492–1504 (2012).

22. Michaud, J. L., Rosenquist, T., May, N. R. & Fan, C. M. Development of neuroendocrine lineages requires the bHLH-PAS transcription factor SIM1. *Genes Dev.* **12,** 3264–3275 (1998).

23. Hastings, M. H., Maywood, E. S. & Brancaccio, M. Generation of circadian rhythms in the suprachiasmatic nucleus. *Nat. Rev. Neurosci.* **19,** 453–469 (2018).

24. Adlanmerini, M. *et al.* REV-ERB nuclear receptors in the suprachiasmatic nucleus control circadian period and restrict diet-induced obesity. *Sci. Adv.* **7,** 1–13 (2021).

25. Grippo, R. M. *et al.* Dopamine Signaling in the Suprachiasmatic Nucleus Enables Weight Gain Associated with Hedonic Feeding. *Curr. Biol.* **30,** 196–208.e8 (2020).

26. Coomans, C. P. *et al.* The suprachiasmatic nucleus controls circadian energy metabolism and hepatic insulin sensitivity. *Diabetes* **62,** 1102–1108 (2013).

27. Husse, J., Zhou, X., Shostak, A., Oster, H. & Eichele, G. Synaptotagmin10-cre, a driver to disrupt clock genes in the SCN. *J. Biol. Rhythms* **26,** 379–389 (2011).

28. Bedont, J. L. *et al.* Lhx1 Controls Terminal Differentiation and Circadian Function of the Suprachiasmatic Nucleus. *Cell Rep.* **7,** 609–622 (2014).

29. Chen, R., Wu, X., Jiang, L. & Zhang, Y. Single-Cell RNA-Seq Reveals Hypothalamic Cell Diversity. *Cell Rep.* **18,** 3227–3241 (2017).

30. Haque, A., Engel, J., Teichmann, S. A. & Lönnberg, T. A practical guide to single-cell RNA-sequencing for biomedical research and clinical applications. *Genome Med.* **9,** 75 (2017).

REVIEWERS' COMMENTS

Reviewer #1 (Remarks to the Author):

I am very satisfied with the authors' responses to my questions and the revisions they made. I have no other concerns. I recommend that the revised paper be accepted. Thank you. Yingchang Lu

Reviewer #2 (Remarks to the Author):

The authors addressed my concerns and I find the revised manuscript strongly improved. Nice work.

Reviewer #3 (Remarks to the Author):

The authors have adequately answered my comments and I have no additional concerns.

LaPierre *et al.*: Response to reviewer comments

Reviewer #1 (Remarks to the Author):
I am very satisfied with the authors' responses to my questions and the revisions they made. I have no other concerns. I recommend that the revised paper be accepted. Thank you. Yingchang Lu

RESPONSE: We thank the reviewer for taking the time to evaluate our revised manuscript.

Reviewer #2 (Remarks to the Author):
The authors addressed my concerns and I find the revised manuscript strongly improved. Nice work.

RESPONSE: We thank the reviewer for taking the time to evaluate our revised manuscript.

Reviewer #3 (Remarks to the Author):
The authors have adequately answered my comments and I have no additional concerns.

RESPONSE: We thank the reviewer for taking the time to evaluate our revised manuscript.
